# Bestrophin-like protein 4 is involved in photosynthetic acclimation to light fluctuations in Chlamydomonas

**DOI:** 10.1093/plphys/kiae450

**Published:** 2024-09-06

**Authors:** Liat Adler, Chun Sing Lau, Kashif M Shaikh, Kim A van Maldegem, Alex L Payne-Dwyer, Cecile Lefoulon, Philipp Girr, Nicky Atkinson, James Barrett, Tom Z Emrich-Mills, Emilija Dukic, Michael R Blatt, Mark C Leake, Gilles Peltier, Cornelia Spetea, Adrien Burlacot, Alistair J McCormick, Luke C M Mackinder, Charlotte E Walker

**Affiliations:** Institute of Molecular Plant Sciences, School of Biological Sciences, University of Edinburgh, Edinburgh EH9 3BF, UK; Centre for Engineering Biology, University of Edinburgh, Edinburgh EH9 3BF, UK; Department of Plant Biology, Division of Biosphere Science and Engineering, Carnegie Science, Stanford, CA 94305, USA; Centre for Novel Agricultural Products (CNAP), Department of Biology, University of York, York YO10 5DD, UK; Department of Biological and Environmental Sciences, University of Gothenburg, Gothenburg 40530, Sweden; Department of Biological and Environmental Sciences, University of Gothenburg, Gothenburg 40530, Sweden; Centre for Novel Agricultural Products (CNAP), Department of Biology, University of York, York YO10 5DD, UK; School of Physics, Engineering and Technology, University of York, York YO10 5DD, UK; Laboratory of Plant Physiology and Biophysics, Bower Building, University of Glasgow, Glasgow G12 8QQ, UK; Centre for Novel Agricultural Products (CNAP), Department of Biology, University of York, York YO10 5DD, UK; Institute of Molecular Plant Sciences, School of Biological Sciences, University of Edinburgh, Edinburgh EH9 3BF, UK; Centre for Engineering Biology, University of Edinburgh, Edinburgh EH9 3BF, UK; Centre for Novel Agricultural Products (CNAP), Department of Biology, University of York, York YO10 5DD, UK; Centre for Novel Agricultural Products (CNAP), Department of Biology, University of York, York YO10 5DD, UK; Department of Biological and Environmental Sciences, University of Gothenburg, Gothenburg 40530, Sweden; Laboratory of Plant Physiology and Biophysics, Bower Building, University of Glasgow, Glasgow G12 8QQ, UK; Centre for Novel Agricultural Products (CNAP), Department of Biology, University of York, York YO10 5DD, UK; School of Physics, Engineering and Technology, University of York, York YO10 5DD, UK; Aix-Marseille Université, CEA, CNRS, Institut de Biosciences et Biotechnologies Aix-Marseille, CEA Cadarache, Saint-Paul-lez-Durance 13108, France; Department of Biological and Environmental Sciences, University of Gothenburg, Gothenburg 40530, Sweden; Department of Plant Biology, Division of Biosphere Science and Engineering, Carnegie Science, Stanford, CA 94305, USA; Department of Biology, Stanford University, Stanford, CA 94305, USA; Institute of Molecular Plant Sciences, School of Biological Sciences, University of Edinburgh, Edinburgh EH9 3BF, UK; Centre for Engineering Biology, University of Edinburgh, Edinburgh EH9 3BF, UK; Centre for Novel Agricultural Products (CNAP), Department of Biology, University of York, York YO10 5DD, UK; Centre for Novel Agricultural Products (CNAP), Department of Biology, University of York, York YO10 5DD, UK

## Abstract

In many eukaryotic algae, CO_2_ fixation by Rubisco is enhanced by a CO_2_-concentrating mechanism, which utilizes a Rubisco-rich organelle called the pyrenoid. The pyrenoid is traversed by a network of thylakoid membranes called pyrenoid tubules, which are proposed to deliver CO_2_. In the model alga Chlamydomonas (*Chlamydomonas reinhardtii*), the pyrenoid tubules have been proposed to be tethered to the Rubisco matrix by a bestrophin-like transmembrane protein, BST4. Here, we show that BST4 forms a complex that localizes to the pyrenoid tubules. A Chlamydomonas mutant impaired in the accumulation of BST4 (*bst4*) formed normal pyrenoid tubules, and heterologous expression of BST4 in Arabidopsis (*Arabidopsis thaliana*) did not lead to the incorporation of thylakoids into a reconstituted Rubisco condensate. Chlamydomonas *bst4* mutants did not show impaired growth under continuous light at air level CO_2_ but were impaired in their growth under fluctuating light. By quantifying the non-photochemical quenching (NPQ) of chlorophyll fluorescence, we propose that *bst4* has a transiently lower thylakoid lumenal pH during dark-to-light transition compared to control strains. We conclude that BST4 is not a tethering protein but is most likely a pyrenoid tubule ion channel involved in the ion homeostasis of the lumen with particular importance during light fluctuations.

## Introduction

Maintaining improvement in crop yields to keep pace with the rising demands for food is becoming increasingly challenging (Horton et al. 2021). Current models predict that an increase in food supply between 35% and 56% from 2010 to 2050 is required (van Dijk et al. 2021). A possible solution to overcome this challenge is engineering a biophysical CO_2_-concentrating mechanism (CCM) into C3 crop plants, which has been proposed to improve crop yields by between 8% and 60%, as well as water-use and nitrogen-use efficiency (Price et al. 2013; McGrath and Long 2014; Long et al. 2019; Fei et al. 2022; Wu et al. 2023). The biophysical CCMs in algae typically function by concentrating CO_2_ into a liquid–liquid phase separated microcompartment called a pyrenoid, which is predominantly made up of a Ribulose-1,5-bisphosphate carboxylase/oxygenase (Rubisco)-rich matrix. This raises the [CO_2_]:[O_2_] ratio around the primary CO_2_-fixing enzyme Rubisco, which brings Rubisco closer to its maximal carboxylation rate and minimizes the competing oxygenation reaction. Chlamydomonas *(Chlamydomonas reinhardtii)* has the most well-understood pyrenoid-based CCM and has become the blueprint for engineering such a CCM into C3 plants (Hennacy and Jonikas 2020; Adler et al. 2022).

An important yet little-understood aspect of the Chlamydomonas CCM is the function and biogenesis of the thylakoid tubule network that traverses the pyrenoid, known as the pyrenoid tubules. The pyrenoid tubules are continuous with the thylakoid membrane (which harbors the photosynthetic electron transport chain) (Engel et al. 2015) and are thought to function as a delivery system for inorganic carbon (Ci) to the Rubisco-rich pyrenoid matrix (Raven 1997; Mitra et al. 2005). In the current model, bicarbonate (HCO_3_^−^) is channeled into the thylakoid lumen by bestrophin-like proteins 1-3 (BST1-3) (Mukherjee et al. 2019) and diffuses to the pyrenoid tubules where it is converted to CO_2_ by carbonic anhydrase 3 (Karlsson et al. 1998; Mitra et al. 2005) thanks to a low lumenal pH generated by the photosynthetic electron transport chain (Burlacot et al. 2022). Traversion of the pyrenoid Rubisco matrix by tubules is predicted to be essential for an efficient CCM (Fei et al. 2022). Therefore, understanding the mechanisms of pyrenoid tubule formation and function will be crucial for future plant pyrenoid engineering efforts.

**Figure 1.**
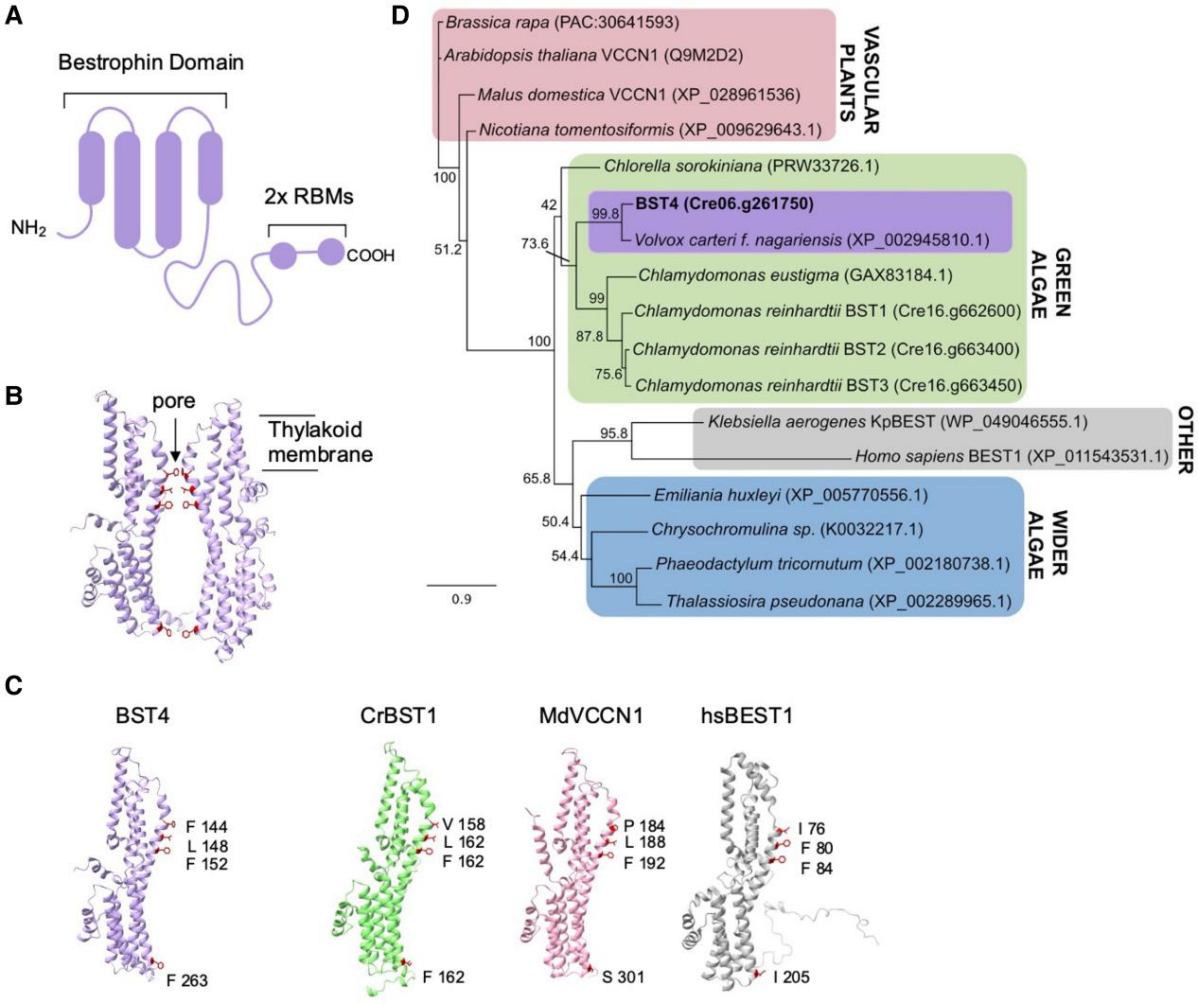
BST4 is a bestrophin protein that is distinct from BST1-3. **A)** Schematic of the topology of BST4. BST4 is predicted to have 4 transmembrane domains and a disordered C-terminus containing 2 RBMs. **B)** AlphaFold v2 models of two BST4 bestrophin domains (amino acid residues 53 to 386 shown for clarity) to show a typical bestrophin channel pore. Pore lining residue are side chains are shown. **C)** AlphaFold v2 structure of the BST4 bestrophin domains alongside the predicted structure of another bestrophin-like protein from *Chlamydomonas reinhardtii* (*Cr*BST1; Alphafold amino acids 51-end shown for clarity), and experimentally determined structures of bestrophins *Md*VCCN1 (7EK1) and *Hs*BEST1 (8D1I). Residues known to line the channel pore are highlighted. **D)** Phylogenetic analysis of the BST4 bestrophin domain (bold) with the disordered C-terminal removed. The alignment used was trimmed at residue 369. The evolutionary history of BST4 was inferred by using the maximum likelihood method based on the Le and Gascuel substitution model with discrete Gamma distribution (5 categories) and 500 bootstrap replicates. The tree is drawn to scale, with branch lengths measured in the number of substitutions per site.

The protein BST4 (bestrophin-like protein 4, also known as Rubisco binding membrane protein 1, RBMP1; Cre06.g261750) localizes exclusively to the pyrenoid tubules and has been proposed to function as a tethering protein, linking the Rubisco matrix to the tubules (Meyer et al. 2020). BST4 is a predicted transmembrane protein and has 2 Rubisco binding motifs (RBMs) on its long, disordered C-terminus (Fig. 1A) (He et al. 2020; Meyer et al. 2020). RBMs facilitate the targeting of proteins to the pyrenoid and are also hypothesized to underpin the assembly of the pyrenoid. Meyer et al. (2020) proposed that BST4, together with other tether proteins, may recruit Rubisco to the tubule network.

BST4 also has a well-conserved bestrophin domain similar to those in the thylakoid-localized BST1-3 proteins (Mukherjee et al. 2019). Bestrophins primarily act as anion channels and are found in a wide diversity of organisms, including animals, plants, and fungi. They are best known to have permeability to chloride and HCO_3_^−^ (Qu and Hartzell 2008), although some are reportedly permeable to cations (Yang et al. 2014) and larger organic anions (Roberts et al. 2011). BST4 may function as an HCO_3_^−^ channel, like that proposed for BST1-3 (Mukherjee et al. 2019), but there are currently no data to support this hypothesis.

In this study, we aimed to elucidate the role of BST4 in the Chlamydomonas pyrenoid. We tested BST4 as a thylakoid-Rubisco tethering protein as well as its suitability in promoting a thylakoid-Rubisco matrix interface in the model land plant Arabidopsis (*Arabidopsis thaliana*). By studying Chlamydomonas mutants impaired in the accumulation of BST4, we show that BST4 is critical for growth under fast light fluctuations likely thanks to its capacity to regulate lumenal proton concentration.

## Results and discussion

### BST4 is a bestrophin-like protein that is localized in the intra-pyrenoid thylakoid tubules in Chlamydomonas

The amino acid sequence of BST4 has 2 key unique features compared to the previously characterized BST1-3 (Fig. 1) (Mukherjee et al. 2019). First, BST4 has an extended disordered C-terminus that contains 2 RBMs (Fig. 1A) (He et al. 2020; Meyer et al. 2020). Second, BST4 has a phenylalanine residue in the first position of the putative selection pore, as opposed to valine which is conserved throughout BST1-3 (Mukherjee et al. 2019), although the residue at this position is variable in other well-characterized bestrophin proteins (*Malus domestica* voltage-dependent Cl^−^ channel 1 (*Md*VCCN1) and *Homo sapiens Bestrophin 1* (*Hs*BEST1)) (Fig. 1, B and C). In corroboration, we found the evolutionary history of BST4 diverges from BST1-3 when investigated using maximum likelihood phylogenetic analysis. We analyzed the full-length sequence (Supplementary Fig. S1) and a truncated version without the disordered C-terminal, leaving the bestrophin domain and N-terminal (Fig. 1D), both of which found BST4 to resolve in a distinct clade from BST1-3 but within the wider green algae group.

**Figure 2.**
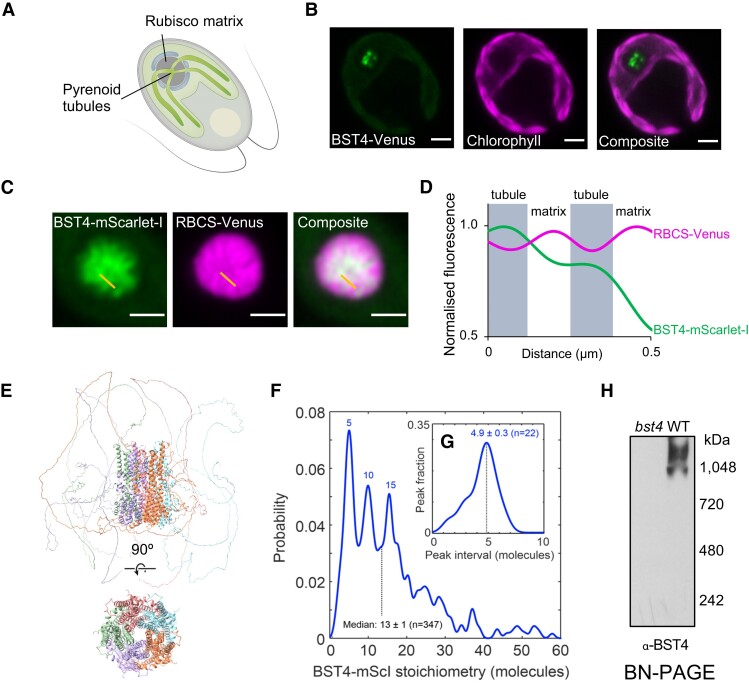
BST4 forms higher-order assemblies in the pyrenoid tubules in Chlamydomonas. **A)** Diagram of a Chlamydomonas cell with the pyrenoid Rubisco matrix and pyrenoid tubules indicated. **B)** Confocal image of a Chlamydomonas cell expressing BST4-Venus. Scale bar is 2 *µ*m. **C)** Pyrenoid in dual-tagged Chlamydomonas. BST4-mScarlet-I and RBCS-Venus are shown in green and magenta, respectively. Overlap appears white. The yellow line shows the 1D cross-section used for generating the line plot in (**D**). Scale bar is 1 *µ*m. **D)** Plot of normalized fluorescence intensity values from a 1D cross-section from (**C**). mScarlet-I and RBCS-Venus are shown in green and magenta, respectively. **E)** AlphaFold Multimer v2 prediction of pentameric BST4 structure. Top structure includes the disordered C-terminus, bottom structure displays amino acid residues 53 to 386 only for clarity. **F)** BST4-mScarlet-I stoichiometry probability distribution based on single particle tracking and molecular counting in live Chlamydomonas (*n* = 347 tracks) using Slimfield microscopy. **G)** Averaging the intervals between peaks in BST4-mScarlet-I stoichiometry (*n* = 22 intervals) indicates a consistent pentameric unit. **H)** Immunoblot of proteins from *bst4* mutant and WT Chlamydomonas thylakoids separated by Blue Native-polyacrylamide gel electrophoresis (BN-PAGE).

As well as being distinct at a sequence level, BST4 also localizes differently from BST1-3 (Fig. 2). While BST1-3 localizes throughout the thylakoid membrane and is enriched at the pyrenoid periphery (Mukherjee et al. 2019), BST4 localizes to the center of the pyrenoid in a pattern that resembles the pyrenoid thylakoid tubule system (Fig. 2, A and B; Meyer et al. 2020). To confirm tubule localization, we generated a dual-tagged line that expressed BST4-mScarlet-I and the Chlamydomonas Rubisco small subunit 1 (*Cr*RBCS1) fused to Venus. BST4-mScarlet-I was enriched where *Cr*RBCS1-Venus was depleted (Fig. 2, C and D), indicating that BST4 is located in the tubules and not the Rubisco-enriched pyrenoid matrix. Previous work suggests that the C-terminal RBMs of BST4 enable the protein to interact with *Cr*RBCS1 (Meyer et al. 2020). We confirmed using a yeast-2-hybrid approach that the C-terminus of BST4 interacts with *Cr*RBCS1 (Supplementary Fig. S2A). We also measured the efficiency of Förster resonance energy transfer (FRET) from Venus to mScarlet-I and found that the FRET efficiency was ∼35%, supporting the proximity of BST4 to *Cr*RBCS1 (Supplementary Fig. S2B).

Bestrophins typically form pentameric assemblies (Bharill et al. 2014; Hagino et al. 2022). When 5 chains of BST4 were inputted to AlphaFold a typical bestrophin pentameric structure was predicted (Fig. 2E). To test the complex assembly of BST4 in vivo, we utilized a Slimfield microscopy molecular tracking method (Plank et al. 2009). For this method, it is important to have only fluorescently tagged BST4 molecules and no native (untagged) BST4 molecules. To find a strain that was impaired in the accumulation of BST4 protein, we screened *bst4* insertional mutants from the CLiP library (Zhang et al. 2014; Li et al. 2019) and confirmed a mutant strain (*bst4*) (Supplementary Fig. S3 and methods). We then expressed BST4-mScarlet-I in the *bst4* mutant background (Supplementary Fig. S3). We used Slimfield microscopy to image BST4 in the pyrenoid tubules and subsequent image analysis (detailed in methods) to track individual fluorescent molecules of BST4-mScarlet-I and quantified the number of BST4 monomers per complex. The resulting probability distribution revealed that the most common BST4 complex is made up of 5 molecules (Fig. 2F). Other peaks showed complexes with numbers of molecules divisible by 5, which may be multiple pentameric channels grouping together. The interval between the probability peaks was also 5 (Fig. 2G). To further support higher-order complex assembly of BST4, we subjected purified Chlamydomonas thylakoid membranes to Blue Native-polyacrylamide gel electrophoresis (BN-PAGE) and immunodetected BST4 (Fig. 2H). BST4 formed a smear at ∼1,000 kDa, which is considerably larger than a pentamer (∼330 kDa). This could be due to higher-order assemblies of BST4, pentameric BST4 in a complex with other proteins and/or aberrant migration during BN-PAGE due to influences by complex shape. Collectively, our data support in vivo higher-order assembly of BST4 potentially as a pentamer.

### BST4 localizes to the stroma lamellae thylakoid membrane in Arabidopsis

We used Arabidopsis as a heterologous system to examine if BST4 was able to tether the Rubisco matrix to thylakoid membranes. In previous work, an Arabidopsis Rubisco small subunit (*At*RBCS) double mutant (*1a3b*) was complemented with *Cr*RBCS2, resulting in a line with hybrid Rubisco representing ∼50% of the Rubisco pool (S2_Cr_) (Izumi et al. 2012; Atkinson et al. 2017). Subsequent expression of the pyrenoid linker protein Essential PYrenoid Component 1 (EPYC1), in S2_Cr_ resulted in the formation of an EPYC1-hybrid Rubisco condensate or “proto-pyrenoid” (Atkinson et al. 2020). The S2_Cr_ line was therefore used as a platform to test whether BST4 acts as a tether protein.

**Figure 3.**
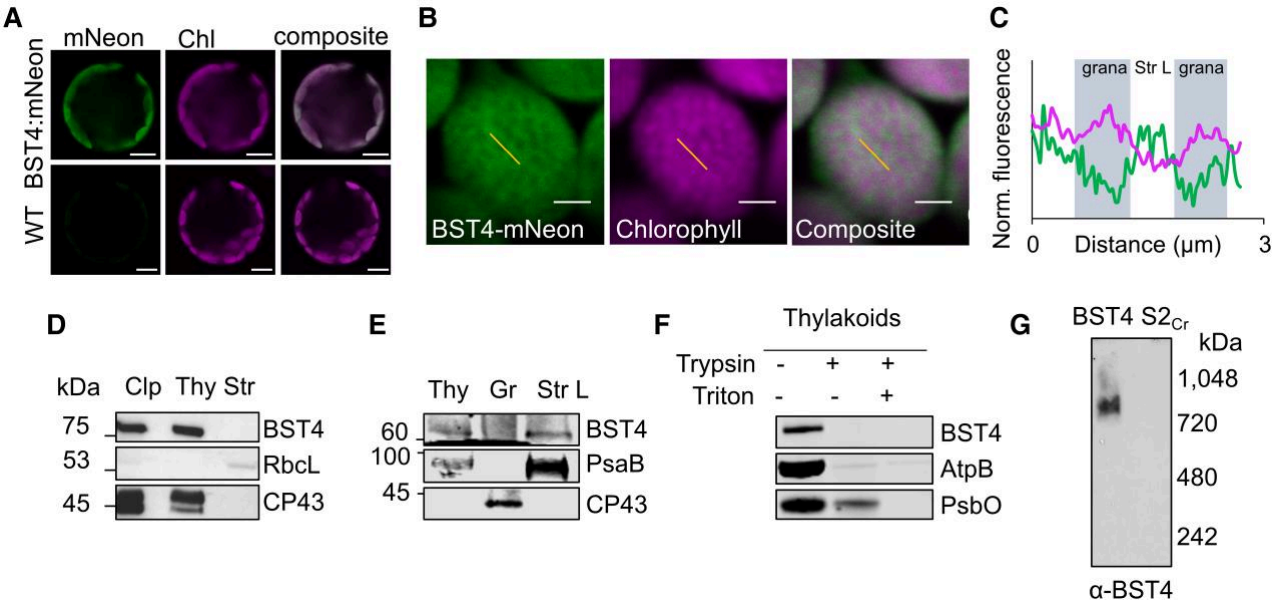
BST4 assembles as a complex in the stroma lamellae of thylakoids in Arabidopsis. **A)** Confocal image of WT protoplasts expressing BST4-mNeon. Scale bar is 10 *µ*m. **B)** Mesophyll chloroplast from S2_Cr_ Arabidopsis expressing BST4-mNeon. BST4-mNeon and chlorophyll autofluorescence are shown in green and magenta, respectively. Overlap appears white. Scale bar is 2 *µ*m. Yellow lines indicate selections for profile plot in **(C)**. **C)** Plot of normalized fluorescence intensity values from a 1D cross-section (yellow line) through 2 grana stacks from **(B)**. mNeon and chlorophyll autofluorescence are shown in green and magenta, respectively. **D)** Immunoblots of sub-chloroplast fractions isolated from Arabidopsis line S2_Cr_ expressing BST4. RbcL and CP43 were probed for as stromal and thylakoid controls, respectively. **E)** Immunoblots of fractionation thylakoids from Arabidopsis line S2_Cr_ expressing BST4. CP43 and PsaB were used for grana stack and stroma lamellae controls, respectively. **F)** Trypsin protease protection assay. Intact thylakoids containing BST4 subjected to 0 or 100 *μ*g/mL trypsin with or without the addition of 1% (v/v) Triton. AtpB and PsbO used as controls for stromal facing (exposed) and lumen facing (protected), respectively. **G)** Immunoblot of proteins from thylakoids separated by Blue Native-PAGE from either BST4 stable line or S2_Cr_ background. Abbreviations: Clp, whole chloroplast; CP43, CP43-like chlorophyll binding protein; RbcL, Rubisco large subunit; PsbO, photosystem II manganese-stabilizing polypeptide; AtpB, Adeonsine TriPhosphate synthase subunit beta; PsaB, photosystem I P700 chlorophyll a apoprotein A2; Str, stromal fraction; Thy, thylakoid fraction.

We initially expressed a BST4-mNeon fusion protein in S2_Cr_ (without EPYC1) to first confirm the correct localization of BST4 to thylakoids without a Rubisco condensate present (Fig. 3). BST4-mNeon was observed in chloroplasts, demonstrating that the native chloroplast signal peptide was compatible with the chloroplast targeting mechanism in land plants (Fig. 3A), as seen previously for other Chlamydomonas proteins (Atkinson et al. 2016). The fluorescence signal from BST4-mNeon had a sponge-like pattern that inversely corresponded with the punctate chlorophyll autofluorescence signal that represents the grana stacks (Fig. 3, B and C). The sponge-like pattern was similar to previous observations of autofluorescence originating from photosystem (PS) I (Hasegawa et al. 2010), which is enriched in the stroma lamellae of thylakoids. We subsequently generated a stable Arabidopsis transgenic line expressing untagged BST4 to confirm its location by biochemical fractionation. BST4 was detected in the thylakoid fraction and not in the stromal fraction (Fig. 3D). The thylakoids were then further fractionated into grana stacks and stroma lamellae sub-fractions. BST4 was found in the stroma lamellae fraction (Fig. 3E), which is consistent with the observed sponge-like fluorescence pattern.

As BST4 has 2 RBMs on its C-terminus, we hypothesized that BST4 should be orientated with the C-terminus facing the stroma so that the RBMs are available to interact with *Cr*RBCS. A topology prediction of BST4 also predicts that BST4 is likely oriented with the C-terminus facing the stroma due to the C-terminal side having a greater frequency of positively charged residues (Supplementary Fig. S4) (Gavel et al. 1991).To determine the orientation of BST4, we performed a protease protection assay on thylakoids isolated from the untagged BST4 transgenic plants. Our antibody was raised against the C-terminal end of BST4, so it could be used to assess whether the C-terminus was exposed to degradation in the stroma or if it was protected in the lumen. We found that the BST4 C-terminus was fully degraded after a 60 min treatment of trypsin, indicating that it faced the stroma (Fig. 3F). There was some degradation of the lumenal control, the PSII subunit PsbO, which we attribute to a portion of the thylakoid membrane preparation not being fully intact. However, PsbO was fully degraded when the membranes were solubilized, indicating that they were sufficiently intact to differentiate between lumenal- and stromal-facing peptides. Therefore, BST4 was observed in the expected location and orientation in plant thylakoid membranes.

Finally, we also tested whether BST4 forms a complex in the Arabidopsis thylakoid membrane. We subjected thylakoids from the untagged BST4 transgenic plants to BN-PAGE and detected a single band of ∼850 kDa (Fig. 3G). Thus, BST4 forms a similar high-order complex in Arabidopsis to that in Chlamydomonas but may be lacking additional interaction partners present in Chlamydomonas.

### BST4 is not sufficient for the integration of thylakoids into the Arabidopsis proto-pyrenoid

**Figure 4.**
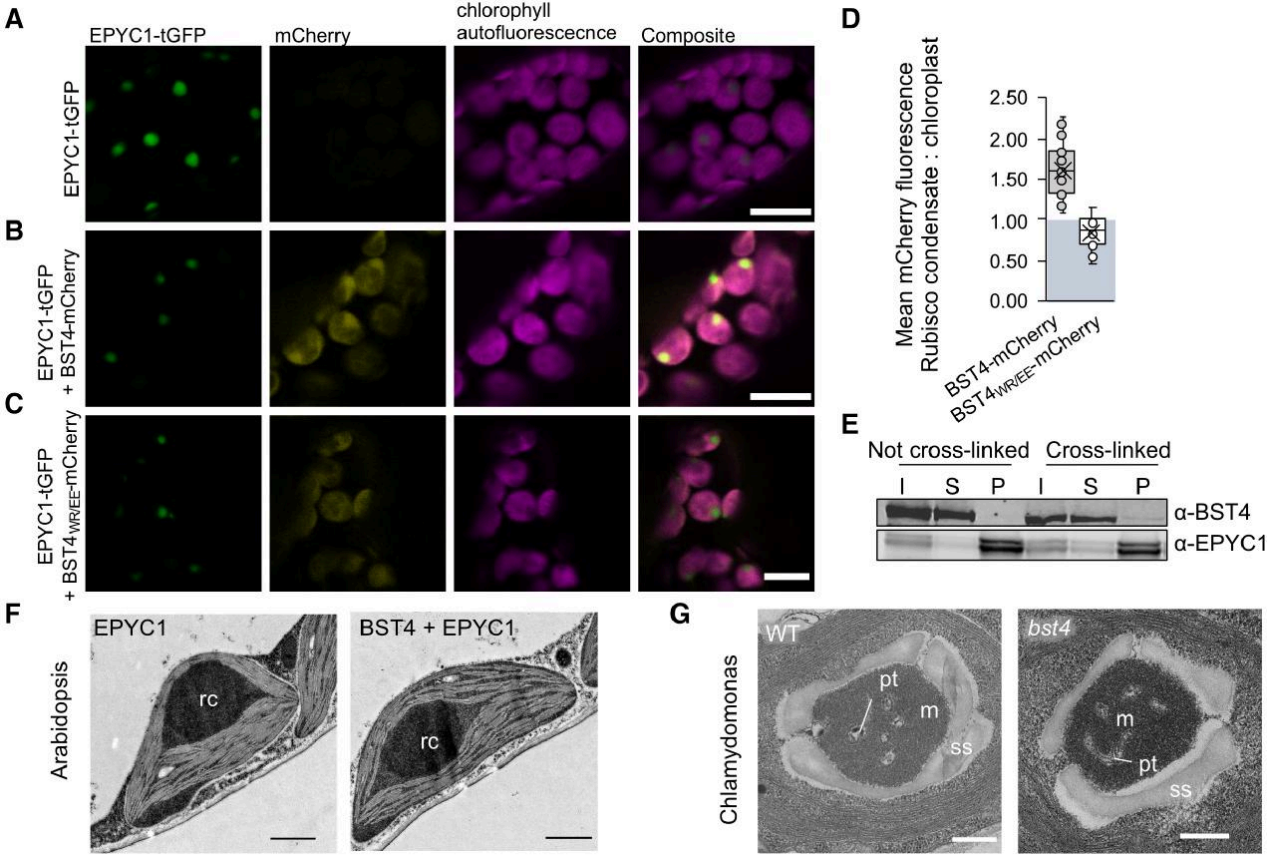
BST4 is not sufficient to enable the inclusion of thylakoid membranes in a rubisco condensate in Arabidopsis and is not required for pyrenoid tubule inclusion in the Chlamydomonas pyrenoid. **A)** Confocal images showing EPYC1-tGFP in S2_Cr_ Arabidopsis background. Scale bar is 10 *µ*m. **B)** Confocal images showing BST4-mCherry co-expressed with EPYC1-tGFP in S2_Cr_ Arabidopsis background. Scale bar is 10 *µ*m. **C)** Confocal images showing BST4 with mutated RBMs (BST4_(WR/EE)_) fused to mCherry co-expressed with EPYC1-tGFP in the S2_Cr_ Arabidopsis background. **D)** Box plot of the ratio of mean mCherry fluorescence associated with the Rubisco condensate compares do the rest of chloroplast when mCherry was fused to either BST4 or BST4_(WR/EE)_. Box plot elements are as follows: centerline, median; box, interquartile interval; whiskers, upper and lower quartiles; X mark, mean; circles, data points (*n* = 22–23). **E)** Immunoblot of sedimented Rubisco condensates from S2_Cr_ Arabidopsis expressing EPYC1-tGFP and BST4-mCherry. Abbreviations: I, input; S, supernatant; P, pellet. Rubisco condensates are enriched in the pelleted fraction. Cross-linked samples were prepared by vacuum infiltrating intact leaves with 1% (v/v) formaldehyde prior to sedimentation. **F)** Transmission electron micrograph of a chloroplast from S2_Cr_ Arabidopsis expressing either EPYC1 alone or EPYC1-tGFP and BST4-mCherry. Rc is the Rubisco condensate. Scale bar is 1 *µ*m. **G)** Transmission electron micrograph of pyrenoid from WT Chlamydomonas or *bst4* mutant. Abbreviations: pt, pyrenoid tubules; m, matrix; s, starch sheath. Scale bar is 250 nm.

To test whether BST4 could facilitate incorporation of thylakoids into hybrid Rubisco condensates (i.e. proto-pyrenoids) in plants, BST4-mCherry and EPYC1-tGFP were co-expressed in the S2_Cr_ background (Fig. 4). When we expressed EPYC1 alone, Rubisco condensates formed in chloroplasts as previously described by Atkinson et al. (2020), and were visible as a ∼2 *µ*m wide puncta in the tGFP channel (Fig. 4A). When BST4-mCherry and EPYC1-tGFP were co-expressed, approximately 60% of the BST4-mCherry fluorescence signal was observed in the condensate region (Fig. 4, B and D), with the remaining signal exhibiting the same sponge-like pattern as shown in Fig. 3B.

To confirm that the observed co-localization was due to an interaction between BST4 and *Cr*RBCS2, we mutated the first 2 residues of each core RBM motif of BST4 to glutamic acid (Tryptophan and Arginie to Glutamate and Glutamate, WR to EE, the new version noted BST4_WR/EE_). Previously, these substitutions have been reported to disrupt the binding interface between EPYC1 and *Cr*RBCS2 (He et al. 2020). Using yeast-2-hybrid, we confirmed that the interaction between the C-terminus of BST4 and *Cr*RBCS2 was disrupted by these mutations (Supplementary Fig. S2). When BST4_WR/EE_-mCherry was expressed with EPYC1-tGFP in S2_Cr_, no enrichment of the mCherry signal was observed in the Rubisco condensate (Fig. 4, C and D). Thus, the RBMs of BST4 were responsible for the enrichment of BST4 in the vicinity of the Rubisco condensate in Arabidopsis.

However, when condensates were sedimented and analyzed by immunoblotting, BST4-mCherry was not detected in the condensate fraction (Fig. 4E). When leaf samples were subjected to formaldehyde cross-linking prior to sedimentation of the condensate, a small amount of BST4 was present in the condensate fraction. We concluded that BST4 was not present in the condensate itself but preferentially occupied the thylakoid membranes surrounding the *Cr*RBCS2-enriched condensate (Atkinson et al. 2020), likely due to interactions with *Cr*RBCS2.

Although BST4 partially co-localized with the Rubisco condensate, we found no evidence to suggest that BST4 could facilitate the inclusion of thylakoid membranes. Confocal microscopy was not sufficient to determine if chlorophyll autofluorescence was in the proto-pyrenoids of S2_Cr_ lines expressing BST4-mCherry and EPYC1-tGFP. However, transmission electron microscopy (TEM) revealed no visible indication of thylakoid membranes in the condensates, which were structurally similar to condensates in S2_Cr_-EPYC1 lines lacking BST4 (Fig. 4F). Thus, BST4 appeared insufficient to enable thylakoid inclusion in the Rubisco condensate in Arabidopsis.

### BST4 has no impact on growth and photosynthesis in the Arabidopsis S2_Cr_ line

To test whether BST4 has any influence on plant thylakoid membranes, we generated 3 independent BST4 no tag lines in the S2_Cr_ background and used them to assess the impact of BST4 on Arabidopsis physiology (Supplementary Fig. S5A). We found there was no difference in growth between plants expressing BST4 and their azygous segregants, as determined by the rosette area (Supplementary Fig. S5B). We also found that BST4-expressing plants tended to have slightly lower *F_v_*/*F_m_* (maximum quantum yield of PSII) as compared to azygous segregants and the parent line, although this was not significant (Supplementary Fig. S6A). Further measurements were made for lines 2 and 3 on the kinetics of non-photochemical quenching (NPQ), Y(II), and for proton motif force (PMF) size and partitioning but no consistent differences were observed (Supplementary Fig. S6).

### BST4 is not necessary for tubule formation in Chlamydomonas

To investigate whether BST4 was necessary for the normal formation of the thylakoid tubule structure in Chlamydomonas we compared the structure of pyrenoids of *bst4* compared to the wild type (WT) control strain (CMJ030) (Zhang et al. 2014; Li et al. 2019). TEM images showed that pyrenoids from *bst4* were structurally comparable to the WT control, including the presence of pyrenoid tubules (Fig. 4G). There were also no differences in pyrenoid size or shape between the 2 lines when comparing pyrenoids from 40 to 50 cells from each genotype (Supplementary Fig. S7). As a result, we conclude that BST4 is not necessary for the pyrenoid tubule–Rubisco matrix interface in Chlamydomonas.

### The C-terminus of BST4 is required for localization to the pyrenoid tubules

**Figure 5.**
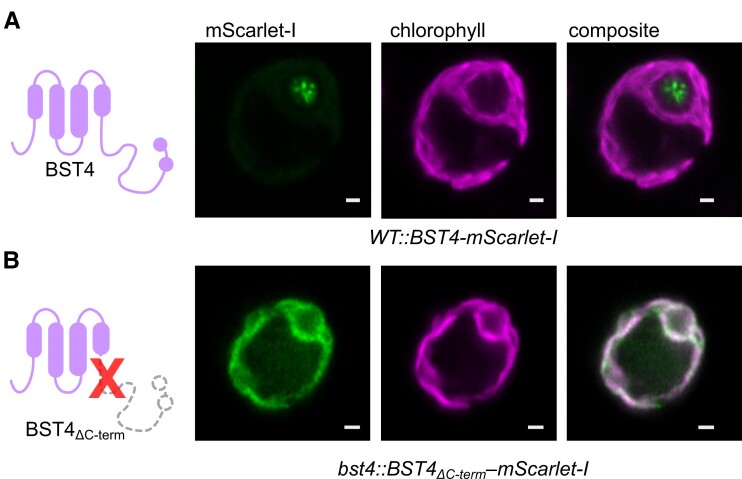
C-terminus of BST4 is required for pyrenoid localization. **A)** Confocal image of full-length BST4-mScarlet-I in WT. Diagram on the left-hand side depicts full-length BST4. **B)** Confocal image of BST4_ΔC-term_-mScarlet-I in *bst4*. Diagram on the left-hand side depicts BST4 with C-terminus truncated. Scale bars are 1 *µ*m.

Multiple copies of the RBM are sufficient to target proteins to the pyrenoid (Meyer et al. 2020). Furthermore, we have shown that the BST4 RBMs are required for RBCS interaction via yeast-2-hybrid (Supplementary Fig. S2A) and that they are required for enrichment with the proto-pyrenoid in Arabidopsis (Fig. 4, B to D). To investigate the role of the RBMs in BST4 localization in Chlamydomonas, we generated a truncated version of BST4 (residues 1 to 386) that lacked the C-terminus containing the 2 RBMs (BST4_ΔC-term_) and compared localization with full-length BST4 expressed in WT and the *bst4* mutant (Fig. 5 and Supplementary Fig. S8). BST4_ΔC-term_-mScarlet expressed in the *bst4* mutant line did not localize to the pyrenoid tubules but was found throughout the thylakoid membrane (Fig. 5). This is consistent with our findings in Arabidopsis with BST4_WR/EE_ (Fig. 4C), demonstrating that localization of BST4 is driven via RBM-Rubisco interaction. Thus, the C-terminus is necessary for BST4 localization to the pyrenoid tubules in the presence of the matrix. Collectively, these data indicate that BST4 may require a preexisting pyrenoid tubule network to be localized in the pyrenoid rather than driving the inclusion of thylakoid membranes into the Rubisco matrix or is redundant as a tether protein.

When BST4_ΔC-term_-Venus was expressed in WT Chlamydomonas (i.e. still expressing the native version of BST4), we observed fluorescence throughout the thylakoids, but with the majority of the signal still localized to the pyrenoid tubules (Supplementary Fig. S8). This is in contrast to BST4_ΔC-term_-mScarlet expressed in the *bst4* mutant where, presumably, only homomeric truncated pentamers are formed and all the signal was localized throughout the chloroplast. We conclude that BST4_ΔC-term_-Venus is recruited to the pyrenoid through heteromeric pentamers constituting truncated and native full-length BST4, which is further evidence that BST4 oligomerizes.

We next investigated whether BST4 localizes to the pyrenoid tubules alone, or whether BST4 localizes to the tubules through an interaction with Rubisco. To do so, we utilized a Chlamydomonas mutant generated by Genkov et al. (2010) that expresses *At*RBCS but lacks both isoforms of *Cr*RBCS (*Crrbcs::AtRBCS*) and thus lacks a Rubisco matrix as EPYC1 does not interact with *At*RBCS (Atkinson et al. 2019). *Crrbcs::AtRBCS* retains reticulated thylakoid membranes, unlike the laminar arrangement of stromal thylakoids, at the canonical pyrenoid site, which are likely the nascent pyrenoid tubule network (Caspari et al. 2017) (Supplementary Fig. S9A). Caspari et al. (2017) show large starch granules accumulating at the canonical pyrenoid site, which we confirmed by expressing STA2-Venus as a starch marker (Mackinder et al. 2017) in *Crrbcs::AtRBCS* (Supplementary Fig. S9B). We expressed BST4-Venus in *Crrbcs::AtRBCS* and found that BST4 localized to a punctum adjacent to the canonical pyrenoid site, which we attribute to the nascent pyrenoid tubules (Supplementary Fig. S9C). To confirm this, we also expressed a known pyrenoid tubule marker protein, PsaF (Emrich-Mills et al. 2021) in *Crrbcs::AtRBCS*, which showed a similar localization pattern to that of BST4 (Supplementary Fig. S9D). This suggests there may be an additional Rubisco binding independent mechanism for the localization of BST4 as well as other proteins to the pyrenoid tubules. Further investigation of the role of the whole C-terminal domain will be required to understand the mode of BST4 pyrenoid tubule localization.

### Chlamydomonas *bst4* mutant is not defective in growth under continuous high light but has increased H_2_O_2_ production

**Figure 6.**
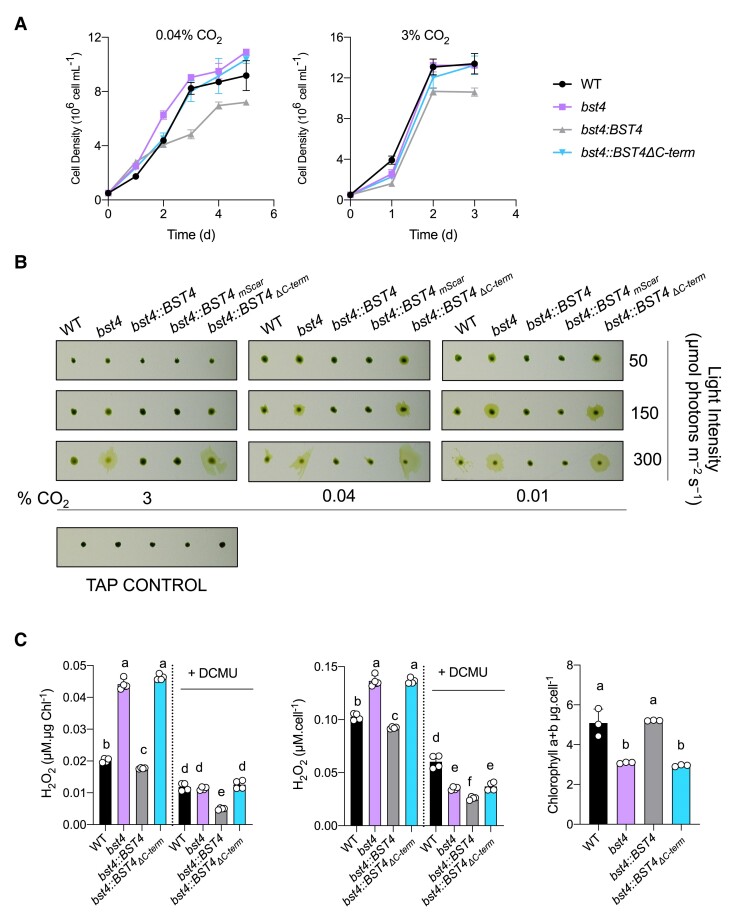
The *bst4* mutant does not have an impaired growth phenotype under CCM induced conditions but has increased H_2_O_2_ production. **A)***Chlamydomonas* strains were subjected to a liquid growth assay using pH 7.4 TP minimal media that was bubbled with 0.04% CO_2_ or 3% CO_2_ (+/− 2 ppm). Error bars are ± SEM (*n* = 3). **B)** Dot assay of WT, *bst4,* complemented *bst4::BST4* and *bst4* complemented with C-terminal truncation of BST4 (*bst4::BST4_Δ_*_C-term_) on minimal pH 7.4 TP agar at indicated light intensities and CO_2_ concentrations. **C)** H_2_O_2_ assay. Chlamydomonas cells were grown in pH 7.4 TP liquid media and exposed to 150 *µ*mol photons m^−2^s^−1^ for 24 h with or without the photosynthetic inhibitor DCMU (10 *µ*M). The concentration of H_2_O_2_ was subsequently quantified using Amplex Red (*n* = 4), and is presented both proportionately to cell density and chlorophyll content. Chlorophyll content was quantified for all cell lines (*n* = 3). Error bars are ± SEM. Different letters indicate significance (*P* < 0.05) as determined by a one-way ANOVA and Tukey's post-hoc test.

To test whether BST4 has a role in the operation of the CCM, we measured the growth of *bst4* compared to the WT control strain under various CO_2_ conditions (Fig. 6 and Supplementary Fig. S10). Spot assays did not reveal any reduction in growth under CO_2_-limiting conditions (Supplementary Fig. S10). When grown in liquid medium, *bst4* even seemed to grow slightly better than WT when sparged with 0.04% CO_2_ (Fig. 6A). However, when comparing the calculated specific growth rates (*μ* h^−1^) for both the exponential growth phase (days 0 to 3, *bst4* 0.0402 ± 0.0003 *μ* h^−1^ and WT 0.0389 ± 0.0007 *μ* h^−1^) or the full growth assay (days 0 to 5, *bst4* 0.0257 ± 0.0001 *μ* h^−1^ and WT 0.0241 ± 0.001 *μ* h^−1^), there was no statistically significant increase in specific growth rates between *bst4* and WT (two-tailed *t*-test, *P* = 0.17 and 0.20, respectively, *n* = 3, full results Supplementary Table S1). We conclude from these experiments that BST4 is not essential for growth at air levels of CO_2_ and might not be necessary for the functioning of the CCM. We also included the complemented *bst4::BST4* and *bst4::BST4_ΔC-term_-mScarlet-I* (hereafter *bst4::BST4_ΔC-term_*) lines in the spot and liquid growth assays. While all lines grew well in the spot assay, in the liquid growth *bst4::BST4_ΔC-term_* grew comparably to WT and *bst4* whereas *bst4::BST4* exhibited a slightly reduced growth than the other lines in both CO_2_ conditions.

One noticeable difference between *bst4* and WT lines from the growth assays conducted on solid media (Fig. 6B and Supplementary Fig. S10) was that *bst4* cells had a distinct halo of diffuse cells on the periphery of the colony. We used a range of CO_2_ and light conditions to investigate the diffuse colony phenotype (Fig. 6B) and found it was most apparent under high light (300 *μ*mol photons m^−2^ s^−1^) and low or very low CO_2_ conditions (0.04% CO_2_, 0.01% CO_2_, respectively) (Fig. 6B). WT, *bst4::BST4* and *b*s*t4::BST4-mScarlet-I* complemented lines had little or no diffusivity, although WT did display a slightly diffuse colony phenotype at 300 *μ*mol photons m^−2^ s^−1^. Interestingly, *b*s*t4::*BST4_ΔC-term_ was unable to rescue the diffuse colony phenotype suggesting that either the presence of the C-terminus or localization to the tubules is essential for the function of BST4.

The diffuse colony phenotype was most apparent under high light (300 *μ*mol photons m^−2^ s^−1^), to a slightly lesser extent at medium light (150 *μ*mol photons m^−2^ s^−1^), and exacerbated by low CO_2_ (0.01% and 0.04%). These are conditions where carbon fixation can be limiting and therefore, the energy production by photosynthesis could exceed the energetic demand required to fix CO_2_ by the CCM and the Calvin cycle. This imbalance can result in the release of reactive oxygen species (ROS) (Erickson et al. 2015). Because cells exposed to ROS have altered phototactic responses to light (Wakabayashi et al. 2011), we thus thought to assess the phototactic capacity of *bst4* and its control WT strain by exposing them to directional light in liquid culture (Supplementary Fig. S11). In this assay, *bst4* cells displayed strong positive phototaxis, whereas WT and complemented *bst4::BST4* lines displayed negative phototaxis. To test whether the phototactic response of *bst4* was due to an increase in ROS production, we recorded the direction of phototaxis in cells exposed to either ROS or a ROS quencher. In the presence of the ROS quencher N, N'-dimethylthiourea (DMTU), *bst4* positive phototaxis was disrupted, resulting in a negative response to directional light (Supplementary Fig. S11A). When the ROS H_2_O_2_ (75 *μ*M) was added, WT, *bst4::BST4*, and *bst4* displayed positive phototaxis (Supplementary Fig. S11A) indicating that the positive phototaxis observed in *bst4* is likely governed by increased ROS production.

To directly quantify the difference in ROS generation, we analyzed the H_2_O_2_ produced by cells exposed to 150 *μ*mol photons m^−2^ s^−1^ (Fig. 6C). The *bst4* and *bst4::BST4_ΔC-term_* lines had significantly higher H_2_O_2_ production than WT and *bst4::BST4* when normalized to both chlorophyll content and cell density. We validated the assay by using the same ROS quencher DMTU from Supplementary Fig. S11A and saw a consistent reduction in H_2_O_2_ detected in all lines (Supplementary Fig. S11B). Therefore, *bst4* has a higher H_2_O_2_ generation than its control strains.

To test the involvement of photosynthetic activity in the increased ROS production, we also treated cells with the PSII plastoquinone binding site inhibitor 3-(3,4-dichlorophenyl)-1,1-dimethylurea (DCMU) whereby the concentration of H_2_O_2_ produced was reduced in all lines (Fig. 6C). To assess possible explanations for photosynthetic ROS production, we probed the reduction state of the plastoquinone pool (PQ) pool, which is a possible source of H_2_O_2_ (Khorobrykh and Tyystjärvi 2018). We used the chlorophyll fluorescence parameter 1-qL, which represents the fraction of closed PSII centers and can be indicative of the donor side reduction state of the PSII (Kramer et al. 2004), and therefore PQ reduction level. We found that 1-qL was slightly lower in *bst4* compared to WT, which does not support a stronger ROS production originating from the PQ pool (Supplementary Figs. S12 and S13). Another possibility for the origin of the ROS is from the Mehler reaction (Asada 1999; Badger et al. 2000). We conclude that the origin of increased H_2_O_2_ in *bst4* is light-dependent but further work is needed to determine the origin of the increased ROS.

During high light exposure, Chlamydomonas typically undergoes transcriptional changes to protect the cell from excess light energy. Reduction in cellular chlorophyll and the increase in ROS scavenging pigments is a known physiological response to high light exposure and subsequent elevated ROS production (Bonente et al. 2012). We found that *bst4* and *bst4::BST4_ΔC-term_* had significantly lower cellular chlorophyll content than WT and *bst4::BST4.* Another important part of photoprotection depends on the safe dissipation of absorbed light energy into heat via NPQ. To assess the level of NPQ in response to high light exposure, we used a pulsed amplitude modulation fluorimeter (PAM) to measure chlorophyll fluorescence of cells adapted to 3 h of high light (150 *μ*mol photons m^−2^ s^−1^) (Supplementary Fig. S14). We used the PAM to assess the quantum yield of PSII (Y(II)) and the amount of NPQ. We found that *bst4* was able to maintain a higher *F*_v_*/F*_m_ than WT (Supplementary Fig. S14A), suggesting that the increased ROS is likely independent of photoinhibition. *Bst4 also* had higher sustained NPQ compared to WT (Supplementary Fig. S14B). The fact that *bst4* mutants grow comparably to WT, despite the increased NPQ and ROS could be due to a redistribution of electrons to alternative electron flows (Burlacot 2023). There were higher levels of light harvesting complex stress related 3 (LHCSR3) protein as compared to the WT strain (Supplementary Fig. S14, G and H). LHCSR3 mediates energy-dependent NPQ in response to thylakoid membrane acidification (Peers et al. 2009; Bonente et al. 2011; Tian et al. 2019; Steen et al. 2022). No significant differences in PMF or the proton conductivity of thylakoid membranes (g_H_^+^) were observed although the dissipation rate of the PMF (v_H_^+^) was higher in *bst4* compared to WT (Supplementary Fig. S14, D, E, I, and J). The higher v_H_^+^ (Supplementary Fig. S14F) together with the higher expression of LHCSR3 suggest a buildup of protons in the tubule lumen. A more acidic lumen might reduce the rates of the Mehler reaction, suggesting it may not be the primary source of H_2_O_2_ (Roach et al. 2015). Since ROS induces photoprotective mechanisms (Roach and Na 2017), we propose that, in the absence of BST4, enhanced production of ROS during prolonged high light exposure (Fig. 6) leads to enhanced photoprotective mechanisms such as increased LHCSR3 production and pigment changes.

### Chlamydomonas *bst4* mutant is impaired in acclimation to light fluctuations

**Figure 7.**
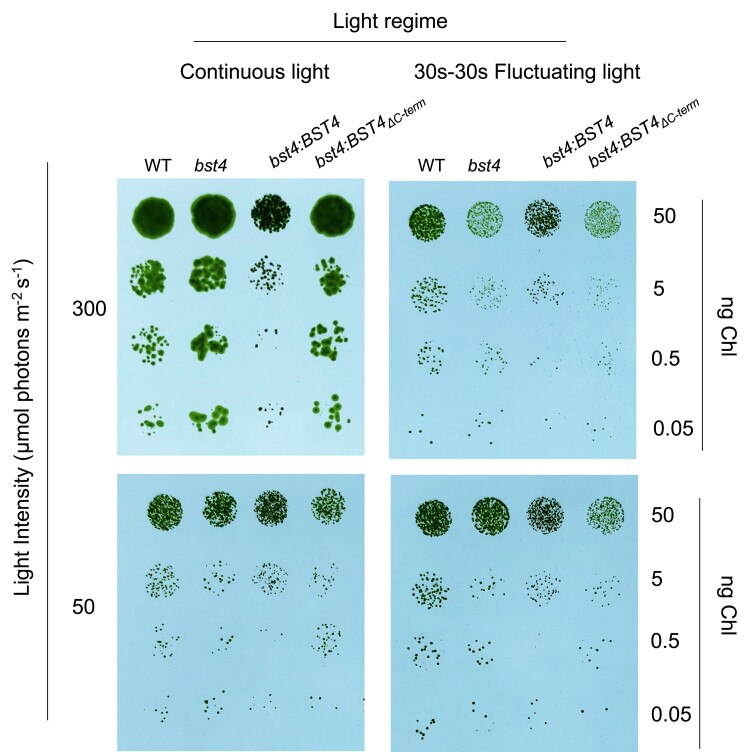
*Bst4* has a growth defect under fluctuating light. *Chlamydomonas* strains were grown in serial dilution on (modified high salt) HS agar plates under air CO_2_ levels and indicated light regimes. Images from continuous light and fluctuating light conditions were from day 6 and 13 of growth, respectively. The amount of cells per spot was quantified by chlorophyll as indicated. This spot test is representative of seven independent biological repeats.

Some photosynthetic mutants show little to no defect during continuous light, but are sensitive to light fluctuations (Chaux et al. 2017; Jokel et al. 2018; Eckardt et al. 2024). We therefore decided to test the growth of the strains under fluctuating light, since numerous thylakoid ion channels are active in the first minute of illumination (Armbruster et al. 2017; Schneider et al. 2019). We grew cells on solid minimal media under different light regimes. We found that under fluctuating high light, *bst4* had a growth defect compared to the WT and the line complemented with full-length BST4 (Fig. 7 and Supplementary Fig. S15). The growth defect of *bst4* was not rescued by high CO_2_ conditions, rather it was more apparent, suggesting that BST4 has a role independent of the presence of a CCM. *Bst4* complemented with BST4_ΔC-term_ grew comparably to *bst4* except under 2% CO_2_, where the growth was partially restored. The *bst4* CLiP mutant has two other verified insertion sites (Supplementary Fig. S3). Although complementation with BST4 reversed the growth phenotype under all conditions, we wanted to assess the impact of the genetic background on the *bst4* CLiP mutant phenotype. To this end, we generated two additional BST4 knockout mutants using CRISPR-Cas9 (*bst4-2; bst4-3*) (Supplementary Fig. S16). Both the *bst4-2* and *bst4-3* mutants had a fluctuating light growth defect under 300 or 600 *μ*mol photons m ^−2^ s^−1^ at air level and 2% CO_2_, but the phenotype was less reliable than the *bst4* phenotype (Supplementary Fig. S15). Therefore, the fluctuating light *bst4* phenotype is dependent on the genetic background used. We conclude that BST4 is involved in acclimation to fluctuating light, although there may be some exacerbating polygenic effects in the CLiP line that make the role of BST4 more prominent.

### BST4 regulates the lumenal pH in Chlamydomonas during the dark-to-light transition

**Figure 8.**
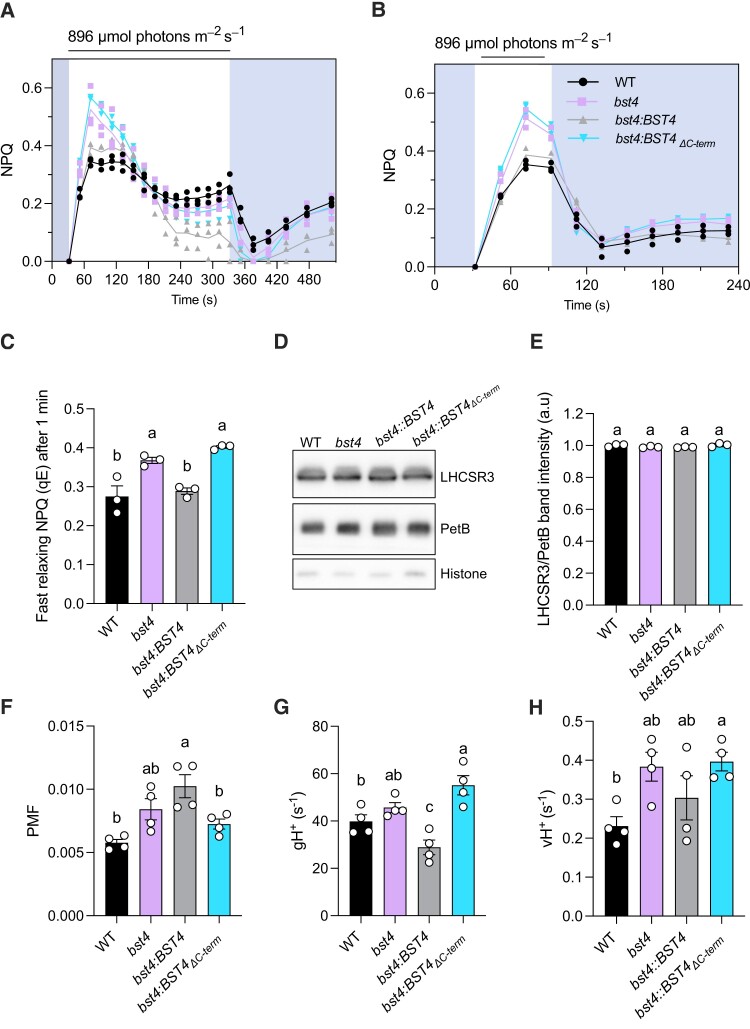
Chlamydomonas *bst4* mutant has an enhanced initial NPQ response. WT and mutants were grown in high salt (HS) medium at 80 *µ*mol photons m^−2^ s^−1^ and measured at 10 *µ*g Chl mL^−1^ (prepared by dilution). The cells were dark adapted for 5 min before the measurements. **A)** Dynamics of NPQ on transition from dark to high light. Kinetics for induction of chlorophyll fluorescence were recorded during 5 min of illumination at 896 *µ*mol photons m^−2^ s^−1^ followed by 5 min in darkness. Shown are individual data points (dots) and their average (lines) (*n* = 3). Shading on graphs indicates when sample is in the dark, unshaded regions indicate application of actinic light. **B)** Dynamics of NPQ during dark-to-light transition with 1 min of light exposure. Shown are individual data points (dots) and their average (lines) (*n* = 3). Shading on graphs indicates when sample is in the dark, unshaded regions indicate application of actinic light. **C)** Calculated fast relaxing NPQ after 1 min of light exposure as determined by NPQ at the light-to-dark transition minus the minimum NPQ in the dark. Bars show the individual data points and the mean ± SEM (*n* = 3). **D)** Immunoblot of NPQ protein LHCSR3 in each strain plus PetB (which encodes cytochrome *b*_6_) and Histone as loading controls. **E)** Quantification of LHCSR3 band intensity normalized to PetB. Each point is the mean of 2 technical replicates from one biological replicate. **F)** Total PMF as measured from ECS measurements. Shown are average of 3 technical replicates for each biological replicates (*n* = 4 biological replicates). **G)** Proton conductance (g_H_^+^) and **H)** proton flux (v_H_^+^) were determined after 1 min illumination at 890 *µ*mol photons m^−2^ s^−1^. (*n* = 4 biological replicates). Bars show the individual data points and the mean ± SEM. Different letters indicate significance (*P* < 0.05) as determined by a one-way ANOVA and Tukey's post-hoc test.

To understand the role of BST4 under fluctuating light, we measured the induction of NPQ during a dark-to-light transition. NPQ is mediated by multiple mechanisms and harbors multiple components (Erickson et al. 2015), one of them, termed energy-dependent quenching (qE), is quickly induced and relaxed and is mostly mediated by LCHSR3 protein. The magnitude of qE has recently been shown to be an indicator of the lumenal pH, the lower the pH, the higher the qE (Tian et al. 2019). While no differences in Y(II) were observed between the strains upon a dark-to-light transition (Supplementary Fig. S17), the NPQ of *bst4* mutant was transiently higher than WT (Fig. 8), the NPQ becoming indistinguishable from WT after 3 min of illumination (Fig. 8A). The complementing *bst4:BST4* strain was indistinguishable from the WT control during the first 3 min of illumination and *bst4:BST4_ΔC-term_* had a NPQ similar to *bst4* (Fig. 8A). To establish the nature of the transiently increased NPQ in *bst4*, we used a shorter illumination time (Fig. 8B). For both *bst4* mutant and WT, NPQ was quickly dissipated in the dark (Fig. 8, B and C). Similar trends of NPQ kinetics were also observed when cells were supplemented with HCO_3_^−^ before the measurement, although NPQ relaxation in the dark was different between lines (Supplementary Fig. S18). The initial increase in NPQ observed in *bst4* could therefore be attributed to qE. The transiently increased NPQ compared to WT was recapitulated with a smaller amplitude in *bst4-2* whereas *bst4*-3 had similar NPQ induction to WT (Supplementary Fig. S19). We hypothesize that the magnitude of NPQ induction is influenced by differences in the genetic background between *bst4* CLiP and CRISPR strains. There is also a possibility that *bst4*-3 still expresses levels of BST4 that we could not detect. We note here that the mechanism behind the role of BST4 in fluctuating light acclimation may not be solely due to NPQ induction and requires further investigation.

To test whether the initial increased NPQ in *bst4* was linked to a lower lumenal pH as compared to the WT, we added the ionophore nigericin when the NPQ difference between *bst4* and WT cells was maximal (Supplementary Fig. S20). In both *bst4* mutant and its control, nigericin quickly dissipated NPQ within 3 min after injection (Supplementary Fig. S20), suggesting that NPQ was lumenal pH dependent. Since *bst4* did not accumulate more LHCSR3 as compared to WT in these conditions (Fig. 8, D and E), we conclude that BST4 is likely involved in the regulation of the lumenal pH over the first minute upon a dark-to-light transition.

The buildup of lumenal H^+^ concentration is usually accompanied by a buildup in the PMF across the thylakoid membrane, which is used by the ATPase to generate ATP. We used electrochromic shift (ECS) measurements (Bailleul et al. 2010) to measure the total PMF size, as well as initial PMF dissipation rate (g_H_^+^) (Fig. 8, F and G, Supplementary Fig. S21). Interestingly, in conditions where we hypothesize that the lumenal pH is higher in the *bst4* mutant (after 1 min of illumination), neither the PMF, g_H_^+^ nor proton flux (v_H_^+^) differed between *bst4* and its WT strain. We conclude that the changes induced by BST4 on the lumenal pH are either a small contribution to the PMF formation or are otherwise compensated for in the *bst4* mutant.

### We could not determine what species BST4 is permeable to

As a result of the NPQ difference seen between *bst4* and WT lines in response to illumination, we proposed that BST4 might be an anion channel involved in regulating the pH of the thylakoid lumen. Bestrophins are typically permeable to Cl^−^ and HCO_3_^−^. A plant thylakoid bestrophin, *At*VCCN1, is permeable to Cl^−^ and is also active in the first minute of illumination to modulate the lumenal pH, although *vccn1* mutants have lower NPQ (Herdean et al. 2016). Alternatively, like BST1-3, BST4 may be permeable to HCO_3_^−^ (Mukherjee et al. 2019). To determine which species BST4 may be permeable to, we expressed BST4 in Xenopus oocytes and measured currents in the presence of different anions (Supplementary Fig. S22). No currents for BST4 were detected in the presence of 100 mm KCl or 100 mm Na. HCO_3_. As some bestrophins are auto-inhibited by their C-terminus (Qu et al. 2006), we also tested 2 C-terminal truncations of BST4 (0-386 and 0-591), but no currents were detected. We also saw no currents for organic anions K-PEP and K-Gluconate, see Supplementary Fig. S22 for further details. Therefore, we were unable to conclude what BST4 is permeable to. BST4 may require certain conditions to be open that are not met in the oocyte system, such as post-translational modifications, a specific pH, a specific voltage, or an interaction partner. BST4 was found to be phosphorylated and had an oxidized methionine residue in its C-terminus (Bergner et al. 2015). Methionine oxidation can serve as channel-regulating post-translational modification (Ciorba et al. 1999), which would fit with the role that BST4 appears to have in preventing oxidative stress.

Based on these findings, we conclude that BST4 is a pentameric transmembrane channel found within the pyrenoid tubules but is not crucial for Rubisco matrix tethering. Rather, BST4 may be targeted to the tubules by the C-terminal RBMs to facilitate its role in the pyrenoid. Specifically, wetsiri propose that BST4 influences the ion homeostasis and subsequently the pH of the pyrenoid tubules through its function as an ion channel, particularly during fluctuating light. Future work is needed to determine what ionic species BST4 might transport, how it is regulated, and what other factors contribute to the role of BST4 in acclimation to light fluctuation.

## Materials and methods

### Phylogenetic analysis

Amino acid sequences for phylogenetic analysis were compiled by blasting BST4 (Cre06.g261750) in the NCBI database (Sayer et al. 2022) and the manual addition of other well-characterized bestrophin proteins including BEST1 *Homo sapiens* (XP_011543531.1), KpBEST *Klebsiella aerogenes* (WP_049046555.1), VCCN1 from Arabidopsis (*Arabidopsis thaliana*) (Q9M2D2), and BST1-3 Chlamydomonas (*Chlamydomonas reinhardtii*) (Cre16.g662600, Cre16.g663400, and Cre16.g663450). Sequences were aligned using a multiple sequence alignment program (multiple alignment using fast Fourier transform, MAFFT) (Katoh and Standley 2013) visually inspected and manually trimmed (specified). Finalized alignments were run through IQTREE webserver (Minh et al. 2020) to identify the most appropriate substitution model. Maximum likelihood trees were then generated in Geneious v11 using the PhyML 3.0 (Guindon et al. 2010) plugin with an Le Gascuel substitution model (Le and Gascuel 2008) with Gamma distribution (4 categories) and 500 bootstrap iterations. Full alignments are found in Supplementary Figs. S23 to S32.

### Alphafold structure prediction

Five chains of BST4 were submitted to Alphafold Multimer v2 with default settings. using the ColabFold server (Mirdita et al. 2022). All protein structure figures were generated using UCSF ChimeraX, developed by the Resource for Biocomputing, Visualization, and Informatics at the University of California, San Francisco, with support from National Institutes of Health R01-GM129325 and the Office of Cyber Infrastructure and Computational Biology, National Institute of Allergy and Infectious Diseases (Pettersen et al. 2021). The top-ranked model of 5 was used for figure generation.

### Generation of plasmids

All primers are listed in Supplementary Table S2. The plasmids for BST4 mutant complementation in Chlamydomonas were prepared using a recombineering method described previously (Emrich-Mills et al. 2021). BST4 is expressed under native promotor either without a tag or with a mScarlet-I C-terminal tag, and a hygromycin AphVII selection marker. The same approach was used to generate STA2-Venus lines in the *Crrbcs::AtRBCS* background (Genkov et al. 2010).

Plasmids for plant expression were generated using the MoClo system (Engler et al. 2014). For visualization, *BST4* was cloned from a synthesized g-block (IDT) with 35S promoter (pICH51277), mNeonGreen C-terminal tag (pICSL50015), heat shock protein (HSP) terminator and acceptor plasmid (pICH47732) in an L1 golden gate reaction. For transgenic seed selection, the BST4 expression cassette was combined with a pFAST-R cassette in an L2 golden gate reaction. For all other experiments, a no-tag BST4 construct was generated with the 35S protomer and HSP terminator parts and a Kanamycin resistance cassette was used for selection. Point mutations were introduced by PCR to generate a new L0 cds1 ns part.

Plasmids for Xenopus expression were generated by using the Gateway system by cloning the coding sequence for BST4 into a pGT vector (Grefen et al. 2010). *BST4* was amplified with Gateway adaptor sequences from an IDT with the PredAlgo (Tardif et al. 2012) predicted transit peptide removed from the N-terminal (sequence begins R35), and any subsequent mutations were made via PCR.

### Arabidopsis transformation

Arabidopsis was transformed by floral dip as previously described by Atkinson et al. (2016). BST4-mNeon primary transformants were screened for transgene insertion by seed fluorescence from pFAST-R and *BST4* expression was confirmed by checking for mNeon fluorescence and by immunoblot. BST4 no-tag primary transformants were screened using kanamycin resistance and immunoblot. Zygosity was checked via seed fluorescence from pFAST-R or kanamycin resistance.

### Chlamydomonas cell culture conditions and strain details

Chlamydomonas cultures were maintained as previously described by Ma et al. (2011). Tris-acetate-phosphate (TAP) and minimal (TP) media (acetate free) were prepared according to Sueoka (1960). TAP and TP agar plates for growth were made by adding 1.5% (w/v) agar. CMJ030 (CC-4533; *cw15, mt^−^)* and *bst4* (*BST4* knock-out LMJ.RY0402.159478) were obtained from the CLiP collection at the Chlamydomonas culture collection (Zhang et al. 2014; Li et al. 2019). This CLiP mutant has 2 other mapped CIB1 cassette insertions at loci Cre04.g230046 and Cre08.g367750. The insertion of the CIB1 cassette in *BST4* locus was confirmed by PCR amplifying the insertion locus from genomic DNA (Supplementary Fig. S3A) using loci-specific primers (forward GAGCTTCGTGGATGGATGTT and reverse GTATGAAGGTCACCGCCTGT) in parallel with a control locus (forward ATGCTTCTCTGCATCCGTCT and reverse ATGTTTTACGTCCAGTCCGC). The additional 2 insertions were also confirmed using loci-specific primers for Cre04.g230046 (forward TGTGCCTCTGTCAGTCTTGG and reverse TGCGTGGATGGGTAACAGTA), Cre08.g367750 (forward AATCAAGAAGCTTCCCAGCA and reverse CCTACCGCTATCTCAGCCAG) and STT7 locus as a control (forward GCACGAACCAAGACACACATAG and reverse GTAGACGATGTCACCGCACTT). Therefore, the *bst4* knock-out was complemented with *BST4* constructs described herein. All complemented lines were validated by immunoblotting of BST4 and specified epitope tags, described below (Supplementary Fig. S3, B to D).

### Chlamydomonas transformation

For each Chlamydomonas transformation, 28 ng kbp^−1^ of plasmid was linearized by restriction digest. Cells were grown to 2 to 4 × 10^6^ cells mL^−1^, harvested by centrifugation at 1,000*×g* for 10 min, and re-suspended in TAP with 40 mm sucrose at a concentration of 2 × 10^8^ cells mL^−1^. Linearized plasmid was mixed with 250 *μ*L of cells at 15 °C in a 0.4 cm gap electroporation cuvette and transformed immediately by electroporation using a Gene Pulser II (BioRad) set to 800 V and 25 *μ*F. Cells were recovered overnight in TAP sucrose while shaking gently (140 rpm) in the dark. Transformed cells were subsequently subjected to selection by growth on TAP agar plates with paromomycin (20 *μ*g mL^−1^) or hygromycin (25 *μ*g mL^−1^) which were kept in low light (5–10 *μ*mol photons m^−2^ s^−1^) until screening positive transformants.

### Generation of bst4 CRISPR knock-out lines

gRNAs targeting the BST4 locus were designed using the CHOPCHOP server, selected targets had zero mismatches, zero self-complementarity, and an efficiency calculated at 60%. Prior to transformation, WT Chlamydomonas were grown in 50 *μ*mol m^−1^ s^2^ on a 12:12 light/dark regime at 21 °C and shaking at 140 rpm. 3 h prior to transformation and the transition to the dark period, the temperature was increased to 33 °C. During this period, the ribonucleoprotein (RNP) mix was prepared, sgRNAs (IDT) (240 pmol) were incubated with Cas9 (60 pmol) and IDT Duplex Buffer (a total volume of 5 *μ*L per transformation) at 37 °C for 30 min.

Chlamydomonas cells were harvested by centrifugation 1,000× *g*, TAP removed, and re-suspended in TAP with 40 mm sucrose to a concentration of 2 × 10^8^ cells mL^−1^ and kept warm at 33 °C until just before electroporation. The RNP mix was combined with 500 ng of either AphVII/AphVIII per transformation at room temperature (RT). 115 *μ*L of cells were mixed with 10 *μ*L of RNP+ antibiotic resistance cassette in a 2 mm cuvette and immediately electroporated (conditions below) by a NEPA electroporator (Nepa Gene). The transformation was immediately recovered in 8 mL of TAP sucrose and incubated at 33 °C shaking overnight in the dark. The cells were plated on relevant selection plates and screened for cassette insert after approximately 2 wk of growth (Supplementary Fig. S16).

### Chlamydomonas growth assays

Spot tests: Cells were grown heterotrophically in TAP media. Once cultures reached 2 to 4 × 10^6^ cells mL^−1^, 1 × 10^6^ cells were harvested by centrifugation at 1,000*×g* for 10 min. Cells were washed and re-suspended at a concentration of 1 × 10^6^ cells mL^−1^ in TP media. Liquid cultures were spotted onto TP agar (1.5%) in 1,000, 100, and 10 cell spots at a range of pHs (specified). The plates were incubated in 3%, 0.04%, and 0.01% CO_2_ and illuminated under constant light at 400 *µ*mol photons m^−2^ s^−1^. Growth was monitored for up to 10 d. The light spectrum of the LED used is shown in Supplementary Fig. S33.

Fluctuating growth spot tests: Cells were inoculated on plates and grown in modified high salt (HS) minimal media [Ethylenediaminetetraacetic acid (85 μM), FeSO_4_∙7H_2_O (18 μM), ZnSO_4_∙7H_2_O (75 μM), H_3_BO_3_ (185 μM), MnCl_2_∙4H_2_O (26 μM), CuCl_2_∙2H_2_O (6.5 μM), Na_2_MoO_4_∙2H_2_O (5.5 μM), CoCl_2_∙6H_2_O (6.5 μM), K_2_HPO_4_ (1.65 mM), KH_2_PO_4_ (1.06 mM), NH_4_Cl (7.48 mM), CaCl_2_∙2H_2_O (0.34 mM), MgSO_4_∙7H_2_O (0.406 mM)] buffered in 3-(N-morpholino)propanesulfonic acid (9.56 mM) adjusted to pH 7.2. under 2% CO_2_ at 50 *µ*mol photons m^−2^ s^−1^. Once cells reached 2 to 4 × 10^6^ cells mL^−1^, cells were moved to air levels of CO_2_ for 8 h. Cells were re-suspended at a concentration of 1 × 10^6^ cells mL^−1^ in HS media. Liquid cultures were spotted onto HSM or TAP agar (1.5%) in 50, 5, 0.5, and 0.005 ng chlorophyll cell spots. The plates were incubated under indicated light regimes and CO_2_ levels.

Liquid growth: Cells were grown heterotrophically in TAP media. Once cultures reached 2 to 4 × 10^6^ cells mL^−1^, cells were harvested by centrifugation at 1,000*×g* and re-suspended at a starting concentration of 1 × 10^5^ cells mL^−1^ in TP media pH 7.4. Cultures were incubated in a CellDEG HDC 22.10 culture platform (CellDeg GMBH, Berlin) bubbled with 0.04% and 3% CO_2_, illuminated at 150 *µ*mol photons m^−2^ s^−1^ and consistently stirred at 180 rpm. Cell density and optical destiny (750 nm) measurements were taken daily for up to 10 d. Specific growth rates per hour were calculated using the following formula: *μ =* ln*(N*_2_/*N*_1_*)/t* whereby *N* = cell density.

Dot tests: Cultures were grown in a 96 format on agar plates and replicated by a Rotor+ (Singer Instruments) high throughput replication robot. The cultures were stamped onto pH 7.8 TP agar plates, incubated in 3%, 0.04%, and 0.01% CO_2_ and illuminated under constant light at a range of intensities (specified). Growth was monitored for up to 10 d.

### Phototaxis assays

Chlamydomonas cells were grown heterotrophically in TAP media until they reached 2 to 4 × 10^6^ cells mL^−1^ and harvested by centrifugation at 1,000*×g* for 10 min. Pelleted cells were either re-suspended in TP media or, for ROS manipulation assays, a phototaxis buffer described previously by Ueki et al. (2016) (5 mm Hepes pH 7.4, 0.2 mm EGTA, 1 mm KCl, and 0.3 mm CaCl_2_). The assays took place in 12-well dishes with a thin layer of TP agar (0.8%) on the well bottom and approximately 1.5 × 10^7^ cells in 400 *μ*L of homogenous suspension laid on top. The dishes were illuminated from one direction with 150 *µ*mol photons m^−2^ s^−1^ illumination for up to 3 h. Plates were imaged using a Flatbed Scanner at specified intervals.

### Quantification of H_2_O_2_

Cells were grown heterotrophically in TAP media. Once cultures reached 2 to 4 × 10^6^ celsl mL^−1^, cells were harvested by centrifugation at 1,000*×g* and re-suspended at a concentration of 2 × 10^6^ cells mL^−1^ in TP media pH 7.4, illuminated at 150 *µ*mol photons m^−2^ s^−1^ for 24 h and shaken at 140 rpm. For H_2_O_2_ quantification, 1 mL of culture was diluted at a 1:1 ratio with fresh TP media, containing 1 U of horseradish peroxidase and 5 *µ*M of Amplex Red (ThermoFisher) and incubated for 1 h (illuminated at 150 *μ*mol photons m^−2^ s^−1^, shaking 140 rpm). Cells were removed by centrifugation. The H_2_O_2_ of the media was immediately quantified using a ClarioStar Plate Reader Excitation/Emission 520/570 to 600 and compared against a linear H_2_O_2_ standard curve up to 5 *μ*M. Additional controls were included; some cells were treated with the ROS quencher DMTU at a final concentration of 150 *µ*M; and some with the PSII plastoquinone binding site blocker DCMU were dissolved in methanol at a final concertation of 10 *μ*M prior to H_2_O_2_ quantification (specified). All measurements were conducted with a minimum of 4 technical replicates. The data shown represent one of multiple experimental repeats conducted on different days with fresh cultures. All H_2_O_2_ concentrations were normalized to cell density, calculated as described previously, and chlorophyll content, described below.

Total chlorophyll was calculated by re-suspending 1 mL of harvested cells in 1 mL of methanol. All samples were protected from the light after menthol addition. After vortexing for 1 min to re-suspend the pellet and incubating for 10 min, the cells were removed by centrifugation. The absorbance of the supernatant was analyzed by spectrophotometer at 652 and 665 nm. Total chlorophyll was calculated using the formula below. All measurements are averaged from three technical replicates. Total chlorophyll (*µ*g/mL) = 22.12×Abs652 + 2.71×Abs665. Differences were tested for statistical significance by a one-way ANOVA and Tukey's post-hoc test, where significance was *P* < 0.05.

### Growth of Arabidopsis

Arabidopsis seeds were sown on moist F2 + S soil and stratified in the dark at 4 ℃ for 2 d. For growth experiments seeds were grown in a Percival SE-41AR3cLED chamber (CLF PlantClimatics GmbH, Wertingen, Germany) equipped with cool white LED lights under 12 h light (175 to 180 *µ*mol photons m^−2^ s^−1^)/12 h dark cycles at 21 °C, respectively and 70% relative humidity. Differences were tested for statistical significance by a one-way ANOVA and Tukey's post-hoc test, where significance was *P* < 0.05.

### Chlamydomonas confocal microscopy

Transgenic fluorescent strains were initially grown heterotrophically in TAP media until reaching 2 to 4 × 10^6^ cells mL^−1^ and re-suspended in TP media overnight prior to imaging. Cells were mounted on 8-well chamber slides and overlayed with 1.5% low melting point agarose made with TP medium. Images were collected on an LSM880 (Zeiss) equipped with an Airyscan module using a 63× objective. Laser excitation and emission setting for each channel used are set as below: Venus (Excitation: 514 nm; Emission 525 to 500 nm); mScarlet-I (Excitation: 561 nm; Emission 570 to 620 nm); Chlorophyll (Excitation: 633 nm; Emission 670 to 700 nm). Gain settings were experiment dependent but were set high enough such that some signal was visible in the background but low enough such that no pixels were saturated.

### Yeast-2-Hybrid

Yeast 2-hybrid to detect interactions between BST4 C-terminus and RbcS1 was carried out as described by He et al. (2020). BST4 C-terminus (amino acids 387-end) was cloned into the 2-hybrid vector pGBKT7 adapted to the MoClo syntax to create a fusion with the GAL4 DNA-binding domain. Point mutations were introduced by PCR into BST4 RBMs, which were then cloned into the same vector. Primers are listed in Supplementary Table S2. Mature CrRBCS1 was cloned into the vector pGADT7 to create a fusion with the GAL4 activation domain. Yeast cells were then co-transformed with binding and activation domain vectors. Successful transformants were cultured, diluted to an optical density at 600 nm (OD_600_) of 0.5 or 0.1, and plated onto SD-L-W (double drop out) and SD-L-W-H (triple drop out) media. The plates were imaged after 3 d and Supplementary Fig. S2 shows yeast spots from cultures diluted to an OD_600_ of 0.5.

### Slimfield microscopy

Chlamydomonas lines *bst4::BST4*-*mScarlet-I* and the unlabeled control line *bst4* were prepared overnight in TP media. Each was harvested and spotted onto a slide-mounted agar pad (GeneFrames, ThermoFisher), consisting of TP media with 1.5% low melting point agarose. Fluorescence imaging with single-molecule sensitivity was performed using a custom Slimfield microscope (Syeda et al. 2019). The setup used a high-magnification objective (NA 1.49 Apo TIRF 100× oil immersion, Nikon) and the detector was a Prime95B sCMOS camera (Teledyne Photometrics) operating in 12-bit “sensitivity” gain at a high total magnification of 53 nm/pixel. The samples were illuminated either in brightfield, or for Slimfield fluorescence in camera-triggered frames by a collimated 561 nm wavelength, Gaussian mode OPSL laser (Coherent, Obis LS) at a peak intensity of 5 kW/cm^2^ at the sample plane. This beam was tilted into a highly inclined and laminated optical sheet configuration (Payne-Dwyer and Leake 2022) to reduce out-of-focus excitation while retaining quantitative molecular sensitivity. The fluorescence image was split into 2 parallel channels comprising emission bandpass filters (Semrock BrightLine): one with a 585/15 emission filter (central wavelength/spectral bandwidth in nanometers) optimized to isolate the mScarlet-I signal, and a second with a 525/25 emission filter, used only to indicate autofluorescence background. The total length of each acquisition sequence was ∼5 s; sufficient to observe the full course of mScarlet-I photobleaching, from the initial unbleached state to single-molecule blinking, while also rapid enough (10 ms exposure/frame at 180 frames/s) to capture the motion of individual molecular assemblies.

### Single particle tracking and molecular counting

Slimfield image sequences were segmented manually in ImageJ to include only the pyrenoids in downstream analysis. The centroid positions of fluorescent tracks were identified from local intensity maxima in each frame using ADEMScode software in MATLAB (Wollman et al. 2022). The summed intensity of each candidate track was calculated in each frame by adding all pixel values within 5 pixels of the centroid, then subtracting the local background averaged between 5 and 8 pixels from the centroid. Candidates with a summed intensity <0.4× the standard deviation in the background region were discarded.

Fluorescent proteins are known to exhibit a characteristic integrated intensity per molecule under stable Slimfield imaging conditions and within the quasi-uniformly illuminated area within half the beam waist (Shepherd et al. 2021). After sufficient photobleaching of mScarlet-I in the Slimfield image sequences, only step-like blinking was observed at the end of each track. The modal integrated intensity of these steps was used to estimate this characteristic single-molecule brightness, equivalent to 56 ± 9 photoelectrons per frame per molecule.

At the start of each track, we obtained an initial integrated intensity (independent of photobleaching) by linearly extrapolating the summed intensity backward over the first 4 frames of the exposure. This initial intensity was then divided by the characteristic brightness of a single mScarlet-I to estimate the number of molecules, or stoichiometry, in that track. This estimate was precise enough to detect stoichiometry steps of up to 12 tagged molecules without ambiguity.

Stoichiometry distributions may exhibit peaks that are separated by a characteristic interval. The smallest consistent interval between peaks can be used to infer the size of a physical repeat unit or “periodicity” within assemblies (Hunter et al. 2022; Payne-Dwyer et al. 2022). A kernel width of 0.7 molecules was chosen to generate the stoichiometry distribution (Fig. 2F), reflecting the background standard deviation. Peaks were pinpointed using MATLAB's *findpeaks*.

The intervals between all peaks for each acquisition were aggregated across the pyrenoid population, weighted by inverse square root distance (thereby accounting for shot noise in broader intervals). A second distribution (Fig. 2G) was then generated from this weighted population of intervals. The kernel width in this estimate was 0.7 molecules multiplied by the square root of the mean stoichiometry divided by the root number of intervals (thereby accounting for shot noise in intervals between peaks of higher stoichiometry). The periodicity was then reported as the mode of this distribution and its 95% confidence interval.

### Arabidopsis confocal microscopy

Small sections of 3- to 4-week-old leaf tissue (∼5 to 10 mm^2^) were adhered to slides using double-sided tape with basal side up. A ×40 water immersion objective lens was used. Samples were excited by 488 nm at 1% laser power, chlorophyll autofluorescence was collected at 680 to 750 nm, and mNeonGreen fluorescence at 503 to 532 nm. For dual-tagged lines, we used sequential acquisition to minimize bleed-through. mCherry was excited using the 542 nm laser and emission collected at 601 to 620 nm and mNeon as before. Gain settings were experiment dependent but were set high enough such that some signal was visible in the background but low enough such that no pixels were saturated. Images were acquired using the SP8 Confocal system and Leica LAS AF software (http://www.leica-microsystems.com/). Figures were prepared using ImageJ (http://fiji.sc/Fiji).

### Immunoblot detection

Two leaf disks (6 mm diameter) were harvested and immediately frozen in liquid nitrogen. Two steel balls (3 mm) were added and tissue was homogenized using a tissue-lyser twice for 30 Hz for 30 s. A 4-time volume of cold extraction buffer (20 mm Tris-HCl pH = 7.5, 5 mm MgCl_2_, 300 mm NaCl, 5 mm DTT, 1% (v/v) Triton X-100, 1× Protease inhibitor (Roche)) was added and samples vortexed for 30 s. Samples were solubilized on ice for 5 min and then centrifuged at 5,000*×g* for 5 min at 4 ℃. 17.5 *µ*L of supernatant was used to make up 1× LDS and 100 *μ*M DTT. 20 *µ*L was loaded on a Novex 4% to 12% Bis-Tris Mini Gel, (Thermo Fisher, Catalogue number: NP0322BOX). The gel was run at 150 V for 60 min. Proteins were transferred to a nitrocellulose membrane using an iBlot 2, program 0. The membrane was probed with primary antibody in 5% (w/v) milk in 1× Tris-buffered saline with 0.1% (v/v) Tween 20 detergent at the following dilutions: BST4 (1:1,000; generated for this study, peptide from C-terminus: SDTELSEANRPRTRPDWRN) (YenZym, Antibodies LLC, USA), AtpB (1:2,000; Agrisera:AS05085), RbcL (1:1,000; kind gift from Griffiths lab), CP43 (1:3,000; Agrisera: AS111787), PsaB (1:1,000; Agrisera: AS10695), and PsbO (1:2,000; Agrisera:AS06142-33). Secondary antibody (goat ɑ-rabbit IR-800; Li-COR: 925-32211). Membrane was imaged using the Li-COR Odyssey CLx scanner.

In order to quantify BST4 protein in Chlamydomonas lines, cells were grown in TP media at ambient CO_2_ until reaching 2 to 4 × 10^6^ cells mL^−1^. Cells were harvested by centrifugation at 1,000*×g* for 10 min, normalized to Chl content and re-suspended in the extraction buffer described above. Samples were freeze-thawed 3 times and spun at 20,000*×g* for 20 min at 4 ℃. Protein extractions containing 5 *µ*g of Chl with 1× sodium dodecyl sulfate (SDS) loading buffer were boiled at 100 ℃ for 5 min and loaded onto a 4% to 20% polyacrylamide gel (Mini Protean TGX, Biorad Laboratories). Proteins were transferred to a PVDF-FL membrane on a Biorad semidry blotting system. BST4 primary antibody was used as described above alongside alpha-tubulin primary antibody raised in mouse (Agrisera), as a loading control. Anti-rabbit and anti-mouse fluorescent secondary antibodies, Invitrogen AlexaFluor 488 and 555 respectively, were used at a 1:20,000 dilution. Immunoblots were imaged using an Amersham typhoon 5 scanner with 488 and 535 excitation lasers and Cy2 and Cy3 emission filters. BST4 band fluorescent intensity was quantified using FIJI (Image J) (Schindelin et al. 2012) and normalized to alpha-tubulin loading control. All Chlamydomonas lines for quantification were extracted and analyzed in triplicate. Differences were tested for statistical significance by a paired two-tail *t*-test, where significance was *P* < 0.05. For LHCSR3 protein quantification, cells were seeded at 0.1 OD_750_ in TAP media for 4 d and then switched to TP media at a concentration of 30 *µ*g Chl mL^−1^ and exposed to 150 *µ*mol photons m^−2^ s^−1^ for 3 h. Protein was extracted according to Burlacot et al. (2022) and separated by SDS-PAGE as described for BST4 immunoblots.

Oocytes for expression of BST4 were collected after recording and prepared for immunoblot as described by Lefoulon et al. (2018). The BST4 primary antibody was used as described above and the secondary antibody used was horseradish peroxidase-coupled goat, anti-rabbit (dilution 1:10,000 Abcam). Proteins were detected with ECL Advance kit (GE Healthcare, Poole, UK). Uncropped versions of all immunoblots are compiled in Supplementary Figs. S34 to S36.

### Blue Native-PAGE

A crude thylakoid enrichment was performed according to Aro et al. (2004). Thylakoid membranes were solubilized in 0.5% n-dodecyl-ß-D-maltoside, 1× Native-PAGE buffer (Thermo: BN2003), 1× complete protease inhibitor tab (Roche; 10× stock made by dissolving 1 tablet in 1 mL dH_2_O) for at a concentration of 0.8 *µ*g Chl/*µ*L for 15 min on ice. Unsolubilized material was removed by 2 rounds of centrifugation at 17,000*×g* at 4 °C for 15 min. 19.5 *µ*L of supernatant was combined with 0.5 *µ*L of Coomassie additive and loaded immediately onto a 4% to 16% Bis-tris gel (Thermo: BN1002BOX). Electrophoresis was performed at RT at 150 V for 90 min. Cathode buffer was swapped from dark to light when the dye front was a third way through the gel. Separated proteins were transferred to a nitrocellulose membrane by electrophoresis at 100 V for 90 min at 4 ℃.

Proteins were visualized using chemiluminescence. Secondary antibody (goat ɑ-rabbit, HRP; 1:10,000; Abcam: ab6721). Chemiluminescence substrate SuperSignal West Pico PLUS (ThermoScientific, ref number: 34579) according to manufacturer's instructions. Chemiluminescence was detected using clear blue X-Ray Film CL-Xposure^TM^ Film (ThermoScientific, ref number: 34090).

### Chlorophyll fluorescence measurements in Chlamydomonas for Fig. 8

To measure PSII activity in Chlamydomonas, cells were grown in HS media under 80 *µ*mol photons m^−2^ s^−1^ for 3 d at 120 rpm (in a Multitron, Infors-ht, light spectrum in Supplementary Fig. S24) to reach active growth phase and then maintained at ∼10 *µ*g Chl mL^−1^. Biological replicates were defined as cultures grown in separate flasks. 2 mL of cells were added to a cuvette and bubbled with air (10 cc/min) and continuous stirring. Cells were incubated in the dark for 5 min before recording Chl fluorescence using DUAL-PAM-100 (Walz, Effeltrich, Germany). A saturating pulse of 8,000 *µ*mol photons m^−2^ s^−1^ of 300 ms was applied to the samples for determination of the maximal fluorescence yield in the dark state (*F_m_*) and maximal fluorescence yield during the period with actinic light (*F_m_*’). Minimal fluorescence yields in the dark were also determined in prior to the first saturating flash (*F_o_*). The maximal quantum efficiency of PSII was calculated as (*F_m_^’^*−*F)/F_m_* where *F* is the stationary fluorescence. NPQ was calculated as (*F_m_*−*F_m_’*)/*F_m_*’. Far-red light (4 *µ*mol photons m^−2^ s^−1^) was used throughout the entire experiment to limit state transition from contributing to the NPQ. NPQ and Y(II) were calculated based on changes in Chl fluorescence as (*F_m_–F_m_’*)/*F_m_*’ and (*F*_m_’–*F*)/*F_m_*’, respectively, according to Genty et al. (1989). When indicated, cells were supplemented with a final concentration of 500 *µ*M HCO_3_^−^ at the beginning of the dark adaptation. 1-qL values were calculated directly using the qL parameter calculated as (*F_m_’–F*)*F_o_’/(*F_m_’– F_o_*’)*F (Kramer et al. 2004) *where F_o_*’ represents the minimum fluorescence yield in the light-adapted state and can be estimated as *F_o_*’=*F_o_/(F_v_/F_m_ + F_o_/F_m_’)*. Relative electron transport rate was calculated by multiplying the electron transport rate of a given strain by the ratio of its chlorophyll content relative to the WT strain. Differences were tested for statistical significance by a one-way ANOVA and Tukey's post-hoc test, where significance was *P* < 0.05.

### ECS in Chlamydomonas for Fig. 8

ECS in Chlamydomonas was assessed by measuring the absorbance changes of cells at 520 and 545 nm using a JTS-100 spectrophotometer (BioLogic). Cells were grown and prepared as for Chl fluorescence experiments described previously except cells were re-suspended to 150 *µ*g Chl mL^−1^ before being loaded into a custom vertical light path cuvette. Cells were dark adapted for 1 min and then exposed to 890 *μ*mol photons m^−2^ s^−1^ red light (630 nm) for 1 min. The light was switched off and decay kinetics was measured. ECS signal was calculated as the difference between absorbance changes measured at 520 and 545 nm. For each biological replicate, 3 technical replicates were taken and averaged. PMF size was calculated as the difference between the ECS signal in light and the minimum value of the ECS signal immediately after the light was turned off. The g_H_^+^ parameter was calculated as 1/*τ*, where *τ* is the time constant for decay during the first 100 ms (Cruz et al. 2005). Differences were tested for statistical significance by a one-way ANOVA and Tukey's post-hoc test, where significance was *P* < 0.05.

### Chlorophyll fluorescence measurements in Chlamydomonas for Supplementary Fig. S14

To measure PSII activity in Chlamydomonas, cells were grown in TAP in low light (20 *µ*mol photons m^−2^ s^−1^) for 4 d at 50 rpm to reach logarithmic phase. Cells were then washed, re-suspended to 30 *µ*g Chl mL^−1^ in TP media and exposed to 150 *µ*mol photons m^−2^ s^−1^ light for 3 h, followed by 1 h incubation in darkness at 50 rpm before recording Chl fluorescence using DUAL-PAM-100 (Walz, Effeltrich, Germany). A saturating pulse of 3,000 *µ*mol photons m^−2^ s^−1^ of 800 ms was applied to the samples in a cuvette under continuous stirring for determination of the maximal fluorescence yield in the dark state (*F_m_*) and maximal fluorescence yield during the period with actinic light (*F_m_’*). The maximal photochemical efficiency of PSII (*F_v_/F_m_*) was calculated. NPQ was determined from slow kinetics during actinic illumination at 1,500 *μ*mol m^−2^ s^−1^ for 17 min followed by 5 min of dark relaxation. NPQ and Y(II) were calculated based on changes in Chl fluorescence as (*F_m_–F_m_’*)/*F_m_*’ and (*F*_m_’–*F*)/*F_m_*’, respectively, according to Genty et al. (1989). Differences were tested for statistical significance by a one-way ANOVA and Tukey's post-hoc test, where significance was *P* < 0.05.

### ECS in Chlamydomonas for Supplementary Fig. S17

ECS measurements in Chlamydomonas were carried out using the Dual-PAM-100 equipped with a P515/535 module (Walz). Cells grown and prepared as for Chl fluorescence experiments described previously were layered on a glass slide and exposed to actinic red light for the given period. The light was switched off and decay kinetics were measured. PMF size was calculated as the difference between the ECS signal in light and the minimum value of the ECS signal immediately after the light was turned off. Calculation of ΔpH and ΔΨ was performed using the steady-state time point of the ECS signal in darkness (Cruz et al. 2001). Before each ECS measurement, a 3 saturating 50-μs actinic red flashes of 200,000 *μ*mol photons m^−2^ s^−1^ was applied to determine the ECS_ST_; subsequently, the ECS_ST_ amplitude was used to normalize the ECS signal before the calculation of PMF size and partitioning values. To determine H^+^ conductivity (g_H_^+^), the light was switched off at specific time points to record the ECS signal decay during 620 ms dark intervals. The g_H_^+^ parameter was calculated as 1/*τ* (time constant for decay during the first 100 ms (Cruz et al. 2005)). The total proton flux across the membrane was calculated as ν_H_^+^ = PMF *× g*_H_^+^ (Cruz et al. 2001). Differences were tested for statistical significance by a one-way ANOVA and Tukey's post-hoc test, where significance was *P* < 0.05.

### Chlorophyll fluorescence measurements for Arabidopsis

Plants were grown for 8 wk on S-Hasselfors soil in a Percival AR-82L chamber (CLF Plant Climatics, Wertingen, Germany) using 12 h light (180 *µ*mol photons m^−2^ s^−1^)/12 h dark cycles at 21 °C/19 °C, respectively, and 70% relative humidity. Slow kinetics of Chl *a* fluorescence induction were recorded with a pulse-amplitude modulated fluorometer DUAL-PAM 100 equipped with DUAL-DB and DUAL-E emitter/detector module (Walz) on attached leaves of 30 min dark-adapted plants using actinic red light of 830 *μ*mol photons m^−2^ s^−1^ for 10 min, followed by a 5 min dark period. The saturating pulse applied was 5,000 *µ*mol photons m^−2^ s^−1^ and of 800 ms duration. NPQ and (Y(II)) were calculated based on changes in Chl fluorescence as (*F_m_*–*F_m_*’)/*F_m_*’ and (*F_m_*’–*F*)/*F_m_*’, respectively (Genty et al. 1989). Separate plants were used for each biological replicate. Differences were tested for statistical significance by a one-way ANOVA and Tukey's post-hoc test, where significance was *P* < 0.05.

### ECS for Arabidopsis

ECS was recorded with a DUAL-PAM 100 system equipped with a P515/535 emitter/detector module (Walz). First, plants were dark adapted for 30 min, then illuminated with actinic red light at 830 *µ*mol photons m^−2^ s^−1^ for 3 min followed by a 60 s dark period in which the ECS decay kinetics were recorded. Before each measurement, 3 pulses of 5 *µ*s and 200,000 *µ*mol photons m^−2^ s^−1^ were applied to determine ECS_ST_, which was used to normalize the ECS_T_ values of each measurement. Differences were tested for statistical significance by a one-way ANOVA and Tukey's post-hoc test, where significance was *P* < 0.05.

### Sedimentation of proto-pyrenoid

Cross-linked samples were prepared by vacuum infiltrating intact leaves 1% formaldehyde prior to sedimentation 200 mg of leaf tissue was flash frozen in liquid nitrogen and ground by bead beating twice at 30 Hz for 30 s. Four times volume of extraction buffer (50 mm HEPES-KOH pH 7.5, 17.4% (v/v) glycerol, 2% (v/v) Triton X-100, cOmplete protease inhibitor tab) was added and the sample was mixed by bead beating again. Extract was filtered through one layer of miracloth. A small aliquot of filtered extract was saved as the input. The extract was then centrifuged at 500*×g* for 3 min at 4 ℃ and the pellet was discarded. The supernatant was centrifuged at 500*×g* for 12 min, 4 ℃. The pellet was washed once with extraction buffer and then centrifuged again. The pellet was re-suspended in 100 *µ*l extraction buffer and then centrifuged for a further 5 min. The pellet was finally re-suspended in 25 *µ*l extraction buffer. Fractions were made up in 1× LDS loading buffer and 200 mm DTT. Ten microliters of each fraction were subjected to SDS-PAGE (NuPAGE 4% to 12% Bis-Tris Mini Gel, Thermo Fisher, Catalog number: NP0322BOX) at 150 V for 60 min.

### Chloroplast fractionation

In order to biochemically localize transgenically expressed BST4 in Arabidopsis, chloroplasts were fractionated as described by Herdean et al. (2016) using 100 g of leaf tissue from 4- to 5-week-old BST4 transgenic plants. The stromal fraction was concentrated using a 10,000 molecular weight cut off centrifugal concentrator (Sartorius Stedim Biotech GmbH, product number: VS1502).

### Arabidopsis protoplast isolation

Protoplasts were released from 1 mm strips of Arabidopsis leaf tissue by enzymatic digestion in mannitol magnesium (MGG) buffer [4 mM 2-(N-morpholino)ethanesulfonic acid (MES), 0.4 M mannitol and 15 mM MgCl_2_ at pH 5.7] for 3 h in the dark [1× MGG, 1.5% (w/v) Cellulase R10, 0.4% (w/v) macerozyme R10 (Yakult honsha, Tokyo, Japan)] followed by shaking at 80 rpm for 5 min. Protoplasts were isolated by passing the digest mixture through a 75 *μ*m nylon mesh and washing with 1× MGG.

### Electron microscopy of Chlamydomonas

High (3%) and low (0.04%) CO_2_ acclimated cells were harvested by centrifugation (1,000*×g*, 4 min, 20 °C). Primary fixation was performed in 1.25% (v/v) glutaraldehyde in 50 mm sodium cacodylate (pH 7.15) in TP medium for 15 min followed by 2.5% (v/v) Glutaraldehyde in 50 mm cacodylate for 2 h. Fixed samples were washed 3 times with 50 mm sodium cacodylate by centrifugation. Samples were then osmicated with 1% OsO4 in 50 mm sodium 25 cacodylate for 1 h on ice and washed with de-ionized water. Samples were block stained in 1% uranyl acetate in the dark for 1 h. Samples were washed twice with dH_2_O and twice with 50 mm sodium cacodylate. Fixed samples were dehydrated in an acetone series (25%, 50%, 75%, 90%, and 100%) ∼20 min each step. Dehydrated samples were infiltrated with Spurr's resin by incubating in 25%, then 50% Spurr resin in acetone for 30 min, and transferred to 75% for 45 min at RT. They were then incubated in 100% Spurr resin overnight before polymerizing at 70 °C for 24 h. Sections ∼70 nm thick were collected on copper grids and stained with saturated uranyl acetate and lead citrate. Images were collected with a FEI Tecnai 12 BT at 120 kV using a Ceta camera.

### Electron microscopy of Arabidopsis

Leaves were cut into small 5 mm strips and fixed in 4% (v/v) paraformaldehyde/0.5% (v/v) glutaraldehyde in 0.05 m sodium cacodylate (pH = 7.2) by vacuum infiltration 3 times for 15 min and incubation at 4 °C overnight with gentle agitation followed by dehydration in increasing amounts of ethanol 50/70/80/90/100% (v/v) 1 h each then overnight rotation. 100% ethanol was repeated 3 times, 1 h each and a final over night at 4 °C. Samples were then fixed in London resin by infiltrating with increasing concentration (50/70/100%(v/v)) with a repeat of 100% and then polymerized overnight at 50 °C. Ultrathin sections were cut and mounted onto plastic-coated copper grids. Grids were stained with 2% (w/v) uranyl acetate and visualized by the TEM.

### Protease protection assay

Investigation of the orientation of BST4 in isolated thylakoid membranes was conducted as described by Stepien and Johnson (2018). Briefly, trypsin is made up in 50 mm acetic acid according to the manufacturer's instructions (Thermo Scientific, ref number: 90057). To disrupt the thylakoid membrane and allow degradation to lumen-facing peptides, 1% (v/v) Triton X-100 was added, and tubes were gently agitated prior to the addition of trypsin.

### Xenopus oocyte electrophysiology

Destination clones containing BST4 for Xenopus expression were linearized with EcoRI, before proceeding to the in vitro transcription (mMessage mMachine T7 transcription kit, Thermofisher Scientific, ref number AM1344). Stage IV oocytes were injected with 20 ng of RNA per oocyte. Measurements of ion transport were done by voltage clamp using an Axoclamp 2B amplifier (Axon Instruments, Foster City, CA) (Leyman et al. 1999; Lefoulon et al. 2014). They were performed under perfusion of either 100 mm KCl, K-HCO_3_, Na-HCO_3,_ K-PEP, or K-Gluconate, with 1 mm CaCl_2_, 1.5 mm MgCl_2_, and 10 mm HEPES-NaOH, pH 7.3. Recordings were obtained and analyzed using Henry IV software (Y-Science, Glasgow, UK). BST4 expression in oocytes was validated by immunoblotting (Supplementary Fig. S22B), as described earlier.

### Accession numbers

Sequence data from this article can be found in the GenBank/EMBL data libraries under the following accession number: Bestrophin-like protein 4/Rubisco binding membrane protein 1: Cre06.g261750.

## Supplementary Material

kiae450_Supplementary_Data

## Data Availability

The data underlying this article will be shared on reasonable request to the corresponding authors.

## Supplementary data

The following materials are available in the online version of this article.

**Supplementary Figure S1.** BST4 structure and sequence compared to other bestrophins.

**Supplementary Figure S2.** Supporting Figs. 4 and 5, Interaction between BST4 and RBCS.

**Supplementary Figure S3.** Validation of Chlamydomonas BST4 lines.

**Supplementary Figure S4.** Supporting Fig. 3, prediction of BST4 membrane topology.

**Supplementary Figure S5.** Phenotype of BST4 transgenic Arabidopsis line.

**Supplementary Figure S6.** Photosynthetic measurements of BST4 transgenic Arabidopsis lines 2 and 3.

**Supplementary Figure S7.** Supporting Fig. 4, pyrenoid morphology in bst4 vs WT.

**Supplementary Figure S8.** Supporting Fig. 5, localization of C-terminally truncated BST4 in WT background.

**Supplementary Figure S9.** Supporting Fig. 5, localization of proteins in a pyrenoid matrix-less background.

**Supplementary Figure S10.** Supporting Fig. 6, spot test of WT, bst4, and BST4 complemented lines under CCM induced conditions.

**Supplementary Figure S11.** Supporting Fig. 6, phototaxis and ROS assay.

**Supplementary Figure S12.** Supporting Figs. 6 and 8, chlorophyll fluorescence measurements.

**Supplementary Figure S13.** Supporting Figs. 6 and 8, Chlorophyll fluorescence measurements.

**Supplementary Figure S14.** Supporting Fig. 6, Chlamydomonas bst4 mutant has an enhanced NPQ and proton conductance under high light and limiting Ci conditions.

**Supplementary Figure S15.** Supporting Fig. 7, spot test of BST4 strains from Fig. 7 including CRISPR-generated.

**Supplementary Figure S16.** Generation and validation of bst4 CRISPR knock-out lines.

**Supplementary Figure S17.** Supporting Fig. 8, chlorophyll fluorescence measurements.

**Supplementary Figure S18.** Supporting Fig. 8, NPQ with supplemented bicarbonate.

**Supplementary Figure S19.** Supporting Fig. 8, chlorophyll fluorescence measurements of bst4 CLiP and CRISPR mutants.

**Supplementary Figure S20.** Supporting Fig. 8, the effect of nigericin on NPQ.

**Supplementary Figure S21.** Supporting Fig. 8, ECS traces across four biological repeats.

**Supplementary Figure S22.** No currents were detected for BST4 with any anions tested in Xenopus oocytes.

**Supplementary Figure S23.** Sequences used to generate MAFFT alignments used to generate the phylogenetic trees included in this manuscript Part 1 of 6.

**Supplementary Figure S24.** Sequences used to generate MAFFT alignments used to generate the phylogenetic trees included in this manuscript Part 2 of 6.

**Supplementary Figure S25.** Sequences used to generate MAFFT alignments used to generate the phylogenetic trees included in this manuscript Part 3 of 6.

**Supplementary Figure S26.** Sequences used to generate MAFFT alignments used to generate the phylogenetic trees included in this manuscript Part 4 of 6.

**Supplementary Figure S27.** Sequences used to generate MAFFT alignments used to generate the phylogenetic trees included in this manuscript Part 5 of 6.

**Supplementary Figure S28.** Sequences used to generate MAFFT alignments used to generate the phylogenetic trees included in this manuscript Part 6 of 6.

**Supplementary Figure S29.** Sequences used to generate MAFFT alignments used to generate the phylogenetic trees included in this manuscript Part 1 of 4.

**Supplementary Figure S30.** Sequences used to generate MAFFT alignments used to generate the phylogenetic trees included in this manuscript Part 2 of 4.

**Supplementary Figure S31.** Sequences used to generate MAFFT alignments used to generate the phylogenetic trees included in this manuscript Part 3 of 4.

**Supplementary Figure S32.** Sequences used to generate MAFFT alignments used to generate the phylogenetic trees included in this manuscript Part 4 of 4.

**Supplementary Figure S33.** Light spectra of growth lights used in this work.

**Supplementary Figure S34.** Uncropped immunoblots from Supplementary Fig. S3.

**Supplementary Figure S35.** Uncropped immunoblots from Figs. 2, 3, 4, and 8.

**Supplementary Figure S36.** Uncropped immunoblots from Supplementary Figs. S5, S14 and S16.

**Supplementary Table S1.** Growth rate of *bst4* compared to WT.

**Supplementary Table S2.** Primers used for cloning.

## Dive Curated Terms

The following phenotypic, genotypic, and functional terms are of significance to the work described in this paper:

LHCSR3 Gramene: XP_001696138.1
LHCSR3 Araport: XP_001696138.1
