# Supplementary material for: Bestrophin-like protein 4 is involved in photosynthetic acclimation to light fluctuations in Chlamydomonas
Source: Plant Physiol. 2024 Sep 6;196(4):2374–94. doi: 10.1093/plphys/kiae450 (PMC11638005; doi:10.1093/plphys/kiae450)
Supplement: kiae450_Supplementary_Data [file kiae450_supplementary_data.zip › 20240904 Supplemental Figures- no tracked changes.docx]

**Supplemental Figures**

**Supplemental Figure S1. Supporting Figure 1, BST4 structure and sequence compared to other bestrophins.** Phylogenetic analysis of the full length Chlamydomonas BST4 amino acid sequence (bold). The evolutionary history of BST4 was inferred by using the maximum likelihood method based on the Le and Gascuel substitution model with discrete Gamma distribution (5 categories) and 500 bootstrap replicates. The tree is drawn to scale, with branch lengths measured in the number of substitutions per site.

**Supplemental Figure S2. Supporting Figure 4 and 5, Interaction between BST4 and RBCS. A.** Yeast-2-hybrid experiment. The disordered region of BST4 C-terminus (amino acid 387-end) and with residues WR from RBMs are changed to EE. Abbreviations: AD, activation domain; BD, binding domain; DDO, double drop out media; TDO, triple drop out media. Growth on TDO indicates an interaction. **B**. FRET efficiency between BST4-mScarlet-I and CrRBCS-Venus. Sensitized-FRET measurement on dual-tagged cells (BST4-Venus and RBCS1-mCherry) showed a moderate FRET efficiency of 35%. Error bar is ±SEM (n=10).

**Supplemental Figure S3. Validation of Chlamydomonas BST4 lines**. **A.** PCR amplification of the mapped CIB1 insertion sites and control loci of *bst4* (LMJ.RY0402.159478) and wild type (WT) control strain (CMJ030 (CC-4533; cw15, mt -) gDNA to confirm the insertion of the CIB1 cassettes in *bst4.* **B.** Immunoblots confirming the confirming the expected production of BST4 in WT, *bst4, bst4::BST4, bst4::BST4-mScarlet-I* and *bst4::BST-truncated* Chlamydomonas lines. **C.** Immunoblot blot used to quantify the amount of BST4 protein detected in WT and *bst4::BST4.* **E.** Immunoblot was used for the quantification of fluorescent intensity as a measure of BST4 protein abundance. Plot represents the mean ± SD. All fluorescent intensity measurements were normalized to respective alpha-tubulin loading controls and conducted in triplicate. No statistical difference was observed between WT and *bst4::BST4* BST4 protein abundance (Paired two-tail t-test *p =* 0.22, n=3).


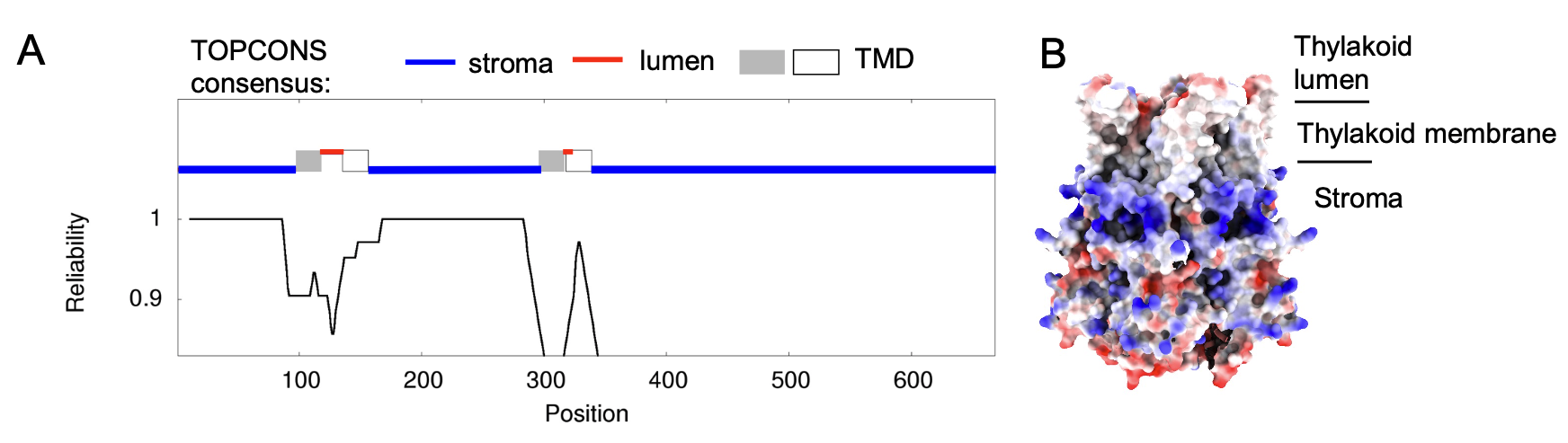
 **Supplemental Figure S4. Supporting Figure 3, Prediction of BST4 membrane topology. A.** Topology predictions of BST4 based on TOPCONS consensus (a web server for combined membrane protein topology and signal peptide prediction) (Tsirigos et al., 2015). Topology manually annotated to show stroma and lumen based on the ‘positive-stroma’ rule (Gavel et al., 1991) **B.** Surface charge of BST4 homo-pentamer Alphafold model. Thylakoid membrane marked based on the Cryo-EM structure of bestrophin channel VCCN1 in a lipid nanodisc (template 7ek2.1) and predicted topology by TOPCONS. Disordered N-terminal and C-terminal regions removed for clarity. Blue and red are positive and negative, respectively.

**Supplemental Figure S5. Phenotype of BST4 transgenic Arabidopsis line. A.** Immunoblot against BST4 of proteins extracted from BST4 no tag lines and Azygous segregants (Az). S2_Cr_ is the parent line. (S2_Cr_ is an Arabidopsis line where ~50% of the Rubisco small subunits are heterologously expressed *Cr*RbcS1). **B.** Fresh weight of 28-day old rosettes, bars represent mean weight for each genotype n=18-21. Different letters indicate statistically significant difference among the genotypes (one-way ANOVA test, followed by Turkey’s post hoc test, P < 0.05).

**Supplemental Figure S6. Photosynthetic measurements of BST4 transgenic Arabidopsis lines 2 and 3. A.** *F*_v_/*F*_m_ values measured on attached, 30 min dark adapted leaves of 8-week-old plants (n=15-21). The letters in indicate nonsignificant differences between plants expressing BST4 and their azygous segregants using Tukey post hoc test (P > 0.05). **B.** Non-photochemical quenching (NPQ) as a measure of photoprotection and **C.** photosystem II quantum yield (Y(II)) were recorded during 10 min of illumination at 830 µmol photons m^−2^ s^−1^ followed by a 5-minute dark period. Data are presented as means ± SEM (n=4-6). Asterisks indicate statistical difference between plants expressing BST4 and their Azygous (Az) segregants according to unpaired t-test (P ≤ 0.05). **D.** Proton motive force (PMF) size and **E.** partitioning to pH gradient (∆pH) after 3 min illumination at 830 µmol photons m^−2^ s^−1^. Data are means ± SEM (n=5-6). The letters in E and F indicate non-significant differences between plants expressing BST4 and their azygous segregants according to unpaired t-test (P > 0.05).

**Supplemental Figure S7. Supporting Figure 4. Pyrenoid morphology in bst4 vs WT. A.** The pyrenoid area to cell area ratio of wild type (WT) control strain (black) and *bst4* (white) strains at high (3%) and low (0.04%) CO_2_ concentrations. Each bar represents the mean value ± SEM (n=40–50). The data for WT and *bst4* strains were compared using a two sample T-test. The p-values (showing no significant difference) are displayed above the bars**. B.** Pyrenoid area vs cell area of WT and *bst4* at 0.04% [CO_2_].

**Supplemental Figure S8. Supporting Figure 5, Localization of C-terminally truncated BST4 in wild type (WT) background.** BST4_ΔCterm_-Venus fluorescence shown in green and chlorophyll autofluorescence in magenta. Scale is bar 1 µm and applies to all images. A diagram of the topology of BST4 is on the left-hand side. The red cross indicates C-terminal truncation of BST4.

**Supplemental Figure S9. Supporting Figure 5, Localization of proteins in a pyrenoid matrix-less background. A.** Diagram showing a section of the *Crrbcs::AtRBCS* - a Chlamydomonas line where the small subunit of Rubisco is replaced with heterologously expressed Arabidopsis small subunit that has no pyrenoid matrix (*-matrix*). Cell is shown in gray and the chloroplast in dark gray. The nascent pyrenoid tubules (pt) are shown in green, starch granules (sg) in white. **B.** Confocal image of starch binding protein STA2 fused to Venus in *Crrbcs::AtRBCS.* Scale bar is 2 µm*.* **C.** BST4-Venus in a wild type (WT) Chlamydomonas cell. Diagram of BST4 topology is shown on the left-hand side. Scale bar is 1 µm and applies to all images. **D.** PsaF-mNeon (fluorescently tagged component of photosystem I) in *Crrbcs::AtRBCS* Diagram of PsaF topology is shown on the left-hand side. Venus and mNeonGreen fluorescence are shown in green and chlorophyll autofluorescence in magenta. Venus and mNeonGreen are commonly used to tag proteins in Chlamydomonas (Mackinder et al., 2017). Scale bar is 1 µm and applies to all images.


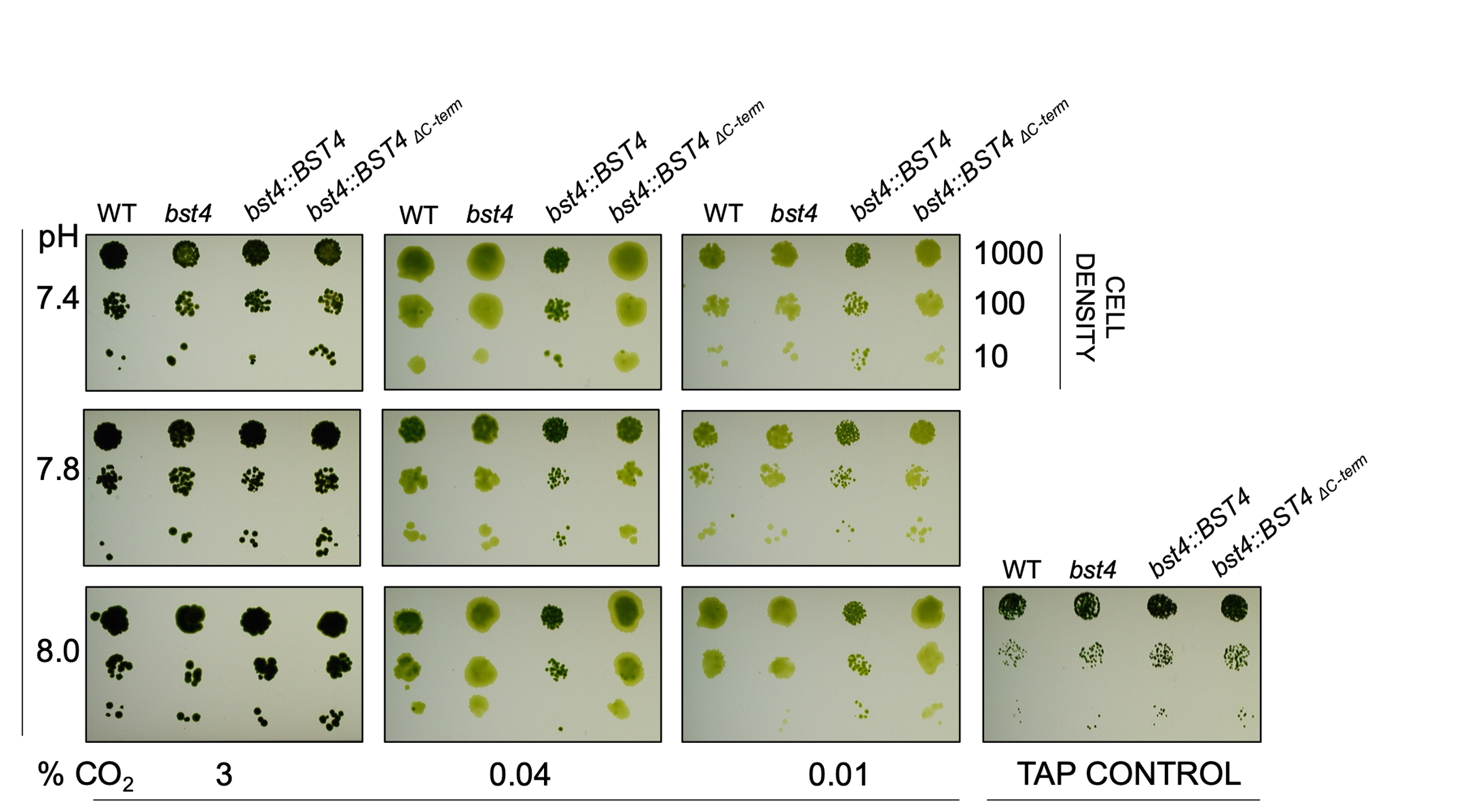


**Supplemental Figure S10. Supporting Figure 6, Spot test of WT, *bst4* and BST4 complemented lines under CCM induced conditions*.*** *Chlamydomonas* strains were grown in serial dilution on agar plates in saturating light (400 μmol m^−2^ s^−1^) under a range of CO_2_ (+/- 2 ppm) and pH conditions (specified) to induce the CO_2_ concentrating mechanism (CCM). All lines grew comparably to wild type (WT) across the conditions used and on the low light Tris-Acetate-Phosphate (TAP) medium control plate.

**Supplemental Figure S11. Supporting Figure 6, Phototaxis and ROS assay. A.** TP (Tris-Phosphate) minimal media liquid cultures of Chlamydomonas cells were uniformly distributed on 0.8% (w/v) agar and subjected to directional light (150 μmol m^−2^ s^−1^). Cell phototaxis was monitored at 0, 1, and 3 h. The assay was performed in the presence of 75 µM reactive oxygen species (ROS) hydrogen peroxide (H_2_O_2_) or 0.3 M ROS quencher N,N'-dimethylthiourea (DMTU). **B.** H_2_O_2_ assay. Chlamydomonas cells were grown in minimal TP liquid media and exposed to 150 µmol photons m^-2^s^-1^. A subset of cells were treated with the quencher DMTU. The concentration of H_2_O_2_ was subsequently quantified using Amplex Red (n=4), and is presented both proportionately to cell density and chlorophyll content. Different letters indicate significance (p<0.05) as determined by a one-way ANOVA and Tukey’s post-hoc test.

**Supplemental Figure S12. Supporting Figure 6 and 8, Chlorophyll fluorescence measurements.** **A.** Y(II) during 5 min illumination and **B.** Y(NO) **C.** Y(NPQ) **D.** 1-qL **E.** qP and **F.** rETRII. Points and lines represent each biological replicate and the mean, respectively (n=3). Shading on graphs indicates when sample is in the dark, unshaded regions indicate application of actinic light. Parameter abbreviations are as follows: Y(II), yield of photosystem II; Y(NO), Yield of Non-regulated Energy Dissipation in PSII; Y(NPQ), Yield of Regulated Non-photochemical Quenching; 1-qL, Fraction of Closed PSII Reaction Centers; qP, Photochemical Quenching Coefficient; rETRII, Relative Electron Transport Rate of PSII.


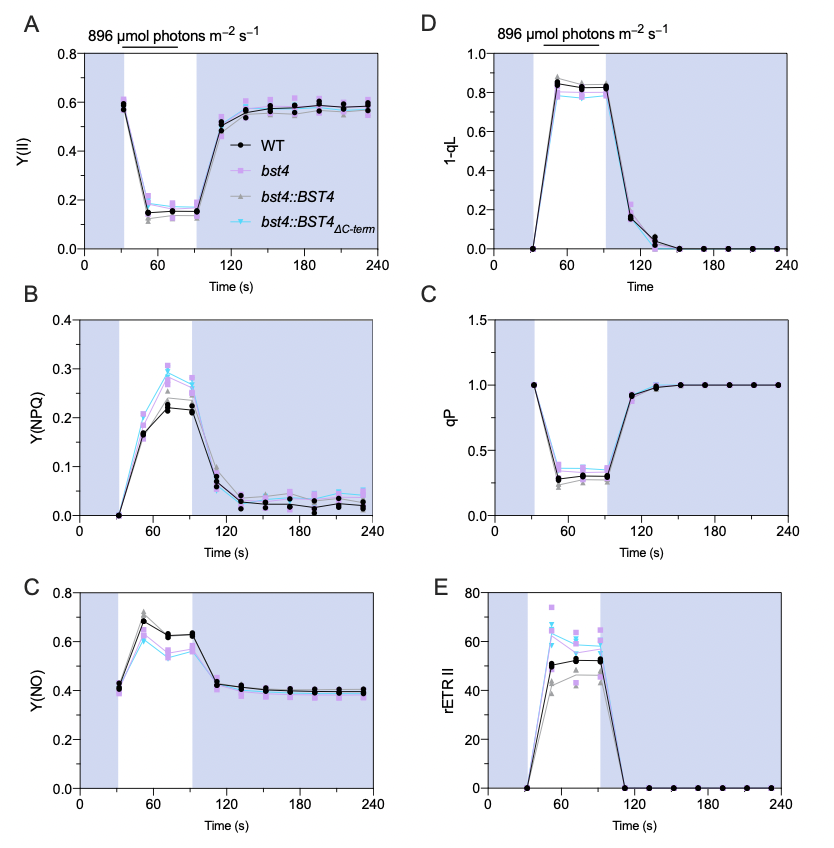


**Supplemental Figure S13. Supporting Figure 6 and 8, Chlorophyll fluorescence measurements.** **A.** Y(II) during 1 min illumination and **B.** Y(NO) **C.** Y(NPQ) **D.** 1-qL **E.** qP and **F.** rETRII. Points and lines represent each biological replicate and the mean, respectively (n=3). Shading on graphs indicates when sample is in the dark, unshaded regions indicate application of actinic light. Parameter abbreviations are as follows: Y(II), yield of photosystem II; Y(NO), Yield of Non-regulated Energy Dissipation in PSII; Y(NPQ), Yield of Regulated Non-photochemical Quenching; 1-qL, Fraction of Closed PSII Reaction Centers; qP, Photochemical Quenching Coefficient; rETRII, Relative Electron Transport Rate of PSII.

**Supplemental Figure S14. Supporting Figure 6, Chlamydomonas bst4 mutant has an enhanced NPQ and proton conductance under high light and limiting Ci conditions.** Wild type (WT) and mutants were grown on Tris-Acetate-Phosphate (TAP) medium at 20 µmol photons m^−2^ s^−1^, resuspended in TP at 30 µg Chl ml^-1^ and exposed for 3 h to light at 150 µmol photons m^−2^ s^−1^. The cells were dark adapted for 1 h before the measurements. **A.** Maximum quantum yield of photosystem II. Individual data points and the mean ± SEM are shown (n=3 replicates) **B.** Dynamics of photosynthesis on transition from dark to high light. Kinetics for induction of chlorophyll fluorescence were recorded during 17 min of illumination at 1500 µmol photons m^−2^ s^−1^ followed by 5 min in darkness. Error bars are ± SEM (n=3). Non-photochemical quenching (NPQ) and **C.** Photosystem II quantum yield (Y(II)). **D to F.** ECS decay kinetics were performed on cells pre-exposed for 10 min to high light and the **D.** total proton motif force (PMF), **E.** g_H_^+^, and **F.** total H^+^ flux (v_H_^+^) were determined as described in Methods. Data are the means ± SEM (n=3). **G.** Immunoblot of NPQ protein LHCSR3 in each genotype compared to tubulin after exposure of cells to 3 h 150 µmol photons m^−2^ s^−1^ in TP. **H.** Fluorescence of LHCSR3 protein band normalized to α-tubulin. Data are the means ± SEM (n=2-3). Different letters or “*” indicate statistically significant difference among the genotypes (one-way ANOVA test, followed by Tukey’s post hoc test, P < 0.05) for all graphs. **I.** Representative Electrochromic shift (ECS) curves used to determine PMF values in D. **J.** Representative ECS decay curves. To determine the g_H_^+^ parameter in E., ECS kinetics were recorded during 600 ms dark intervals. The ECS decay of the first 100 ms was fitted to calculate gH+ (s^-1^) =1/time. We note some usual ECS traces of the slower phases after the initial decay, which prevented us from making any conclusion on the partitioning of the pmf.

**Supplemental Figure S15. Supporting Figure 7, Spot test of BST4 strains from Fig. 7 including CRISPR-generated. A. and B.** represent two out of four biological replicates of all seven strains together. A. represents three spot tests that showed a growth defect for *bst4-2* and *-3* under fast fluctuating light and B. represents the replicate where a growth difference was not apparent. Chlamydomonas strains were grown in serial dilution on agar plates under air CO_2_ levels and indicated light regimes. Images from continuous light and fluctuating light (FL) conditions were from day 6 and 13, respectively. Images for 600 µmol photons m^−2^ s^−1^ FL conditions and Tris-Acetate-Phosphate (TAP) were taken on day 19 and 9, respectively. Both wild type (WT) strains are cc-4533, but the one adjacent to *bst4-2* and *-3* is from the same lab and at the time where the CRISPR mutants were generated.

**Supplemental Figure S16. Generation and validation of *bst4* CRISPR knock out lines. A.** Schematic of paromycin cassette insertion into exon 2 of BST4. Primers used to validate insertion in two mutants, B4 and B6 are shown. **B.** PCR to confirm cassette insertion to exon 2. N.t is no template control. **C.** Immunoblot to confirm the absence of BST4 protein. Histone was included as a loading control. WT = wild type.

**Supplemental Figure S17. Supporting Figure 8, Chlorophyll fluorescence measurements.** **A.** Yield of photosystem II photochemistry ,Y(II), during 5 min illumination and **B.** One minute illumination. Points and lines represent each biological replicate and the mean, respectively (n=3). **C.** and **D.** Raw fluorescence curves for A. and B., respectively. Lines represent the mean of each biological replicate (n=3). Curves are normalized to *F_m_*. Genotypes are artificially spaced by 5 s for clarity. Shading on graphs indicates when sample is in the dark, unshaded regions indicate application of actinic light. **E.** *F_v_/F_m_* measurements ±SEM (n=3) **F.** Light curve for WT in the presence of bicarbonate. Points represent the mean of three technical replicates ±SEM.


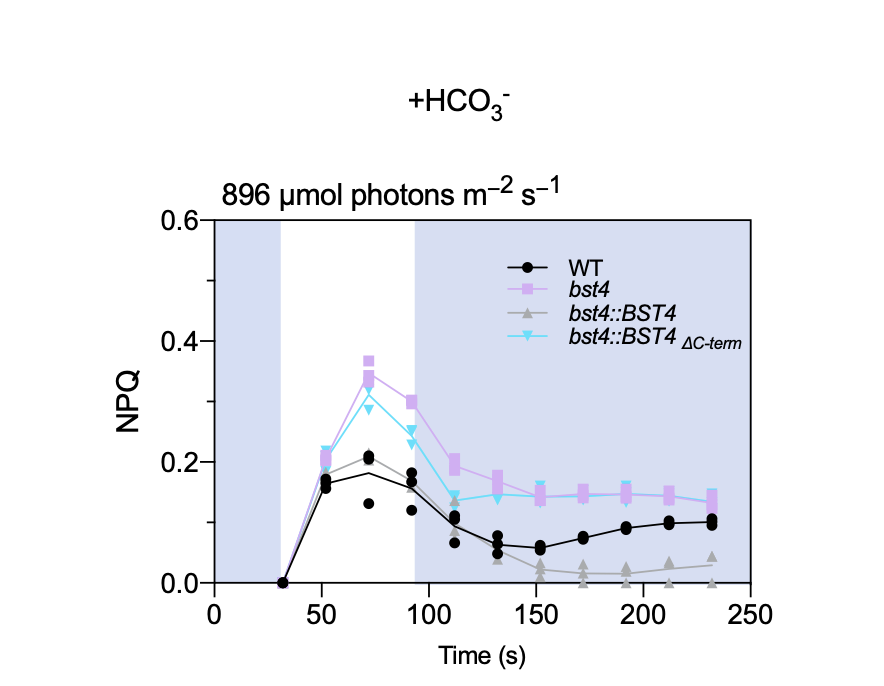


**Supplemental Figure S18. Supporting Figure 8, NPQ with supplemented bicarbonate.** Wild type (WT) and mutants were grown in HS medium at 80 µmol photons m^−2^ s^−1^ and measured at 10 µg Chl ml^-1^. Cells were supplemented with 500 µM HCO_3_^-^and then dark adapted for 5 min before the measurements. Dynamics of Non-photochemical photochemical quenching (NPQ) on transition from dark to high light. Kinetics for induction of chlorophyll fluorescence were recorded during 1 min of illumination at 896 µmol photons m^−2^ s^−1^ followed by 5 min in darkness (shaded region). Points and lines represent each biological replicate and the mean, respectively (n=3).

**Supplemental Figure S19. Supporting Figure 8, Chlorophyll fluorescence measurements of bst4 CLiP and CRISPR mutants.** Strains were grown in modified high salt (HS) medium at 50 µmol photons m^−2^ s^−1^ and measured at 10 µg Chl ml^-1^. **A.** NPQ induction during five minutes of illumination to 540 µmol photons m^−2^ s^−1^. Points and lines represent each biological replicate and the mean, respectively (n=3). **B.** *F_v_/F_m_* of each strain. Asterix indicates statistically significant difference among the genotypes (one-way ANOVA test, followed by Tukey’s post hoc test, P < 0.05). **C.** Normalized raw fluorescence trace, each strain offset by five seconds for clarity. Line represents the mean of three biological replicates. Shading on graphs indicates when sample is in the dark, unshaded regions indicate application of actinic light. WT = wild type.

**Supplemental Figure S20. Supporting Figure 8, The effect of nigericin on NPQ. A. and B.** Wild type (WT) and *bst4* mutant were grown in modified high salt (HS) medium at 80 µmol photons m^−2^ s^−1^ and measured at 10 µg Chl ml^-1^. Cells were supplemented with 10 µM Nigericin after 40 s of illumination. Dynamics of Non-photochemical photochemical quenching (NPQ) were measured on transition from dark to high light. Kinetics for induction of chlorophyll fluorescence were recorded during five minutes of illumination at 896 µmol photons m^−2^ s^−1^ followed by darkness. Points and lines represent each biological replicate and the mean, respectively (n=3). **C.** and **D.** Raw fluorescent traces for A. and B., respectively. Traces are normalized to *F_m_* and represent the mean of three biological replicates.

**Supplemental Figure S21. Supporting Figure 8, Electrochromic shift** (**ECS) traces across four biological repeats.** **A.** Cells resuspended in modified high salt (HS) media at 150 µg Chl ml^-1^ were dark-adapted for 1 min and then illuminated for 1 min with 890 µmol photons m^−2^ s^−1^ after which the light was switched off to record ECS in darkness. We were unable to calculate the partitioning of the proton motive force (PMF) due to non-canonical ECS slow kinetics. **B.** Normalized ECS decay curves. ECS decay of the first 100 ms was fitted to calculate g_H_^+^ (s^-1^) =1/time constant for decay. Data are the means of n=3 technical replicates. WT = wild type.


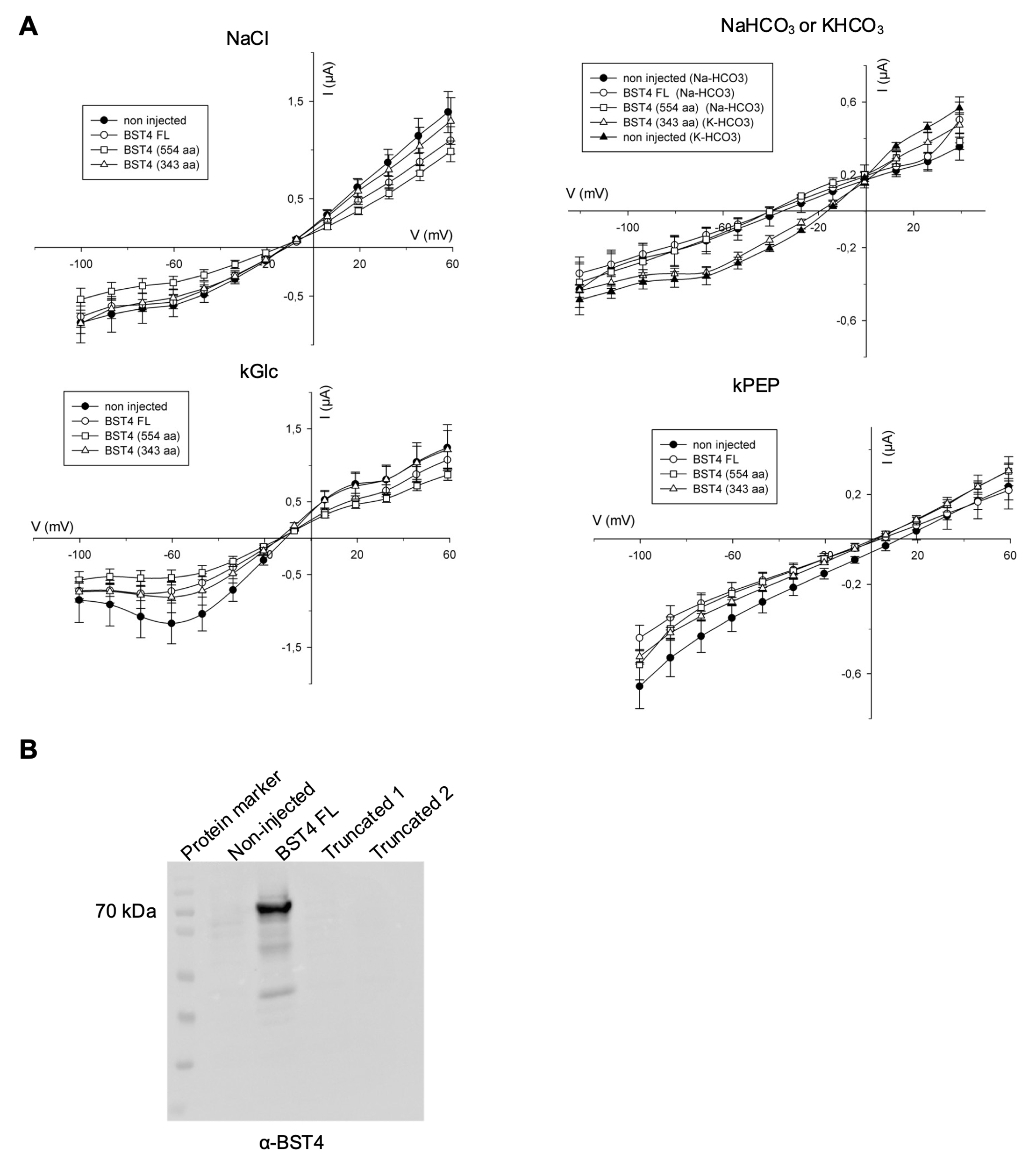


**Supplemental Figure S22. No currents were detected for BST4 with any anions tested in Xenopus oocytes A.** Steady state currents analysis of oocytes injected with BST4, full length or truncated, compared to non-injected oocytes. The voltage steps start from 60 mV to -100 mV (Cl^-^, PEP^-^, and Gluconate conditions) 40 mV to -120 mV (HCO_3_^-^conditions, right panel), holding voltage is -20 mV. Recordings were performed on n>4 oocytes. Error bars are ± SEM. There is no differences between the currents recorded in non-injected oocytes and expressing the protein. **B.** Western Blot analysis of oocytes. Lane 1 is the marker, lane 2 are non-injected oocytes, lane 3 is the full-length (FL) protein (about 66 kDa), lanes 3 and 4 are the truncated proteins. We can only detect full length BST4 as the antibody is directed against the C-terminal part of the protein that is removed in the two truncated versions of the channel.

**Rationale for testing kGlc and kPEP:** BST4 might be permeable to organic ions, similarly to *Hs*Best1, which has been shown to be permeable to γ-aminobutyric acid (GABA) (Lee et al., 2010) and glutamate (Woo et al., 2012), as well as Cl^-^ and HCO_3_^-^ anions. Fei et al. (2022) proposed a model whereby ribulose bisphosphate (RuBP) acts as proton carrier to increase H^+^ concentration in the pyrenoid tubules. It is possible that BST4 is the channel that facilitates RuBP translocation in the tubules in this model. To test this hypothesis, we used small molecule analogues K-PEP and K-Gluconate but no currents were detected for these either.

>Brassica_rapa_(PAC:30641593)

---------------------------------------------------------------------------------------------------------------------------------------------------RTLYTHEKWVEHRSSLRHVHHLFSSFSSRVILSLIPPVFFFTSVAIFIASYNSAVAL-------------DWLPSVFPILR---SSSLPYQLTAPALALLLVFRTEASYSRYEEGRKAWVGIIAGT--DDLARQVICSVDGSG-----------------DELVIK-----DLLLRYV----AAFPVALKCHVTYGSD-----VARDLRN-----LIEGDDLSLI--IESKHR-------------PRCVIEFISQSLQL----LKLDDT--------------KRDLLESKMLHLHEGIGVCEQLMGIPIPLAYTRL-TSRFLVFWHLTL-------PIILWDEC-----HWIVVPATFISAASLFCIEEVGVLIEEPFPM----LALDELCDL-VHSNIQEAVKSES-------------------------------------------------------------------------------------------------------------------------------------------------------------------------------------------------------------------------------------------------------------------------------------------------------------------------------------------------------------------------------------------------------------------------------------------

>Q9M2D2_-_VCCN1_Athaliana

MY------------------QSMNLSVSSNFTHRSLLES------------RFPI-----FSTGFR-----KSVNLKPPRVSSGPE---SNDSGH---------------ETLTDKLIHLLRAVP--------DWADEIKERGMQQKRSLYTHEKWVEHRSSLRHVRHLLSSFSSRVILSLIPPVFFFTSVAVVIASYNSAVAL-------------DWLPGIFPILR---SSSLPYQLTAPALALLLVFRTEASYSRYEEGRKAWVGIIAGT--NDLARQVICSVDSSG-----------------DELIIK-----DLLLRYI----AAFPVALKCHVIYGSD-----IARDLRN-----LIEADDLSLI--LQAKHR-------------PRCVIEFISQSIQL----LKLDDA--------------KRDLLESKMLHLHEGIGVCEQLMGIPIPLSYTRL-TSRFLVFWHLTL-------PIILWDEC-----HWIVVPATFISAASLFCIEEVGVLIEEPFPM----LALDELCDL-VHSNIQEAVKSEKVIRNRIIAKIKLHEFK------HSS--------------------------NGRHRS----------------------------------------------------------------------------------------------------------------------------------------------------------------------------------------------------------------------------------------------------------------------------------------------------------------------------------------------------------------------------------

>Nicotiana_tomentosiformis_(XP_009629643.1)

M--------------------------TNSRTLFSIQSPTNASFSSHF-TLKTPSK--LQQQSFPSKL-NFKKLRFSTFKVRCCPQ----QTPQN---------------QNPTSALISILRIIP--------DWADRIQEEGMKKKRSLYTHESWMQHRSSLRHVRHLFSSLNSRVILSLVPPVIAFTSVAVVIASYNSAVSM-------------HWLPELFPVLR---ASPLPYQLTAPALALLLVFRTEASYSRFETGKKAWTKVIAGT--NDFARQVIACVDKS-------------------DAVLK-----AALLQYI----MAFPVALKCHITYGSD-----IASDLKN-----LLEADDLAVV--LSSKHR-------------PRCIIGFISQCLQS----LHLEGT--------------KLTQLESKISCFHEGIGVCEQLAGIPIPLSYTRL-TSRFLVLWHLTL-------PIILWDDC-----HWIVVPATFISAASLFCIEEVGVLIEEPFPM----LALDELCQL-VHDNIQESMANEKKIQERLSAKRKRRFSE------HSQ--------------------------NGWPTS----------------------------------------------------------------------------------------------------------------------------------------------------------------------------------------------------------------------------------------------------------------------------------------------------------------------------------------------------------------------------------

**Supplemental Figure S23. Sequences used to generate Multiple Alignment using Fast Fourier Transform (MAFFT) alignments used to generate the phylogenetic trees included in this manuscript Part 1 of 6.** FASTA files of MAFFT alignments of full length BST4.

>XP_028961536_Malus_domestica

MLLPSSLSLQTLAPPNADTIQKPHQTLPQNLTLFQLVHP------QHFPTLQFPN-----LPSGPKTL-KFKLLCSQSPNPNPSP-------PSS---------------SSPVQTLISILRIIP--------DWSDRTQERGMRQHRTLYDHEKWMHHRSSYRHLRHLLSSLSSRVILSLIPPVIAFTLVAVVIASYNTAVAL-------------DLLPGIFPLLR---SSSLPYQLTAPALALLLVFRTEASYSRFEEGRKAWTEVIAGA--NDFARQIISSVETSG------------------DAQLK-----KALLQYI----VAFPVALKCHVIYGSD-----IARDLQN-----LLEVDDLLVV--LNSKHR-------------PGCIIQFISRSLQL----LKLEES--------------RRIMLQSKISCFHEGIGICEQLIGTPIPLSYTRL-TSRFLVLWHLTL-------PIILWDDC-----HWIVVPATFISAASLFCIEQVGVLIEEPFPM----LALDDLCNS-VRNNVQEALASEKLIRARLAAKGRIQSEQ------QFQ--------------------------NGQPRP----------------------------------------------------------------------------------------------------------------------------------------------------------------------------------------------------------------------------------------------------------------------------------------------------------------------------------------------------------------------------------

>Chlamydomonas_eustigma_(GAX83184.1)

M--------------------------------------------------------------------LVHRVHTRSLGNRNQCGRK----------------------LHRVSTFVVKTPTEKPVVA----DYVLPRSEEARRYFRTVYDFPQWQKHRSPTRLIDRLLQIPRSHVLQNILPSIAWCSSVAGLLTLYMQAYDA-------------HILPDGFPSFATNNACTSFVNTTTVALSLLLVFRTNVSYGRWDEARKMKGLLVNRS--RDLMRQVCAMVPEE-------------------DVATK-----AMMAKWT----AAFCRVLRIHFQPEVS-----LEDEMKG-----LLSPEELEWL--IESKHR-------------PCSVIHMLSQIIYD----SQISAI--------------CQAQMCNNLTAFEDVLGGCERLLRAPIPVSYTRH-TARFLFTWLTLL-------PFALYNSC-----GVWTLPVVAGVSAVLCGIEEIGVQIEEPFGI----LPLEAICGR-IQADVMATLKEDAKTRNLRNKVLLIRGPE------HWAMV------------------------NGHSNK----------------------------------------------------------------------------------------------------------------------------------------------------------------------------------------------------------------------------------------------------------------------------------------------------------------------------------------------------------------------------------

>Cre16.g662600.t1.2_(BST1)

M---------------------------------QMQA-----------NRSSLRASPVRGLGARPLLRALPAGRVARLNVSAQA--KDPNAPIQ---------------SNPLGTLSSQSGQVA----------TLPRSEEARKYFRTVYDFPQWQKHRSSYRFAERLFQLSQSHILQNALPAISWVTLVATLVASYGYSYDQ-------------HMLPDVFPSISPNASCTAFISNTSVALSLLLVFRTNSSYGRWDEARKMWGGLLNRS--RDIMRQGATCFPDD-------------------QVEAK-----KALARWT----VAFSRALRIHFQPEVT-----IESELQN-----ILTPAELQML--AKSQHR-------------PVRAIHAISQIIQS----VPMSSI--------------HQQQMSNNLTFFHDVLGGCERLLRAPIPVSYTRH-TARFLFAWLTLL-------PFALYPTT-----GWGVVPVCTGIAAVLCGIEEIGVQCEEPFGI----LPLDVICNR-IQADVMATLKDDADTKTILAEAGLISLIPS----ATSATPVASA----EPVLVSARPSAAPAPNNGLQVRVAM--------------------------------------------------------------------------GG---------------------ERK*--------------------------------------------------------------------------------------------------------------------------------------------------------------------------------------------------------------------------------------------------------------------------

**Supplemental Figure S24. Sequences used to generate Multiple Alignment using Fast Fourier Transform (MAFFT) alignments used to generate the phylogenetic trees included in this manuscript part 2 of 6.** FASTA files of MAFFT alignments of **f**ull length BST4.

>Cre16.g663400.t2.1_(BST2)

M---------------------------------QC-------------LSSRPVAMGRAGSSALPRL-PLRAGRVCHLGVRCQAANKDPNAPIQ---------------SNPLGSFSSQLQNQP----------TLPRSEEARKYFRTVYDFPQWQTHRNQYRLMKRLFSIPQSHVIQNALPSIMWVAFTSTCVAAYMYGYDQ-------------HMLPEGFPTLAPNAACSAFISNTSVALSLLLVFRTNSSYGRWDEARKMWGGLLNRS--RDIMRQGATCFPDD-------------------QVEAK-----KALARWV----VAFSRALRIHFQPEVT-----IESELKN-----ILTPAELQML--AKSQHR-------------PVRAIHAISQIIQS----VPMSSI--------------HQQQMSNNLTFFHDVLGGCERLLRAPIPVSYTRH-TARFLFAWLTLL-------PFALYGSC-----GVSVIPVCSGIAAVLCGIEEIGVQCEEPFGI----LPLDVICNR-IQADVMATLKDDADTKTILAEAGLISLRA------NSAMAVENALPDLDSINAAAPNGNGSHNGNGAAV-------------------------------------------------------------------------------------------------------PVS---------------------------VSAGA--------------------------------------------------------------------------------------------------------------------------------------------------------------------------------------------SGNGMNV---------------------------RISPR*-----

>Cre16.g663450.t1.2_(BST3)

M---------------------------------QVSK-----------VPSS------ASARCLPRL-PVRTSRVCQLSVRCQAANKDPNAPIQ---------------SNPLGSFSSQNSSGAVV--------TAPRNEDARKYFRTVYDFPQWQKHRSQSRLVRRLFTIPQSHVIQNALPSIMWVTFTSTCVAAYMYGYDL-------------HILPEGFPTLAPNAACSAFISNTSVALSLLLVFRTNSSYGRWDEARKMWGGLLNRS--RDIMRQGATCFPDD-------------------QVEAK-----KALARWT----VAFARALRIHFQPEVT-----IESELQN-----ILTPAELQML--AKSQHR-------------PVRAIHAISQIIQS----VRMSSI--------------HQQQMSNNLTFFHDVLGGCERLLRAPIPVSYTRH-TARFLFAWLTLL-------PFALYGSC-----GVSVIPVCTGIAAVLCGIEEIGVQCEEPFGI----LPLDVICNR-IQADVMATLKDDADTKTVLAEAGLISLIP------SMSLPPTEHASPSDPVTAAAAAALAAANGNGAASHSNG--------------------------------------------------------------------------NG---------------------SKPVSTQVPP-----------------PVLAPVTVTS--------------------------------------------------------------------------------------------------------------------------------------------------------------------------------------------SSGSMNV---------------------------RISPR*-----

>Chlorella_sorokiniana_(PRW33726.1)

M-----------------------STAMLAGSRIQLQQPAG--------LGGSRLQ--RAAAPVAAAA-RLGSVRPAGLQARSTAARRADRSALR---------------VSATASPEAAPVKLS----------GDDLKEANRKHMRSVFDFDLWKKHRSSSRYLRHIVGLGESRIVSGLMAPLTYVMTLSLAVACYNAAAEA-------------GYLP-VFPELKL--ATNAPFGLTSFALSLLLVFRTNSSYGRWDEARKMWGLIVNRS--RDFIRQGLGYIPPE-------------------QEELQ-----KMLVRWT----VAYSRSLMCHLRPGED-----LRVELKD-----TLKPEELEAL--LASTHR-------------PNYVVQVLTAIIKT----AQLPAAVTNNRDSTGCVPAGAAYRMDENLTVFADVTGGCERILRTPIPLSYTRH-TSRFMMIWLTLL-------PFTLWDSC-----HWAMLPIAGIVSFLLLGIEEIGVQIEEPFTI----LPLEVISRT-IEGNVWELYRMHSGEA---LEKEQAELAN------GQDVQVLNA----QDLVALMAPSAVGNTANGTSRKSLV------------------VNYGL--------------------------------------------------------------------------------------------------------------------------------------------------------------------------------------------------------------------------------------------------------------------------------------------------------------------------------------------------------

**Supplemental Figure S25. Sequences used to generate Multiple Alignment using Fast Fourier Transform (MAFFT) alignments used to generate the phylogenetic trees included in this manuscript part 3 of 6.** FASTA files of MAFFT alignments of full length BST4.

>Cre06.g261750.t1.2_(BST4)

M-----------------------QC--------QLKH----------------------------GA-RPQSQRPNWLPARAATLRPAVQHGVR---------------RGLTLGVKAAAAPLE--------DKKMPADMTTRQYRRVVYDFALWAKHRDVNRYLYNLRTIPGSRIIRQLSQPMGVVLAWAALFGFYETCLEA-------------GVLPSYLPKMTL--MSAEPQGLTSFALSLLLVFRTNSSYGRFDEARKIWGGILNRA--RNIANQAVTFIPAE-------------------DQAGR-----EAVGKWT----VGFTRALQAHLQEDID-----LRKELEKA--TPRWSKEEIDML--VNAQHR-------------PIKAISVLSELTRQ----LSITQF--------------QALQMQENCTFFYDALGGCERLLRTPIPVSYTRH-TARFLTIWLAML-------PLGLWERY-----HWSMLPVIALIGFLLLGIDEIGISIEEPFGI----LPLDAICGR-AQTDVNSLLKEDPAVMKYVDDVRSGRVKS------PPPLPPAPA----APAAA-------AAAAAAAARSVSP----QPDVAKTLGSLFTNVRAGVGAVAPGAPLMPQAPVRSPSPTRSVSP--------SFPRASAGTGMPPPVGMNG----------ATPRVAAAPPTPPPVSRPAA------------------PAAAPAAGSGFTMPNFSASLSGLTGGAAAAAKSAADAASSKLTKMADSMSSGAAAPAPPAAPAR---------------------PSTSPRPSASSPISSSADADRSDSSRR---PVNWRDELQSLKATRE----------------PNGNGNGSGVAPAA--GRADADEEALRRFGNLAGRSR--------------SGNG---------------GGGSSDTELSEANRPRTRPDWRNQL*

>XP_002945810.1_Volvox_carteri_f._nagariensis

M-----------------------QS--------QLQP----------------------------RL-QLQGTRLNWLPQRSCVQRRSLRVDAT---------------SG------AAPPPPA--------GKELSNDMVTRQYRRTVYDFSLWAKHRDVNRYLYNLKTIPGSRIIRTLGQPMGIVLAWAAMFGFYETCLES-------------GVLPSYFPKLTL--MSAEPQGLTSFALSLLLVFRTNSSYGRFDEARKIWGGILNRA--RNIANQAVTFIPAE-------------------DVAGR-----EAVGKWA----VGFCRALQAHLQEDAN-----LREELQKA--QPRWSREEIDML--CSAQHSWQQLQSCVNAFW-PIKAISMLSELTRQ----LPISQF--------------QALQMQENVTFFYDALGGCERLLRTPIPVSYTRI--------------------------------------------------------------------LPLDAICTR-AQTDVVSLLKDDPAVVKYISDVRQGRIAP------PTEPPVAGA----APVAAAPPPPPPASAGGGISRSGSPTAQQQPDVMKTVTSMLHNVKAGIGAVAPAPPRPPSPQPRARSP-RAASPGGPS----PFPRASAGTG--------G----------AAAAVPSPPPIKPLTSSSSSSSGAVSKDSNNSTATAKKPASAPAASSA----GFSMGFSGLADGAAAAAKSAS-AAAAKFSKIADSVVAG--TPAAPASEAKRETAA--AAAMQAQPRN---TPSSSSSTPSAAPANGSSDDDRSSSGRRTAAAVNWREELAALRAGREDAEEPASASASYDREFPSSSWSFSSASSAAVVQSGDAEDEARRRFGGLAGRGARSDTTTSAAAVMRGNGNGLSENGYGNGYGNDN-GNGNGNTVEARGARPRTRPDWRNQL-

>Chrysochromulina_sp._(KOO32217.1)

M-----------------------REHPLSYEEYMRQRS----------AGRDPLA--EAVQGQSASM-ERGVVPPPPVKATPERTEAAFTPPAEFDFFQDVFKPTVESVVKAVVSPGAQQDSDESYMRVPWWEQGSTYSEDQRKDRRTVFMHDDWKRHRSSERFFRNIKTWPSSGINQALRKELTFVTSVSVFVVLANMLLYQYQDFGGVVHPGPLSFLDGPIKSLS---LPALPFSMASPVLSLLLVFRTNTAYFRWNEARTLWGGLINNC--RNIVRQTTTMFPNDA-----------------YHNALK-----KRLATET----ATFIKSLRNFLRGPEDDAT--LRKELYAYVNQGLMTSAQAEAT--LAAKNR-------------PMFALAAMSATLRK----ANIDEM--------------YISRMDSTISVLVDLTGANERIFKSPIPLVYTRL-TARFLSVFLTLL-------PLAMWAALGESWNHWATIPATFILSVFLFGVEEVGIQIEEPFSI----LPLEAMCNGAIEAVQLEMLAAE-----------------------QSQVFEAAG----DAVAVA--------------------------------------------------------------------------------------------------------------------------------------------------------------------------------------------------------------------------------------------------------------------------------------------------------------------------------------------------------------------------------------------------

**Supplemental Figure S26. Sequences used to generate Multiple Alignment using Fast Fourier Transform (MAFFT) alignments used to generate the phylogenetic trees included in this manuscript part 4 of 6.** FASTA files of MAFFT alignments of full length BST4.

>Emiliania_huxleyi_(XP_005770556.1)

-----------------------------------------------------------------------------------------------------------------------------------------EFKEAGREFRQDVYSYNDWRWHRESGHIASAISSVFTSGVGKAMWRETFFVIATAAAVYLYNIGVPVLAAKTAASL----PIVAALLGRLPLLHLSLLPLTLSSPALFLLLVFRTNNSYDRWWEARKVWGGVINAS--RDLARQALALVR---------------------DAELK-----KLMVSQI----ASYARVLKYHLGPPTPEARDLLRNELVD----NRLPADQVRVI--MEAKHK-------------PMALLGLVSASLHDSGR-TGLDTV--------------QASKLDQTLSLLTDYLGKCERIVKTPLPLVYTRH-TARFLSWWLLFL-------PVCLYNQLRA---NWMIVPVSGLIGFFLVGIEDLGNQIEEPFSI----LPLTAMGTG-IQQSIFEAL-----------------------------------------------------------------------------------------------------------------------------------------------------------------------------------------------------------------------------------------------------------------------------------------------------------------------------------------------------------------------------------------------------------------------------------------------

>Phaeodactylum_tricornutum_(XP_002180738.1)

MM--------------------------------------------------------RNFASVLLLL-SSGAAAFAPVQHNGVRTIATPSTPLY---------------GNTKQPPALP--PIK----------DISYGEESRKYRRTVYSHDDWVKHRSSDRFLRNLLAIGSSGVYKSLAKEVLATTGVATFIVLYNCLVGGYTDLEGIKHS---ALIESVWAPLMA--LPLAPFTLSSPSLGLLLVFRTNTSYQRWDEARKNWGMNINHT--RDLVRMGTSFYDNAA-----------------VSSEQRAKDL-KALSLAT----WSFVRAMKRHLSPESEDEQD-FRRELFE-----RLPAPQAQAI--IDAAHR-------------PNRALFDLSVAIEN----LPMHFL--------------RKNQVHQAVTIFEDNLGSSERLLTSPVPLFYSRH-TARFLSFWLLLL-------PFALWDPFAGTWNHVGMIPATAVISIFLFGIEELATQMEEPFTI----LPMQAFCDK-IGNWCNEIVSWQAGDNGMAVNMPSMISPEGLPELKEPAPVPAMA----VASVAAAMPVMANGDINGDTTGITM---DQP-------------------------------------------------HNAIP-------------------------------------------------------------------------------------------------------------------------------------------------------------------------------------------------------------------------------------------------------------------------------------------------------------------

>Thalassiosira_pseudonana_(XP_002289965.1)

M-----------------------------------------------------------------------------------------------------------------GPPIDPSVPVT-----------DQVGEGSRKYRRTVYTHDDWVRHRSPDRFGNNLSTLFNSGIYKQVANEVFATTAVATFVFLWNMIAGGYTDLAGVQHG---PIIDSPLAQMVG--LPMTAFTILTPSLGLLLVFRTNTSYGRWDEARKMWGLNINHT--RDLNRMATAWYGNEGNMDSVAFMGGDIPYSQPIDPVQRAYDL-GQVSLFT----WAFVRSMKRHLSPPEEDEED-FKAELRA-----RLTPEQAENI--INAAHR-------------PNRALFDLSVAIEN----LPMHFL--------------RKNAINTNLSIFEDTLGGCERLLSSPVPLFYSRH-TARFLSTWLLLL-------PFGLYEQFKDSWNHIAMIPATAFISVCLFGIEELATQLEEPFTI----LPMQGFCDK-IGGWCDEIVSW-AGQGQQEYTEENAMSNE------QEMTY-----------------------------------------------------------------------------------------------------------------------------------------------------------------------------------------------------------------------------------------------------------------------------------WR---------------------------------------------------------------------------------------------------------------------------

**Supplemental Figure S27. Sequences used to generate Multiple Alignment using Fast Fourier Transform (MAFFT) alignments used to generate the phylogenetic trees included in this manuscript part 5 of 6.** FASTA files of MAFFT alignments of full length BST4.

>WP_049046555.1_Kiebsiella_aerogenes

------------------------------------------------------------------------------------------------------------------------------------------------------------MIIRPEQHWFFRLFDWHGSVLSKIVFRLLLNVLMSVIAIISYQWYEQL-------------GI-----------HLTVAPFSLLGIAIAIFLGFRNSASYSRFVEARNLWGTVLIAE--RTLVRQLKNILPD--------------------DEETH-----KTLVSYL----VAFSWSLKHQLRK-TD-----PAVDLYR-----LLPKEKVAEI--LASSMP-------------TNRILLLIGNELGRLREQGKLSDI--------------TYGLMDNKLDELAHVLGGCERLASTPVPFAYTLI-LQRTVYLFCTLL-------PFALVGDL-----HYMTPFVSVFISYTFLSWDSLAEELEDPFGTSANDLPLNAMCNT-IERNLMDMTGQHPLPEKMQPDRYYNLT-----------------------------------------------------------------------------------------------------------------------------------------------------------------------------------------------------------------------------------------------------------------------------------------------------------------------------------------------------------------------------------------------------------------------------

>XP_011543531.1_Homo_sapiens

M----------------------------------------------------------------------------------------------------------------------------------------------------TITYTSQVANARLGSFSRLLLCWRGSIYKLLYGEFLIFLLCYYIIRFIYRLALTEEQQL--------------MFEKLTL--YCDSYIQL--IPISFVLGFYVTLVVTRW------WNQYENLPWPDRLMSLVSGFVEGK-------------------DEQGRL--LRRTLIRYANLGNVLILRSVSTAVYKRFP-----SAQHLVQ---AGFMTPAEHKQLEKLSLPHNM---------FWVPWVWFANLSMKAWLG---GRIRDPI-------------LLQSLLNEMNTLRTQCGHLYAYDWISIPLVYTQVVTVAVYSFFLTCLVGRQFLNPAKAYPGHEL---DLVVPVFTFLQFFFYVGWLKVAEQLINPFGEDDDDFETNWIVDRNLQVSLLAVDEMHQDLPRMEPDMYWNKPEP------QPPYTAASA----QFRRASFMGSTFNISLNKEEMEFQPNQEDEED-----------AHAGIIGRFLGLQSHDHHPPRANSRTKLLWPKRESLLHEGLPKNHKAAKQ----NVRGQEDNKAWKLKAVDAFKSAPLYQRPGYYSAPQ-----------TPLSPTPMFFPLEPSA---PSKLHSVTGI-DTKDKSLKTVSSGAKKSFELLSES--DGALMEHPEVSQVRRKTVEFNLTDMPEIPENHLKEPLEQSPTNIHTTLKDHMDPYWALENR----------SVLHLNQGHCIALCPTPASLALSLPFLHNFLGFHHCQSTLDLRPALAWGIYLATFTGILGKC---------------SGPFLTSPWY---HPEDFLGPGEGR--------------------

**Supplemental Figure S28. Sequences used to generate Multiple Alignment using Fast Fourier Transform (MAFFT) alignments used to generate the phylogenetic trees included in this manuscript part 6 of 6.** FASTA files of MAFFT alignments of full length BST4.

>Brassica_rapa_(PAC:30641593)

---------------------------------------------------------------------------------------------------------------------------------------------------RTLYTHEKWVEHRSSLRHVHHLFSSFSSRVILSLIPPVFFFTSVAIFIASYNSAVAL-------------DWLPSVFPILR---SSSLPYQLTAPALALLLVFRTEASYSRYEEGRKAWVGIIAGT--DDLARQVICSVDGSG-----------------DELVIK-----DLLLRYV----AAFPVALKCHVTYGSD-----VARDLRN-----LIEGDDLSLI--IESKHR-------------PRCVIEFISQSLQL----LKLDDT--------------KRDLLESKMLHLHEGIGVCEQLMGIPIPLAYTRL-TSRFLVFWHLTL-------PIILWDEC-----HWIVVPATFISAASLFCIEEVGVLIEEPFPM----LALDELCDL-VHSNIQEAVKSE

>Q9M2D2_-_VCCN1_Athaliana

MY------------------QSMNLSVSSNFTHRSLLES------------RFPI-----FSTGFR-----KSVNLKPPRVSSGPE---SNDSGH---------------ETLTDKLIHLLRAVP--------DWADEIKERGMQQKRSLYTHEKWVEHRSSLRHVRHLLSSFSSRVILSLIPPVFFFTSVAVVIASYNSAVAL-------------DWLPGIFPILR---SSSLPYQLTAPALALLLVFRTEASYSRYEEGRKAWVGIIAGT--NDLARQVICSVDSSG-----------------DELIIK-----DLLLRYI----AAFPVALKCHVIYGSD-----IARDLRN-----LIEADDLSLI--LQAKHR-------------PRCVIEFISQSIQL----LKLDDA--------------KRDLLESKMLHLHEGIGVCEQLMGIPIPLSYTRL-TSRFLVFWHLTL-------PIILWDEC-----HWIVVPATFISAASLFCIEEVGVLIEEPFPM----LALDELCDL-VHSNIQEAVKSE

>Nicotiana_tomentosiformis_(XP_009629643.1)

M--------------------------TNSRTLFSIQSPTNASFSSHF-TLKTPSK--LQQQSFPSKL-NFKKLRFSTFKVRCCPQ----QTPQN---------------QNPTSALISILRIIP--------DWADRIQEEGMKKKRSLYTHESWMQHRSSLRHVRHLFSSLNSRVILSLVPPVIAFTSVAVVIASYNSAVSM-------------HWLPELFPVLR---ASPLPYQLTAPALALLLVFRTEASYSRFETGKKAWTKVIAGT--NDFARQVIACVDKS-------------------DAVLK-----AALLQYI----MAFPVALKCHITYGSD-----IASDLKN-----LLEADDLAVV--LSSKHR-------------PRCIIGFISQCLQS----LHLEGT--------------KLTQLESKISCFHEGIGVCEQLAGIPIPLSYTRL-TSRFLVLWHLTL-------PIILWDDC-----HWIVVPATFISAASLFCIEEVGVLIEEPFPM----LALDELCQL-VHDNIQESMANE

>XP_028961536_Malus_domestica

MLLPSSLSLQTLAPPNADTIQKPHQTLPQNLTLFQLVHP------QHFPTLQFPN-----LPSGPKTL-KFKLLCSQSPNPNPSP-------PSS---------------SSPVQTLISILRIIP--------DWSDRTQERGMRQHRTLYDHEKWMHHRSSYRHLRHLLSSLSSRVILSLIPPVIAFTLVAVVIASYNTAVAL-------------DLLPGIFPLLR---SSSLPYQLTAPALALLLVFRTEASYSRFEEGRKAWTEVIAGA--NDFARQIISSVETSG------------------DAQLK-----KALLQYI----VAFPVALKCHVIYGSD-----IARDLQN-----LLEVDDLLVV--LNSKHR-------------PGCIIQFISRSLQL----LKLEES--------------RRIMLQSKISCFHEGIGICEQLIGTPIPLSYTRL-TSRFLVLWHLTL-------PIILWDDC-----HWIVVPATFISAASLFCIEQVGVLIEEPFPM----LALDDLCNS-VRNNVQEALASE

**Supplemental Figure S29. Sequences used to generate Multiple Alignment using Fast Fourier Transform (MAFFT) alignments used to generate the phylogenetic trees included in this manuscript 1 of 4.** FASTA files of MAFFT alignments of BST4 trimmed after the bestrophin domain BST4 and homologous amino acid sequences.

>Chlamydomonas_eustigma_(GAX83184.1)

M--------------------------------------------------------------------LVHRVHTRSLGNRNQCGRK----------------------LHRVSTFVVKTPTEKPVVA----DYVLPRSEEARRYFRTVYDFPQWQKHRSPTRLIDRLLQIPRSHVLQNILPSIAWCSSVAGLLTLYMQAYDA-------------HILPDGFPSFATNNACTSFVNTTTVALSLLLVFRTNVSYGRWDEARKMKGLLVNRS--RDLMRQVCAMVPEE-------------------DVATK-----AMMAKWT----AAFCRVLRIHFQPEVS-----LEDEMKG-----LLSPEELEWL--IESKHR-------------PCSVIHMLSQIIYD----SQISAI--------------CQAQMCNNLTAFEDVLGGCERLLRAPIPVSYTRH-TARFLFTWLTLL-------PFALYNSC-----GVWTLPVVAGVSAVLCGIEEIGVQIEEPFGI----LPLEAICGR-IQADVMATLKED

>Cre16.g662600.t1.2_(BST1)

M---------------------------------QMQA-----------NRSSLRASPVRGLGARPLLRALPAGRVARLNVSAQA--KDPNAPIQ---------------SNPLGTLSSQSGQVA----------TLPRSEEARKYFRTVYDFPQWQKHRSSYRFAERLFQLSQSHILQNALPAISWVTLVATLVASYGYSYDQ-------------HMLPDVFPSISPNASCTAFISNTSVALSLLLVFRTNSSYGRWDEARKMWGGLLNRS--RDIMRQGATCFPDD-------------------QVEAK-----KALARWT----VAFSRALRIHFQPEVT-----IESELQN-----ILTPAELQML--AKSQHR-------------PVRAIHAISQIIQS----VPMSSI--------------HQQQMSNNLTFFHDVLGGCERLLRAPIPVSYTRH-TARFLFAWLTLL-------PFALYPTT-----GWGVVPVCTGIAAVLCGIEEIGVQCEEPFGI----LPLDVICNR-IQADVMATLKDD

>Cre16.g663400.t2.1_(BST2)

M---------------------------------QC-------------LSSRPVAMGRAGSSALPRL-PLRAGRVCHLGVRCQAANKDPNAPIQ---------------SNPLGSFSSQLQNQP----------TLPRSEEARKYFRTVYDFPQWQTHRNQYRLMKRLFSIPQSHVIQNALPSIMWVAFTSTCVAAYMYGYDQ-------------HMLPEGFPTLAPNAACSAFISNTSVALSLLLVFRTNSSYGRWDEARKMWGGLLNRS--RDIMRQGATCFPDD-------------------QVEAK-----KALARWV----VAFSRALRIHFQPEVT-----IESELKN-----ILTPAELQML--AKSQHR-------------PVRAIHAISQIIQS----VPMSSI--------------HQQQMSNNLTFFHDVLGGCERLLRAPIPVSYTRH-TARFLFAWLTLL-------PFALYGSC-----GVSVIPVCSGIAAVLCGIEEIGVQCEEPFGI----LPLDVICNR-IQADVMATLKDD

>Cre16.g663450.t1.2_(BST3)

M---------------------------------QVSK-----------VPSS------ASARCLPRL-PVRTSRVCQLSVRCQAANKDPNAPIQ---------------SNPLGSFSSQNSSGAVV--------TAPRNEDARKYFRTVYDFPQWQKHRSQSRLVRRLFTIPQSHVIQNALPSIMWVTFTSTCVAAYMYGYDL-------------HILPEGFPTLAPNAACSAFISNTSVALSLLLVFRTNSSYGRWDEARKMWGGLLNRS--RDIMRQGATCFPDD-------------------QVEAK-----KALARWT----VAFARALRIHFQPEVT-----IESELQN-----ILTPAELQML--AKSQHR-------------PVRAIHAISQIIQS----VRMSSI--------------HQQQMSNNLTFFHDVLGGCERLLRAPIPVSYTRH-TARFLFAWLTLL-------PFALYGSC-----GVSVIPVCTGIAAVLCGIEEIGVQCEEPFGI----LPLDVICNR-IQADVMATLKDD

**Supplemental Figure S30. Sequences used to generate Multiple Alignment using Fast Fourier Transform (MAFFT) alignments used to generate the phylogenetic trees included in this manuscript part 2 of 4.** FASTA files of MAFFT alignments of BST4 trimmed after the bestrophin domain BST4 and homologous amino acid sequences.

>Chlorella_sorokiniana_(PRW33726.1)

M-----------------------STAMLAGSRIQLQQPAG--------LGGSRLQ--RAAAPVAAAA-RLGSVRPAGLQARSTAARRADRSALR---------------VSATASPEAAPVKLS----------GDDLKEANRKHMRSVFDFDLWKKHRSSSRYLRHIVGLGESRIVSGLMAPLTYVMTLSLAVACYNAAAEA-------------GYLP-VFPELKL--ATNAPFGLTSFALSLLLVFRTNSSYGRWDEARKMWGLIVNRS--RDFIRQGLGYIPPE-------------------QEELQ-----KMLVRWT----VAYSRSLMCHLRPGED-----LRVELKD-----TLKPEELEAL--LASTHR-------------PNYVVQVLTAIIKT----AQLPAAVTNNRDSTGCVPAGAAYRMDENLTVFADVTGGCERILRTPIPLSYTRH-TSRFMMIWLTLL-------PFTLWDSC-----HWAMLPIAGIVSFLLLGIEEIGVQIEEPFTI----LPLEVISRT-IEGNVWELYRMH

>Cre06.g261750.t1.2_(BST4)

M-----------------------QC--------QLKH----------------------------GA-RPQSQRPNWLPARAATLRPAVQHGVR---------------RGLTLGVKAAAAPLE--------DKKMPADMTTRQYRRVVYDFALWAKHRDVNRYLYNLRTIPGSRIIRQLSQPMGVVLAWAALFGFYETCLEA-------------GVLPSYLPKMTL--MSAEPQGLTSFALSLLLVFRTNSSYGRFDEARKIWGGILNRA--RNIANQAVTFIPAE-------------------DQAGR-----EAVGKWT----VGFTRALQAHLQEDID-----LRKELEKA--TPRWSKEEIDML--VNAQHR-------------PIKAISVLSELTRQ----LSITQF--------------QALQMQENCTFFYDALGGCERLLRTPIPVSYTRH-TARFLTIWLAML-------PLGLWERY-----HWSMLPVIALIGFLLLGIDEIGISIEEPFGI----LPLDAICGR-AQTDVNSLLKED

>XP_002945810.1_Volvox_carteri_f._nagariensis

M-----------------------QS--------QLQP----------------------------RL-QLQGTRLNWLPQRSCVQRRSLRVDAT---------------SG------AAPPPPA--------GKELSNDMVTRQYRRTVYDFSLWAKHRDVNRYLYNLKTIPGSRIIRTLGQPMGIVLAWAAMFGFYETCLES-------------GVLPSYFPKLTL--MSAEPQGLTSFALSLLLVFRTNSSYGRFDEARKIWGGILNRA--RNIANQAVTFIPAE-------------------DVAGR-----EAVGKWA----VGFCRALQAHLQEDAN-----LREELQKA--QPRWSREEIDML--CSAQHSWQQLQSCVNAFW-PIKAISMLSELTRQ----LPISQF--------------QALQMQENVTFFYDALGGCERLLRTPIPVSYTRI--------------------------------------------------------------------LPLDAICTR-AQTDVVSLLKDD

>Chrysochromulina_sp._(KOO32217.1)

M-----------------------REHPLSYEEYMRQRS----------AGRDPLA--EAVQGQSASM-ERGVVPPPPVKATPERTEAAFTPPAEFDFFQDVFKPTVESVVKAVVSPGAQQDSDESYMRVPWWEQGSTYSEDQRKDRRTVFMHDDWKRHRSSERFFRNIKTWPSSGINQALRKELTFVTSVSVFVVLANMLLYQYQDFGGVVHPGPLSFLDGPIKSLS---LPALPFSMASPVLSLLLVFRTNTAYFRWNEARTLWGGLINNC--RNIVRQTTTMFPNDA-----------------YHNALK-----KRLATET----ATFIKSLRNFLRGPEDDAT--LRKELYAYVNQGLMTSAQAEAT--LAAKNR-------------PMFALAAMSATLRK----ANIDEM--------------YISRMDSTISVLVDLTGANERIFKSPIPLVYTRL-TARFLSVFLTLL-------PLAMWAALGESWNHWATIPATFILSVFLFGVEEVGIQIEEPFSI----LPLEAMCNGAIEAVQLEMLAAE

**Supplemental Figure S31. Sequences used to generate Multiple Alignment using Fast Fourier Transform (MAFFT) alignments used to generate the phylogenetic trees included in this manuscript part 3 of 4.** FASTA files of MAFFT alignments of BST4 trimmed after the bestrophin domain BST4 and homologous amino acid sequences.

>Emiliania_huxleyi_(XP_005770556.1)

-----------------------------------------------------------------------------------------------------------------------------------------EFKEAGREFRQDVYSYNDWRWHRESGHIASAISSVFTSGVGKAMWRETFFVIATAAAVYLYNIGVPVLAAKTAASL----PIVAALLGRLPLLHLSLLPLTLSSPALFLLLVFRTNNSYDRWWEARKVWGGVINAS--RDLARQALALVR---------------------DAELK-----KLMVSQI----ASYARVLKYHLGPPTPEARDLLRNELVD----NRLPADQVRVI--MEAKHK-------------PMALLGLVSASLHDSGR-TGLDTV--------------QASKLDQTLSLLTDYLGKCERIVKTPLPLVYTRH-TARFLSWWLLFL-------PVCLYNQLRA---NWMIVPVSGLIGFFLVGIEDLGNQIEEPFSI----LPLTAMGTG-IQQSIFEAL---

>Phaeodactylum_tricornutum_(XP_002180738.1)

MM--------------------------------------------------------RNFASVLLLL-SSGAAAFAPVQHNGVRTIATPSTPLY---------------GNTKQPPALP--PIK----------DISYGEESRKYRRTVYSHDDWVKHRSSDRFLRNLLAIGSSGVYKSLAKEVLATTGVATFIVLYNCLVGGYTDLEGIKHS---ALIESVWAPLMA--LPLAPFTLSSPSLGLLLVFRTNTSYQRWDEARKNWGMNINHT--RDLVRMGTSFYDNAA-----------------VSSEQRAKDL-KALSLAT----WSFVRAMKRHLSPESEDEQD-FRRELFE-----RLPAPQAQAI--IDAAHR-------------PNRALFDLSVAIEN----LPMHFL--------------RKNQVHQAVTIFEDNLGSSERLLTSPVPLFYSRH-TARFLSFWLLLL-------PFALWDPFAGTWNHVGMIPATAVISIFLFGIEELATQMEEPFTI----LPMQAFCDK-IGNWCNEIVSWQ

>Thalassiosira_pseudonana_(XP_002289965.1)

M-----------------------------------------------------------------------------------------------------------------GPPIDPSVPVT-----------DQVGEGSRKYRRTVYTHDDWVRHRSPDRFGNNLSTLFNSGIYKQVANEVFATTAVATFVFLWNMIAGGYTDLAGVQHG---PIIDSPLAQMVG--LPMTAFTILTPSLGLLLVFRTNTSYGRWDEARKMWGLNINHT--RDLNRMATAWYGNEGNMDSVAFMGGDIPYSQPIDPVQRAYDL-GQVSLFT----WAFVRSMKRHLSPPEEDEED-FKAELRA-----RLTPEQAENI--INAAHR-------------PNRALFDLSVAIEN----LPMHFL--------------RKNAINTNLSIFEDTLGGCERLLSSPVPLFYSRH-TARFLSTWLLLL-------PFGLYEQFKDSWNHIAMIPATAFISVCLFGIEELATQLEEPFTI----LPMQGFCDK-IGGWCDEIVSW-

>WP_049046555.1_Kiebsiella_aerogenes

------------------------------------------------------------------------------------------------------------------------------------------------------------MIIRPEQHWFFRLFDWHGSVLSKIVFRLLLNVLMSVIAIISYQWYEQL-------------GI-----------HLTVAPFSLLGIAIAIFLGFRNSASYSRFVEARNLWGTVLIAE--RTLVRQLKNILPD--------------------DEETH-----KTLVSYL----VAFSWSLKHQLRK-TD-----PAVDLYR-----LLPKEKVAEI--LASSMP-------------TNRILLLIGNELGRLREQGKLSDI--------------TYGLMDNKLDELAHVLGGCERLASTPVPFAYTLI-LQRTVYLFCTLL-------PFALVGDL-----HYMTPFVSVFISYTFLSWDSLAEELEDPFGTSANDLPLNAMCNT-IERNLMDMTGQH

>XP_011543531.1_Homo_sapiens

M----------------------------------------------------------------------------------------------------------------------------------------------------TITYTSQVANARLGSFSRLLLCWRGSIYKLLYGEFLIFLLCYYIIRFIYRLALTEEQQL--------------MFEKLTL--YCDSYIQL--IPISFVLGFYVTLVVTRW------WNQYENLPWPDRLMSLVSGFVEGK-------------------DEQGRL--LRRTLIRYANLGNVLILRSVSTAVYKRFP-----SAQHLVQ---AGFMTPAEHKQLEKLSLPHNM---------FWVPWVWFANLSMKAWLG---GRIRDPI-------------LLQSLLNEMNTLRTQCGHLYAYDWISIPLVYTQVVTVAVYSFFLTCLVGRQFLNPAKAYPGHEL---DLVVPVFTFLQFFFYVGWLKVAEQLINPFGEDDDDFETNWIVDRNLQVSLLAVDEMH

**Supplemental Figure S32. Sequences used to generate Multiple Alignment using Fast Fourier Transform (MAFFT) alignments used to generate the phylogenetic trees included in this manuscript part 4 of 4.** FASTA files of MAFFT alignments of BST4 trimmed after the bestrophin domain BST4 and homologous amino acid sequences.

**Supplemental Figure S33**. **Light spectra of growth lights used in this work.** **A.** Spectrum of lights used for spot tests in Fig. 7 and S15. **B.** Light spectrum of Infors Multitron growth chamber used for liquid growth of cultures for Fig. 8 and S12, S13, S17-21.

**Supplemental Figure S33**. **Uncropped immunoblots from Supplemental Figure S3.** Blue and green boxes indicate where corresponding immunoblots were cropped to generate figures.

**Supplemental Figure S34**. **Uncropped immunoblots from Figures 2, 3, 4 and 8. A.** Uncropped immunoblot from Fig. 2**; B.** Fig. 3**; C.** Fig. 4 and **D.** Fig. 8. Blue, green and pink boxes indicate where corresponding immunoblots were cropped to generate figures. Dotted lines indicate where membranes were cut for incubation in different primary antibodies.

**Supplemental Figure S34**. **Uncropped immunoblots from Supplemental Figures S5, S14 and S16. A.** uncropped immunoblots from Fig. S5**; B.** Fig. S14 and **C.** Fig. S16. Blue, green and pink boxes indicate where corresponding immunoblots were cropped to generate figures. Dotted lines indicate where membranes were cut for incubation in different primary antibodies.

| **0.04% CO_2_** | | | | | | | |
| --- | --- | --- | --- | --- | --- | --- | --- |
|  |  | **Days 0-3** | | | **Days 0 to 5** | | |
|  | Strain | Mean | SE | *P(T<=t)*  two-tail | Mean | SE | *P(T<=t)*  two-tail |
| SGR  (μ h^-1^) | WT | 0.0389 | 0.0007 | 0.1652 | 0.0241 | 0.0010 | 0.2036 |
|  | *bst4* | 0.0402 | 0.0003 |  | 0.0257 | 0.0001 |  |
| Doubling time (h) | WT | 17.8462 | 0.3380 | 0.1669 | 28.8436 | 1.2467 | 0.2119 |
|  | *bst4* | 17.2379 | 0.1255 |  | 26.9798 | 0.1491 |  |
| **3% CO_2_** | | | | | | | |
|  |  | **Days 0-2** | | | **Days 0-3** | | |
|  | Strain | Mean | SE | *P(T<=t)*  two-tail | Mean | SE | *P(T<=t)*  two-tail |
| SGR  (μ h^-1^) | WT | 0.0679 | 0.0013 | 0.8626 | 0.0456 | 0.0011 | 0.9436 |
|  | *bst4* | 0.0682 | 0.0010 |  | 0.0455 | 0.0003 |  |
| Doubling time (h) | WT | 10.2145 | 0.1930 | 0.8555 | 15.2239 | 0.3725 | 0.9777 |
|  | *bst4* | 10.1669 | 0.1510 |  | 15.2353 | 0.0878 |  |

**Supplemental Table S1. Growth rate of *bst4* compared to WT.** Comparison of specific growth rates (μ h^-1^) and cell doubling times of wild type (WT) and *bst4* strains (n=3) during liquid growth assays at 0.04 and 3% CO_2_ (+/- 2 ppm).

| **For BST4 recombineering** | **Sequence** |
| --- | --- |
| BST4_Recomb_5' | TCTCAGAGGCCAACCGCCCGCGCACTCGGCCCGACTGGCGCAACCAGCTGGGAGATCTGGGTGGCTCCG |
| BST4_Recomb_3'_NoTag | CTGCCCGAGCTCTGAACCCCGGAGCCCGGCTGCAGAGCTCCAGCAGAGCCGAAGATCCTTTGATCTTTTCTACGGG |
| BST4_Recomb_3'_Tag | CAGAGGCCAACCGCCCGCGCACTCGGCCCGACTGGCGCAACCAGCTGTAATGGCAGCAGCTGGACC |
| BST4_Recomb_3'_Trunc | CAGTCATGAAGTACGTGGACGACGTGCGCTCAGGCCGGGTCAAGTCGTAATGGCAGCAGCTGGACC |
| **For generating Xenopus constructs** | **Sequence** |
| BST4_GW_Xen_F | GGGGACAAGTTTGTACAAAAAAGCAGGCTCATGTTGGGCGTTAAGGCTGC |
| BST4_GW_Xen_R_Full | GGGGACCACTTTGTACAAGAAAGCTGGGTACTTACAGCTGGTTGCGCCA |
| BST4_GW_Xen_R_Trunc | GGGGACCACTTTGTACAAGAAAGCTGGGTACTTACGACTTGACCCGGCCTGAGC |
| **For generating BST4 WR/EE mutations in BST4 disordered region for Y2H:** | **Sequence** |
| BST4dis 2x W/RtoE part II F | ttggtctcaAGACGTCCCGTTAATGAGGAGGACG |
| BST4dis 2x W/RtoE part II R | ggtctcaCGAAccCAACTGATTTTCCTCATCTGG |
| **For generating full length BST4 WR/EE** | **Sequence** |
| BST4 WRtoEE I F | ttgaagacaaAATGCAGTGCCAACTCAAACACG |
| BST4 WRtoEE I R | TTGAAGACAAGTGAGCCTAAAGTtTTCGCAACG |
| BST4 WRtoEE II F | TTGAAGACAATCACTGTTCACTAACGTAAGAGC |
| BST4 WRtoEE II R no stop | TTGAAGACAACGAACCCAACTGATTTTCCTCATCTGG |

**Supplemental Table S2. Primers used for cloning.** Primers are listed under subheadings for each vector type generated through PCR, as described in Materials & Methods section.
