# Supplementary material for: Bestrophin-like protein 4 is involved in photosynthetic acclimation to light fluctuations in Chlamydomonas
Source: Plant Physiol. 2024 Sep 6;196(4):2374–94. doi: 10.1093/plphys/kiae450 (PMC11638005; doi:10.1093/plphys/kiae450)
Supplement: kiae450_Supplementary_Data [file pp.01897.2023-s07.pdf › pp.01897.2023-s07.pdf]

Plant Physiology®

The role of BST4 in the pyrenoid of Chlamydomonas reinhardtii

Liat Adler, Chun Sing Lau, Kashif Shaikh, Kim van Maldegem, Alex Payne-Dwyer, Cécile Lefoulon, Philipp Girr, Nicky Atkinson, James Barrett, Tom Emrich-Mills, Emilija Dukic, Michael Blatt, Mark Leake, Gilles Peltier, Cornelia Spetea, Adrien Burlacot, Alistair McCormick, Luke Mackinder, and Charlotte Walker

Review Timeline:

Submission Date:	04-Dec-2023
Editorial Decision:	09-Jan-2024
Revision Received:	22-May-2024
Accepted:	01-Jul-2024

09-Jan-2024

Dr. Liat Adler
The University of Edinburgh School of Biological Sciences
Edinburgh
United Kingdom

RE: The role of BST4 in the pyrenoid of Chlamydomonas reinhardtii

Dear Dr. Adler,

We have received reviews of your manuscript, which you may view at the end of this letter. On the basis of the advice received and my own evaluation, I believe that, pending revision, this article may ultimately be acceptable for publication in Plant Physiology.

You will see that the reviewers agree that you have provided a substantial amount of work on the characteristics of a BST4 mutant. However, they suggest that your data interpretation requires revision. A key point is relates to the weak evidence for function of BST4 as a bicarbonate channel. The reviewers do not see a requirement for further experimentation but I suggest you look closely at all their points. I note that your manuscript received similar comments from The Plant Cell, so it is important that you fully address the interpretation of the function of BST4.

When you are ready to submit your revision, click the below link and find the red arrow under your Author Tasks heading for this revision submission.

If you want to visit this version of the manuscript, you may click the link below and then click on the folder that reads "Post Decision Manuscripts".

Link Not Available

When uploading the revised version of this article, please be sure to include as complete an answer as possible to the referees' criticisms during the first round of reviews in the "response to reviewers" section.

Plant Physiology considers a revision for up to 60 days after a manuscript has been provisionally accepted; limited extension of this timeframe is at the discretion of the Monitoring Editor. A reminder notice will be sent to you 2 weeks before the deadline, at which time you may choose to contact the editorial office to request an extension; if so, please provide a justification for your request. After the expiration of this deadline, the article would need to be submitted as a resubmission and would be handled as such.

Thank you very much for giving us an opportunity to review this work. I look forward to receiving the next version.

Yours sincerely,

Editor, Plant Physiology

Want to add this revision deadline to your calendar? Click below!

-- Reviewer comments:
Reviewer #1 (Comments for the Author):

Title: The role of BST4 in the pyrenoid of Chlamydomonas reinhardtii
By Adler et al.

This manuscript describes about the localization mechanism and function of the BST4 factor in the green alga, Chlamydomonas reinhardtii. BST4 is a putative anion channel that is estimated to be a factor playing a similar role to that of BST1-3, which are known to be HCO3- channel localized at the thylakoid membrane of Chlamydomonas. BST1-3 supply HCO3- to the lumen of

the pyrenoid tubule where an alfa carbonic anhydrase, CAH3 evolves CO2 to efflux towards Rubisco enzyme in the pyrenoid. As BST4 is a thylakoid-spanning protein and possesses a unique C-terminal extension harboring two Rubisco binding motifs, it was predicted to be a key molecule to recruit thylakoid membrane into the pyrenoid during the biogenesis of the pyrenoid.

In the present study, the authors demonstrated that BST4 is localized at the pyrenoid tubules forming pentameric structure, and further this pentameric unit may form higher order clusters. A heterologous expression of BST4 in the Arabidopsis resulted in the localization of this protein at the stroma lamella, but BST4 fail to tether thylakoid membrane into pyrenoid even if it is introduced into Arabidopsis transformant carrying hybrid Rubisco (S2cr) and EPYC1. BST4-defective line of Chlamydomonas did not lose the pyrenoid-tubule network. Given these results, the authors concluded that BST4 do not have a role to tether thylakoid membrane within the pyrenoid in either engineered land plants or Chlamydomonas even though BST4 can interact with Cr-type RbcS using the function of Rbc binding motif at its C-terminus in both Chlamydomonas and Arabidopsis transformant carrying hybrid AtRbcL/CrRbcS. The authors further demonstrated that, in Chlamydomonas transformant carrying CrRbcL/AtRbcS hybrid Rubisco (which is inert in forming pyrenoid), BST4-vinus still expressed at the location where pyrenoid should occur. The authors concluded that BST4-C-terminus is not only attracted by RbcS to be sorted to the pyrenoid but also is under a migration control of some unknown factors when it is targeted to the pyrenoid.

The BST4-defective mutant does not show slow growth as compared to the WT Chlamydomonas cell under a broad range of growth CO2 from 0.04 to 3%, suggesting that BST4 is not involved in the Chlamydomonas CCM. Noteworthy phenotypic characteristics of the bst4 KO mutant in comparison with WT were the higher H2O2 accumulation, higher Fv/Fm, NPQ, and VH+ values at steady state photosynthesis over 20 min of illumination. Also at short transient increase of NPQ within 1 min after dark to light transition was evident. The authors postulated from these results that the absence of BST4 in Chlamydomonas caused lower lumenal pH at the initial one min after shedding light to dark acclimated cells and prolonged growth in the light gave H2O2 production by constitutively higher light excitation pressure in mutant, and as a result of ROS stress, NPQ in mutant is also acclimated to the relatively higher level.

The localization mechanism of BST4 in this manuscript is clarified by solid methods and the conclusions drawn by the authors are sound. On the other hand, estimations for the function of CrBST4 by the authors are based on limited evidence and sounded quite leaky. In this regard, the manuscript still has a large room for the improvement. I had a strong impression that the function of CrBST4 is still an open question, thus cannot be concluded decisively into a simple context at current stage. Rather, the authors need to list possible mechanisms to explain the data, some of which may need additional experiment. Below I list specific comments.

1. L223-225: It is helpful to explain here why the authors introduced the bst4:tag gene into S2Cr line which does not harbor EPYC1. I assume that, by this experimental setting, the authors tried to figure out whether or not BST4 by itself has a function to tether Rubisco condensate and the pyrenoid tubules without the aid of EPYC1.

2. L270-271：If there are any precedential information about the BST orientation and the channel function, please refer in this part.

3. L353-357: This part will become easier to follow by reminding the contrast of two host system; one KO line of bst4 and the other WT. The introduction of truncated BST4 to the former cell line resulted in expression of the homo-truncated BST4 complex but in the latter cell the BST4 complex will be mix of truncated BST4 with WT BST4.

4. L353-357: Here the authors describe truncated BST4 is a one vinas-tagged but Fig. S5 says mScarlett-tag.

5. L361-378：FigS6 does not have control data. If there is any previous information about vinas expression in Chlamydomonas chloroplast, please quote here.

6. L361-378：Please provide basic information about the difference (or they can be structurally distinguishable) between Stroma thylakoid and pyrenoid tubules in the AtRbcS-expressing (pyrenoid-less) mutants. AtRbcS-expressing mutant line cannot make the pyrenoid structure but starch sheath is there at the original pyrenoid position, implying that there must be components that designate the position of the pyrenoid.

7. L450-45; L495-497; L505; L515-517and relating parts: It is assumed that carbon fixation and CCM of BST4 KO line grown under atmospheric CO2 are not significantly different as compared to WT, since growth profile of these two lines under 0.04% CO2 is similar. This means that the linear electron flow is also similar between BST4 KO and WT lines. However, both ROS and NPQ (at both initial transient and steady state under illumination) levels were significantly higher in the BST4 defective lines relative to the WT line. This means that both qE and qNO are significantly higher in bst4 defective lines even though ETR should be similar between mutants and WT. This implies that bst4 mutants should have higher energy input at PSII, while Fv/Fm data does not support this. The authors speculated that higher ROS levels in bst4 mutant lines are due to undetectable reduction of CCM activity in the bst4 mutant lines and ROS production might stimulate stress responses, which strengthen the NPQ, but again this is not supported by evidence. Decrease in the activities of CCM and ETR at a level causes ROS production (which means the level exceeding capacity of NPQ) under limited DIC would alter the interpretation of the reason why NPQ increased

at shorter and longer time scale, which cannot be simply ascribed to lumen pH.

8. L566-569: The speculation that BST4 transports HCO_3^- into lumen at the very initial min of dark-light transition and then BST1-3 will take over the HCO_3^- supply is not persuasive as cells under the dark rather develop intracellular CO_2 pool and a part of which would be hydrated into HCO_3^- under neutral pHs in the dark. It would be also possible that BST4 may channel other ions which could play an important role to reduce initial thylakoidal membrane potential (enhancing delta pH) at the initial dark-light transition, unless otherwise the cells cannot perform an efficient thylakoid function at the very begging in the light. There are many possibilities.

9. L555-579: This model is too closed. If BST4 is involved in the CCM and ETR at the level which produces ROS in the absence of BST4, difference in CCM and ETR relative to WT could also be at the measurable levels. Interpretation of the photosystem parameters are extremely complicated. Data should be addressed more thoroughly taking time scale and DIC and/or CO_2 concentrations at the measurement conditions into account, so that the several possibilities of the BST4 function can be overviewed as an open question.

Reviewer #2 (Comments for the Author):

Alder et al. characterized the bestrophin-like transmembrane protein, BST4. Their data showed that BST4 forms a complex that is localized to the pyrenoid tubules. A bst4 mutant of C. reinhardtii was not impaired in its growth at CO_2 in air. In addition, normal pyrenoid tubules were formed in the mutant. Remarkably, the NPQ content was higher in the bst4 mutant. In high light, the bst4 mutant showed persistently higher levels of NPQ and increased levels of light-induced H_2O_2 production. The authors hypothesize that BST4 is an ion channel involved in the regulation of pH in the lumen, possibly by mediating bicarbonate transport across the pyrenoid tubules. This work contains a considerable amount of work and high-quality data. However, the outlined hypothesis stands on shaky ground and is not fully supported by the data.

Other comments

1. There is no direct evidence that BST4 is involved in bicarbonate transport across the pyrenoid tubules. The difference in NPQ between bst4 and WT is considered indirect evidence. The authors state "By quantifying the non-photochemical quenching (NPQ) of chlorophyll fluorescence, we show that bst4 displays a transiently lower thylakoid lumenal pH during dark to light transition compared to control strains." However, NPQ is modulated by several factors. LHCSR3 levels are similar under moderate light. PMF, proton conductance and proton flux are not significantly different (Fig. 7), suggesting that the acidification of the lumen possibly does not differ between bst4 and WT. On the other hand, proton flux is significantly different in the high light experiment (Fig. S12). However, LHCSR3 levels are now increased in bst4, which could be a possible cause for the increased NPQ. Thus, the data do not support the conclusion that BST4 is involved in bicarbonate transport after dehydration to CO_2 in the lumen. Are parameters such a NPQ, PMF, proton conductance and proton flux sensitive to nigericin in WT, bst4 and rescue?

2. Acidification of the lumen causes photosynthetic control via the Cyt b6f complex, resulting in a slowdown PQH_2 oxidation and correspondingly in a slowdown of entire photosynthetic electron transfer. In this regard, is electron transfer measured as O_2 evolution or via ETR measurements e.g. via the dark pulse method slower in bst4 as compared to WT? In other words, is photosynthetic control different between WT, bst4 and rescue?

3. What about the PSII efficiency factors qP and qL?

4. In Fig. 6C, Chl a and b were measured and significant differences are seen between WT and bst4. What is the reason? Are LHC proteins down or photosystems?

5. Is H_2O_2 production also sensitive to DBMIB as it is to DCMU, to assess whether differences in the redox state of the PQ pool could be responsible for elevated H_2O_2 production in bst4?

6. Is CO_2 fixation different between WT, bst4 and rescue?

7. Is the photosynthetic phenotype and the NPQ phenotype also valid under CO_2-rich conditions?

Reviewer #3 (Comments for the Author):

Reviewers brief overview of the research: The protein BST4 (bestrophin-like protein 4, also known as Rubisco binding membrane protein 1, RBMP1; Cre06.g261750) localizes exclusively to the pyrenoid tubules and has been proposed to function to tether such tubules to the Rubisco matrix (Meyer et al., 2020; Science Advances). This suggestion was as a result of it binding to SAGA1, a ~180-kDa protein with a starch-binding domain that localizes to the periphery of the matrix, and is required for

normal pyrenoid morphology. However, BST4 also has a well-conserved bestrophin domain similar to those present in the thylakoid-localized BST1-3 proteins, which putatively function as anion (e.g. HCO_3^-) channels (Mukherjee et al. 2019, PNAS). Therefore, the authors primary aim is to show that BST4 indeed functions as an HCO_3^- channel for conversion to CO_2 in the thylakoid lumen for carbon assimilation.

Strengths of the study: The researchers have gone to great efforts to confirm the location of BST4 and show the necessity of the C-terminal RBM section of BST4 for the phenotype (if BST4 is still translated except without its C-terminal, then differences to wild-type in NPQ, H_2O_2 production rates, chlorophyll content, diffuse growth on agar are maintained). The structure of the pyrenoid tubules seem not to be disrupted in the bst4 knock-out mutant in support of a different function from that currently proposed in the literature, although possibly more could be shown in support of this then single-slice electron micrographs (e.g. 3D reconstruction). I also read the section on elevated H_2O_2 production and phototaxis with interest. The authors show that phototaxis is enhanced in bst4 in liquid and on agar, alongside higher H_2O_2 production rates. However, regarding interpretation of the H_2O_2 results (line 452) there are contrasting results published to those stated, clearly showing that restricted CO_2 availability in photoautotrophic conditions significantly lowers H_2O_2 production in Chlamydomonas coincidently when qE-type NPQ increases (Roach et al, 2015, Plant Journal). Therefore, a low luminal pH and accompanied photosynthetic control and qE most likely lowers rates of the Mehler reaction. Could there be another explanation for the higher H_2O_2 production in bst4? The authors could conclude that the role of BST4 in photosynthesis-associated ROS production warrants further exploration.

Weaknesses of the study: The authors suggest that HCO_3^- transport is disrupted in bst4 mutant in the first 2 min of dark to high light transition, affecting luminal pH. An overriding weakness of the functional aspect of the study is what the authors are trying to show has not been directly measured, and only inferred indirectly from measurements of processes affected by a change in pH of the thylakoid lumen, namely NPQ. This become complicated by the multiple ways in which chlorophyll fluorescence in Chlamydomonas is affected during kinetic measurements used to measure NPQ. Also, lack of BST4 leads to a decrease in chlorophyll contents / cell, which suggests it has smaller antenna. This result (ca. 40% chlorophyll reduction) is significant but hasn't seem to have been further studied. Unfortunately, an expression system in oocytes was unable to confirm a channel function for BST4. Therefore, assumption of an HCO_3^- channel and influence of luminal pH must currently be viewed as speculative, especially since the electrochromic shift (ECS) measurements did not reveal differences in the ΔpH between wild-type and bst4. On Line 503 it seems assumed that "the luminal pH was higher in bst4 mutant", but this has not been directly shown. Here, the authors could potentially use 9-aminoacridine fluorescence to support this statement.

Although the authors very nicely show that the unique C-terminal of the BST4 sequence that is not found in BST1-3 is critical for the phenotype, questions remain about the exact coordination of BST1-3 and BST4 activities described in their model in Fig. 8. The authors could include the bst1-3 mutants in the kinetic analyses of NPQ to support their model shown. Why is it that BST4 should be only or more active as an HCO_3^- channel in the first few minutes from dark to high light and not BST1-3, and does this confer a particular advantage to the cells, since it does not seem to under constant light? Considering kinetic measurements show an NPQ phenotype in bst4 between 1-2 min after a dark to high light transition, one could imagine there could be an associated growth phenotype under fluctuating light, but such growth tests have not been shown.

Other / minor:

At Line 492, it would be clearer if "in the dark" was written after "quickly dissipated".

Line 524: to = too

Potentially, Fig.4 could be moved forward to Fig. 2, since this result asks the question of BST4 function if not in tubule structure.

Growth tests of bst4 at ambient CO_2 were surprisingly indifferent to wild type. Is there an explanation especially considering the large difference in chlorophyll contents?

RE: The role of BST4 in the pyrenoid of Chlamydomonas reinhardtii

Dear Dr. Adler,

We have received reviews of your manuscript, which you may view at the end of this letter. On the basis of the advice received and my own evaluation, I believe that, pending revision, this article may ultimately be acceptable for publication in Plant Physiology.

You will see that the reviewers agree that you have provided a substantial amount of work on the characteristics of a BST4 mutant. However, they suggest that your data interpretation requires revision. A key point is relates to the weak evidence for function of BST4 as a bicarbonate channel. The reviewers do not see a requirement for further experimentation but I suggest you look closely at all their points. I note that your manuscript received similar comments from The Plant Cell, so it is important that you fully address the interpretation of the function of BST4.

When you are ready to submit your revision, click the below link and find the red arrow under your Author Tasks heading for this revision submission.

If you want to visit this version of the manuscript, you may click the link below and then click on the folder that reads "Post Decision Manuscripts".

https://pphys.msubmit.net/cgi-bin/main.plex?el=A6It4Non1A1BDYh7F4A9ftdPPPVjJo3SxunFplrl96x5QZ

When uploading the revised version of this article, please be sure to include as complete an answer as possible to the referees' criticisms during the first round of reviews in the "response to reviewers" section.

Plant Physiology considers a revision for up to 60 days after a manuscript has been provisionally accepted; limited extension of this timeframe is at the discretion of the Monitoring Editor. A reminder notice will be sent to you 2 weeks before the deadline, at which time you may choose to contact the editorial office to request an extension; if so, please provide a justification for your request. After the expiration of this deadline, the article would need to be submitted as a resubmission and would be handled as such.

Thank you very much for giving us an opportunity to review this work. I look forward to receiving the next version.

Yours sincerely,

Editor, Plant Physiology

Want to add this revision deadline to your calendar? Click below!

-- Reviewer comments:
Reviewer #1 (Comments for the Author):

Title: The role of BST4 in the pyrenoid of Chlamydomonas reinhardtii
By Adler et al.

This manuscript describes about the localization mechanism and function of the BST4 factor in the green alga, Chlamydomonas reinhardtii. BST4 is a putative anion channel that is estimated to be a factor playing a similar role to that of BST1-3, which are known to be HCO3- channel localized at the thylakoid membrane of Chlamydomonas. BST1-3 supply HCO3- to the lumen of the pyrenoid tubule where an alfa carbonic anhydrase, CAH3 evolves CO2 to efflux towards Rubisco enzyme in the pyrenoid. As BST4 is a thylakoid-spanning protein and possesses a unique C-terminal extension harboring two Rubisco binding motifs, it was predicted to be a key molecule to recruit thylakoid membrane into the pyrenoid during the biogenesis of the pyrenoid.

In the present study, the authors demonstrated that BST4 is localized at the pyrenoid tubules forming pentameric structure, and further this pentameric unit may form higher order clusters. A heterologous expression of BST4 in the Arabidopsis resulted in the localization of this protein at the stroma lamella, but BST4 fail to tether thylakoid membrane into pyrenoid even if it is introduced into Arabidopsis transformant carrying hybrid Rubisco (S2cr) and EPYC1. BST4-defective line of Chlamydomonas did not lose the pyrenoid-tubule network. Given these results, the authors concluded that BST4 do not have a role to tether thylakoid membrane within the pyrenoid in either engineered land plants or Chlamydomonas even though BST4 can interact with Cr-type RbcS using the function of Rbc binding motif at its C-terminus in both Chlamydomonas and Arabidopsis transformant carrying hybrid AtRbcL/CrRbcS. The authors further demonstrated that, in Chlamydomonas transformant carrying CrRbcL/AtRbcS hybrid Rubisco (which is inert in forming pyrenoid), BST4-vinus still expressed at the location where pyrenoid should occur. The authors concluded that BST4-C-terminus is not only attracted by RbcS to be sorted to the pyrenoid but also is under a migration control of some unknown factors when it is targeted to the pyrenoid.

The BST4-defective mutant does not show slow growth as compared to the WT Chlamydomonas cell under a broad range of growth CO2 from 0.04 to 3%, suggesting that BST4 is not involved in the Chlamydomonas CCM. Noteworthy phenotypic characteristics of the bst4 KO mutant in comparison with WT were the higher H2O2 accumulation, higher Fv/Fm, NPQ, and VH+ values at steady state photosynthesis over 20 min of illumination. Also at short transient increase of NPQ within 1 min after dark to light transition was evident. The authors postulated from these results that the absence of BST4 in Chlamydomonas caused lower lumenal pH at the initial one min after shedding light to dark acclimated cells and prolonged growth in the light gave H2O2 production by constitutively higher light excitation pressure in mutant, and as a result of ROS stress, NPQ in mutant is also acclimated to the relatively higher level.

The localization mechanism of BST4 in this manuscript is clarified by solid methods and the conclusions drawn by the authors are sound. On the other hand, estimations for the function of CrBST4 by the authors are based on limited evidence and sounded quite leaky. In this regard, the manuscript still has a large room for the improvement. I had a strong impression that the function of CrBST4 is still an open question, thus cannot be concluded decisively into a simple context at current stage. Rather, the authors need to list possible mechanisms to explain the data, some of which may need additional experiment. Below I list specific comments.

We thank the reviewer for appreciating our work, we acknowledge that the function of BST4 is still an open question and we now provide in the new version of the manuscript a more open model on the putative role that it might play (lines 529-533).

1. L223-225: It is helpful to explain here why the authors introduced the bst4:tag gene into S2Cr line which does not harbor EPYC1. I assume that, by this experimental setting, the authors tried to figure out whether or not BST4 by itself has a function to tether Rubisco condensate and the pyrenoid tubules without the aid of EPYC1.

We used $S2_{Cr}$ alone without EPYC1 present to first assess the expression and localisation of BST4 without a Rubisco condensate present. We have altered **lines 201-203** to clarify this point: "We initially expressed a BST4-mNeon fusion protein in $S2_{Cr}$ without EPYC1 to first confirm correct localization of BST4 to thylakoids without a Rubisco condensate present"

2. L270-271 : If there are any precedential information about the BST orientation and the channel function, please refer in this part.
BST4 is predicted to be oriented with the C-terminus facing the stroma since parts of the protein that face the stroma tend to have a higher frequency of positively charged amino acids (Gavel et al., 1991); this has been added to the text on **lines 219-221** along with **Fig. S4.**

3. L353-357: This part will become easier to follow by reminding the contrast of two host system; one KO line of bst4 and the other WT. The introduction of truncated BST4 to the former cell line resulted in expression of the homo-truncated BST4 complex but in the latter cell the BST4 complex will be mix of truncated BST4 with WT BST4.

We agree that this concept should be made clearer. We have modified **lines 312-318** to now read: "When $BST4_{\Delta C\text{-term}}$-Venus was expressed in WT Chlamydomonas (i.e., still expressing the native version of BST4), we observed fluorescence throughout the thylakoids, but with the majority of the signal still localized to the pyrenoid tubules (**Fig. S8**). This is in contrast to $BST4_{\Delta C\text{-term}}$-mScarlet expressed in the *bst4* mutant where, presumably, only homo truncated pentamers are formed and all the signal was mislocalized to the thylakoid membranes proper. We conclude that $BST4_{\Delta C\text{-term}}$-Venus is recruited to the pyrenoid through heteromeric pentamers constituting truncated and native full-length BST4, which is further evidence that BST4 oligomerizes."

4. L353-357: Here the authors describe truncated BST4 is a one vinas-tagged but Fig. S5 says mScarlett-tag.
This has been corrected to Venus-tagged in what is now **Fig. S8.**

5. L361-378 : FigS6 does not have control data. If there is any previous information about vinas expression in Chlamydomonas chloroplast, please quote here.
Venus is commonly used to C-terminally tag proteins in Chlamydomonas. Venus alone localizes to the cytosol (Mackinder et al., 2017). We have included this reference in the legend of what is now **Fig S9**.

6. L361-378 : Please provide basic information about the difference (or they can be structurally distinguishable) between Stroma thylakoid and pyrenoid tubules in the AtRbcS-expressing (pyrenoid-less) mutants. AtRbcS-expressing mutant line cannot make the pyrenoid structure but starch sheath is there at the original pyrenoid position, implying that there must be components that designate the position of the pyrenoid.
Stromal thylakoids are laminar whereas pyrenoid tubules are reticulated. In AtRbcS expressing pyrenoid-less mutants there is indeed a nascent pyrenoid tubule structure that likely designates the position of the pyrenoid. To help differentiate this in the text, we have included 'unlike the laminar arrangement of stromal thylakoids' when describing the reticulated tubule network **lines 325-326**.

7. L450-45; L495-497; L505; L515-517and relating parts: It is assumed that carbon fixation and CCM of BST4 KO line grown under atmospheric CO2 are not significantly different as compared to WT, since growth profile of these two lines under 0.04% CO2 is similar. This means that the linear electron flow is also similar between BST4 KO and WT lines. However, both ROS and NPQ (at both initial transient and steady state under illumination) levels were significantly higher in the BST4 defective lines relative to the WT line. This means that both qE and qNO are significantly higher in bst4 defective lines even though ETR should be similar between mutants and WT. This implies that bst4 mutants should have higher energy input at PSII, while Fv/Fm data does not support this.

As the reviewer points out, our data does not support an increased PSII energy input in the *bst4* mutant. However, because in Chlamydomonas, a large proportion of electrons generated at the PSII level is dedicated to alternative electron transport (Burlacot et al, 2022; Peltier et al, 2023; Burlacot 2023), a change in the redistribution of the electron transport in *bst4* mutant could lead to a similar growth even with lower energy input at the PSII for *bst4*. While such quantifications are out of the scope of this study, we have modified the manuscript to reflect this possibility **lines 422-424**.

We also found Y(NO) is lower in *bst4* and the truncated complemented line compared to WT (**Fig S12,13**).

The authors speculated that higher ROS levels in bst4 mutant lines are due to undetectable reduction of CCM activity in the bst4 mutant lines and ROS production might stimulate stress responses, which strengthen the NPQ, but again this is not supported by evidence. Decrease in the activities of CCM and ETR at a level causes ROS production (which means the level exceeding capacity of NPQ) under limited DIC would alter the interpretation of the reason why NPQ increased at shorter and longer time scale, which cannot be simply ascribed to lumen pH.

We agree that the increase in NPQ after long time scales of illumination cannot be ascribed simply to lumenal pH. However, for short timescales of illumination (i.e. less than one minute), we have done additional experiments using the proton uncoupler nigericin (**Fig. S20**) which strongly supports that lumenal protons are mostly responsible for the transient NPQ increase in bst4 mutants after one minute of strong illumination. Our interpretation for changes in ROS production impacting NPQ levels, which still holds for longer timescales, is backed by ROS measurements (**Fig. S11**). We have discussed a possible explanation for longer term acclimations in the *bst4* mutant during prolonged exposure to high light in **lines 433-437**.

8. L566-569: The speculation that BST4 transports HCO3- into lumen at the very initial min of dark-light transition and then BST1-3 will take over the HCO3- supply is not persuasive as cells under the dark rather develop intracellular CO2 pool and a part of which would be hydrated into HCO3- under neutral pHs in the dark. It would be also possible that BST4 may channel other ions which could play an important role to reduce initial thylakoidal membrane potential (enhancing delta pH) at the initial dark-light transition, unless otherwise the cells cannot perform an efficient thylakoid function at the very begging in the light. There are many possibilities.

We acknowledge that there could be other explanations for the observed differences in initial NPQ induction and have opened our initial hypothesis of BST4 being a bicarbonate channel to just “an ion channel” **lines 529-533**. However, we want to note here that the majority of CO_2 production in the dark happens in the mitochondria, which will likely be converted directly into HCO_3^- in the mitochondrial matrix by the carbonic anhydrases CAH4/CAH5 (Rai et al, 2021, Plant Physiol), and so far there are no data showing that HCO3- could be shuttled out of the mitochondria in the dark (Findinier et al, 2024, BioRxiv).

9. L555-579: This model is too closed. If BST4 is involved in the CCM and ETR at the level which produces ROS in the absence of BST4, difference in CCM and ETR relative to WT could also be at the measurable levels. Interpretation of the photosystem parameters are extremely complicated. Data should be addressed more thoroughly taking time scale and DIC and/or CO2 concentrations at the measurement conditions into account, so that the several possibilities of the BST4 function can be overviewed as an open question.

We agree that the interpretation of photosynthetic parameters are not straightforward and that our proposed model is still a hypothesis. We have removed our claim that BST4 may be a bicarbonate channel. Instead have a more tentative conclusion that BST4 is likely functioning as an ion channel and state further work is needed to determine what species BST4 is transporting (**lines 529-533**).

Reviewer #2 (Comments for the Author):

Alder et al. characterized the bestrophin-like transmembrane protein, BST4. Their data showed that BST4 forms a complex that is localized to the pyrenoid tubules. A bst4 mutant of C. reinhardtii was not impaired in its growth at CO2 in air. In addition, normal pyrenoid tubules were formed in the mutant. Remarkably, the NPQ content was higher in the bst4 mutant. In high light, the bst4 mutant showed persistently higher levels of NPQ and increased levels of light-induced H2O2 production. The authors hypothesize that BST4 is an ion channel involved in the regulation of pH in the lumen, possibly by mediating bicarbonate transport across the pyrenoid tubules. This work contains a considerable amount of work and high-quality data. However, the outlined hypothesis stands on shaky ground and is not fully supported by the data.

We thank the reviewer for their appreciation of our work and agree that the exact role of BST4 still needs some clarification. We have modified our manuscript to a more conservative hypothesis line 529-533.

Other comments

1. There is no direct evidence that BST4 is involved in bicarbonate transport across the pyrenoid tubules. The difference in NPQ between bst4 and WT is considered indirect evidence. The authors state "By quantifying the non-photochemical quenching (NPQ) of chlorophyll fluorescence, we show that bst4 displays a transiently lower thylakoid lumenal pH during dark to light transition compared to control strains." However, NPQ is modulated by several factors. LHCSR3 levels are similar under moderate light. PMF, proton conductance and proton flux are not significantly different (Fig. 7), suggesting that the acidification of the lumen possibly does not differ between bst4 and WT. On the other hand, proton flux is significantly different in the high light experiment (Fig. S12). However, LHCSR3 levels are now increased in bst4, which could be a possible cause for the increased NPQ. Thus, the data do not support the conclusion that BST4 is involved in bicarbonate transport

after dehydration to CO2 in the lumen. Are parameters such a NPQ, PMF, proton conductance and proton flux sensitive to nigericin in WT, bst4 and rescue?
We have amended 'show' to 'propose' in the abstract on **line 77**. We have removed the model of bicarbonate transport from **line 80** and simply state our hypothesis that BST4 is functioning as an ion channel. We have now included an experiment with the proton-uncoupler nigericin which shows that the transient increase in NPQ in bst4 is nigericin dependent (**Fig S20**).

2. Acidification of the lumen causes photosynthetic control via the Cyt b6f complex, resulting in a slowdown PQH2 oxidation and correspondingly in a slowdown of entire photosynthetic electron transfer. In this regard, is electron transfer measured as O2 evolution or via ETR measurements e.g. via the dark pulse method slower in bst4 as compared to WT? In other words, is photosynthetic control different between WT, bst4 and rescue?

We thank the reviewer for this suggestion and agree that assessment of the photosynthetic control would have been a great addition to the manuscript. However, we did not have access to gross O_2 measurements to properly quantify ETR in our context. Additionally, as estimation of the ETR via the DIRK method is strongly affected by the ion leak through the thylakoid membrane (Bailleul et al, 2010, Phot Res), we could not use it reliably in the *bst4* mutant.

3. What about the PSII efficiency factors qP and qL?
We have included qP and 1-qL parameters in **Fig. S12** and **S13**. qP is slightly higher in *bst4* and 1-qL is slightly lower. A lower 1-qL could suggest a limitation in the PSI acceptor side, or more PSII reaction centers being closed, which may explain the increase in ROS generation. We have discussed this in the text **lines 397-407**.

4. In Fig. 6C, Chl a and b were measured and significant differences are seen between WT and bst4. What is the reason? Are LHC proteins down or photosystems?

We suspect this is an adaptation to real or perceived light stress and have included this in the text on **line 410** and **435-437**.

5. Is H2O2 production also sensitive to DBMIB as it is to DCMU, to assess whether differences in the redox state of the PQ pool could be responsible for elevated H2O2 production in bst4?

We thank the reviewer for this suggestion and discuss it in the manuscript **lines 397-407**.

6. Is CO2 fixation different between WT, bst4 and rescue?
Although we have not measured this, we suspect that there is a small or negligible difference, since there is no difference in growth. However, we agree this could be a reason that reducing energy is too high in the cells.

7. Is the photosynthetic phenotype and the NPQ phenotype also valid under CO2-rich conditions?

In the dot assay, we still observed the diffuse colony phenotype at 3% CO_2 and high light (**Fig. 6B**) suggesting that the ROS-production photosynthetic phenotype persists under CO_2

rich conditions but only under sufficiently high light. When low CO_2 grown cells were supplemented with bicarbonate at the start of dark adaptation, there was still a difference in NPQ induction (**Fig. S18**). However, our approach for evaluating lumenal pH would not work in cells adapted to high CO_2 as LHCSR3 is not expressed in high CO_2 in our growing conditions.

Reviewer #3 (Comments for the Author):

Reviewers brief overview of the research: The protein BST4 (bestrophin-like protein 4, also known as Rubisco binding membrane protein 1, RBMP1; Cre06.g261750) localizes exclusively to the pyrenoid tubules and has been proposed to function to tether such tubules to the Rubisco matrix (Meyer et al., 2020; Science Advances). This suggestion was as a result of it binding to SAGA1, a ~180-kDa protein with a starch-binding domain that localizes to the periphery of the matrix, and is required for normal pyrenoid morphology. However, BST4 also has a well-conserved bestrophin domain similar to those present in the thylakoid-localized BST1-3 proteins, which putatively function as anion (e.g. HCO3-) channels (Mukherjee et al. 2019, PNAS). Therefore, the authors primary aim is to show that BST4 indeed functions as an HCO3- channel for conversion to CO2 in the thylakoid lumen for carbon assimilation.

Strengths of the study: The researchers have gone to great efforts to confirm the location of BST4 and show the necessity of the C-terminal RBM section of BST4 for the phenotype (if BST4 is still translated except without its C-terminal, then differences to wild-type in NPQ, H2O2 production rates, chlorophyll content, diffuse growth on agar are maintained). The structure of the pyrenoid tubules seem not to be disrupted in the bst4 knock-out mutant in support of a different function from that currently proposed in the literature, although possibly more could be shown in support of this then single-slice electron micrographs (e.g. 3D reconstruction). I also read the section on elevated H2O2 production and phototaxis with interest. The authors show that phototaxis is enhanced in bst4 in liquid and on agar, alongside higher H2O2 production rates. However, regarding interpretation of the H2O2 results (line 452) there are contrasting results published to those stated, clearly showing that restricted CO2 availability in photoautotrophic conditions significantly lowers H2O2 production in Chlamydomonas coincidently when qE-type NPQ increases (Roach et al, 2015, Plant Journal). Therefore, a low luminal pH and accompanied photosynthetic control and qE most likely lowers rates of the Mehler reaction. Could there be another explanation for the higher H2O2 production in bst4? The authors could conclude that the role of BST4 in photosynthesis-associated ROS production warrants further exploration.

We agree that the enhanced NPQ and acidified lumen make the Mehler reaction an unlikely source of the H_2O_2. We have added this to the manuscript in **lines 431-433**.

Although the cell migration, which we attribute to ROS, is reduced under high CO_2 levels, we did not directly test the effect of CO_2 availability on the level of ROS production. The growth test was performed over several days at 150 μE, rather than 250 μE for 1 h (as described in Roach et al., 2015), which may explain the discrepancy.

We agree the source of H_2O_2 warrants further exploration and have discussed the different possibilities in **lines 397-407**.

Weaknesses of the study: The authors suggest that HCO3- transport is disrupted in bst4 mutant in the first 2 min of dark to highlight transition, affecting luminal pH. An overriding weakness of the functional aspect of the study is what the authors are trying to show has not

been directly measured, and only inferred indirectly from measurements of processes affected by a change in pH of the thylakoid lumen, namely NPQ. This become complicated by the multiple ways in which chlorophyll fluorescence in Chlamydomonas is affected during kinetic measurements used to measure NPQ. Also, lack of BST4 leads to a decrease in chlorophyll contents / cell, which suggests it has smaller antenna. This result (ca. 40% chlorophyll reduction) is significant but hasn't seem to have been further studied. Unfortunately, an expression system in oocytes was unable to confirm a channel function for BST4. Therefore, assumption of an HCO3- channel and influence of luminal pH must currently be viewed as speculative, especially since the electrochromic shift (ECS) measurements did not reveal differences in the ΔpH between wild-type and bst4. On Line 503 it seems assumed that "the luminal pH was higher in bst4 mutant", but this has not been directly shown. Here, the authors could potentially use 9-aminoacridine fluorescence to support this statement.

We agree that our models are speculative and have adjusted our text to reflect this in **lines 529-533**. We have also mentioned the possibility of decreased antenna size on **line 410** and **435**.

We have added an experiment to measure nigericin sensitive NPQ which shows the increased NPQ observed in bst4 is entirely nigericin dependent (**Fig. S20**)

Although the authors very nicely show that the unique C-terminal of the BST4 sequence that is not found in BST1-3 is critical for the phenotype, questions remain about the exact coordination of BST1-3 and BST4 activities described in their model in Fig. 8. The authors could include the bst1-3 mutants in the kinetic analyses of NPQ to support their model shown. Why is it that BST4 should be only or more active as an HCO3- channel in the first few minutes from dark to high light and not BST1-3, and does this confer a particular advantage to the cells, since it does not seem to under constant light? Considering kinetic measurements show an NPQ phenotype in bst4 between 1-2 min after a dark to high light transition, one could imagine there could be an associated growth phenotype under fluctuating light, but such growth tests have not been shown.

Thank you for this suggestion. We performed a fluctuating light spot test and found that *bst4* had a growth defect under fluctuating high light conditions. We have included this in the manuscript in **Fig. 7** and **Fig. S15** and have discussed in the text **lines 438-452**.

To test the effect of the genetic background on this phenotype, we also assessed two new CRISPR-generated knockout lines: *bst4-2* and *bst4-3*. We found that they are not as sensitive to fluctuating light as the bst4 CLiP mutant. *Bst4-2* displayed a weak growth defect and *bst4-3* had no difference in growth compared to WT. The weak phenotype of these newly generated mutants parallels a weak transient NPQ phenotype. We discuss this in the text **lines 452-460**.

We thank the reviewer for the useful suggestion of including BST1-3 mutants for further analysis to test the bicarbonate uptake model. However, we feel this is beyond the scope of this manuscript.

Other / minor:

At Line 492, it would be clearer if "in the dark" was written after "quickly dissipated".
Done, **line 476** in new document

Line 524: to = too
We have altered the sentence to improve readability. **Line 511** in revised document.

Potentially, Fig.4 could be moved forward to Fig. 2, since this result asks the question of BST4 function if not in tubule structure.

We feel that **Fig. 3** is an important precursor to **Fig. 4** since it determines that BST4 is in the thylakoid membranes of plants.

Growth tests of bst4 at ambient CO2 were surprisingly indifferent to wild type. Is there an explanation especially considering the large difference in chlorophyll contents?

Because the assay was done under saturating light conditions for growth on plates (150µE) we interpret that the growth is not limited by light harvesting ability, as cells will have sufficient light energy regardless of antenna size.

01-Jul-2024
Dr. Liat Adler
The University of Edinburgh School of Biological Sciences
Edinburgh
United Kingdom

MSID: PP2023-RA-01897R1
MS TITLE: The role of BST4 in the pyrenoid of Chlamydomonas reinhardtii

Dear Dr. Adler,

I am pleased to inform you that your revised manuscript "The role of BST4 in the pyrenoid of Chlamydomonas reinhardtii" has been accepted for publication in Plant Physiology. Your manuscript will be checked for consistency with journal formatting and image standards by one of our Science Editors, who will contact you shortly. They may have a number of queries and suggestions for you. Please keep in mind that they work to increase the online visibility of your article and its attraction for readers from the widest of backgrounds. You will have the opportunity to review any revisions, and I hope you will work constructively with them to get the most out of your research. You will also have the opportunity to review all changes when you receive the author proofs for your article.

Once validated for publication, your article will be sent to a Science Editor for a final review of your manuscript and source files. After your final files are uploaded, your article will appear in the next available issue and the online Preview version of your article will be posted after your signed Copyright and Checklist forms and final source files are received.

Please be sure to include the following in your manuscript file:
Harvard style citations
List of all author contributions and funding information at the beginning of manuscript after the titles and author list.
List all supplemental materials associated with the article, with titles, at the end of the ms, before the references and acknowledgments section.
Complete Figure Legends for all figures appearing in the article listed before the Literature Cited.

Thank you and congratulations on your Plant Physiology paper!

Sincerely,

Editor, Plant Physiology

=====
FIRST AUTHOR PROFILES
=====

We recognize the hard work that goes into being First Author of a Plant Physiology paper by publishing a "First Author Feature" for each First Author alongside your paper.

If the first author(s) would like to submit information for a feature on Facebook and Twitter, fill out the form by clicking on the link below:

Plant Physiology First Author Feature Form: https://forms.office.com/r/ZBCGZfhm8F
If you cannot access the form through the link above, please contact Rachel at rbelsky@aspb.org.

To upload an image of yourself to accompany your profile, please put your photo in the correct journal folder at the link below. You must label your image: Author First Name Last Name Article Title.

First Author Profile Image Dropbox: https://www.dropbox.com/scl/fo/xz029p7qjr2jml2rp2kmq/h?dl=0&rlkey=6fkxcaqmxx35ap152b2osy1fz

=====
IMPORTANT REMINDER: PEER REVIEW REPORTS

=====

If you opted to publish a peer review report along with your article during the original submission process, it will be prepared by the editorial staff and publicly posted with your manuscript, inside the zip file that contains any other supplemental material. As a reminder, the peer review report is a public record of all comments from editors and reviewers, as well as your prior responses, as you received them in the decision letters for each draft of your manuscript. If you agreed to publish this report and have changed your mind, or are not sure if you selected this option, please contact the editorial office as soon as possible before signing the license agreement from our publisher.

-- Reviewer comments:
Reviewer #1 (Comments for the Author):

The authors have responded thoroughly to three reviewers concerns and have improved the manuscript, part of which included supporting data from new experiments. The authors have carried out a proton ionophore experiment clearly showing the proton gradient dependence of initial NPQ rise in bst4 mutant. The authors also now well documented potential roles of BST4 in a manner as an open question. I do not have further comments to be added.

Reviewer #2 (Comments for the Author):

The revised manuscript is significantly improved and can thus be accepted.

Reviewer #3 (Comments for the Author):

Since the authors have shifted the emphasis away from a bicarbonate channel and considered or suitably discussed the critical points of the reviewer, I think the paper is suitable for publication.
